# High-Resolution Confocal Fluorescence Imaging of Serine Hydrolase Activity in Cryosections – Application to Glioma Brain Unveils Activity Hotspots Originating from Tumor-Associated Neutrophils

**DOI:** 10.1186/s12575-020-00118-4

**Published:** 2020-03-15

**Authors:** Niina Aaltonen, Prosanta K. Singha, Hermina Jakupović, Thomas Wirth, Haritha Samaranayake, Sanna Pasonen-Seppänen, Kirsi Rilla, Markku Varjosalo, Laura E. Edgington-Mitchell, Paulina Kasperkiewicz, Marcin Drag, Sara Kälvälä, Eemeli Moisio, Juha R. Savinainen, Jarmo T. Laitinen

**Affiliations:** 1 Institute of Biomedicine, University of Eastern Finland (UEF), POB 1627, FI-70211 Kuopio, Finland; 2 Aurealis Pharma, Kuopio, Finland; 3 Institute of Biotechnology & HiLIFE, University of Helsinki, Helsinki, Finland; 4 Department of Biochemistry and Molecular Biology, Bio21 Molecular Science and Biotechnology Institute, The University of Melbourne, Parkville, VIC Australia; 5 Drug Discovery Biology, Monash Institute of Pharmaceutical Sciences, Monash University, Parkville, VIC Australia; 6 Department of Oral and Maxillofacial Surgery, New York University College of Dentistry, Bluestone Center for Clinical Research, New York, NY USA; 7 Department of Bioorganic Chemistry, Wroclaw University of Science and Technology, Wroclaw, Poland

**Keywords:** Activity-based protein profiling (ABPP), Brain cryosection, Glioblastoma multiforme (GBM), Immunohistochemistry, Serine hydrolase activity, Neutrophil serine protease (NSP), TAMRA-FP probe, Tumor-associated neutrophils

## Abstract

**Background:**

Serine hydrolases (SHs) are a functionally diverse family of enzymes playing pivotal roles in health and disease and have emerged as important therapeutic targets in many clinical conditions. Activity-based protein profiling (ABPP) using fluorophosphonate (FP) probes has been a powerful chemoproteomic approach in studies unveiling roles of SHs in various biological systems. ABPP utilizes cell/tissue proteomes and features the FP-warhead, linked to a fluorescent reporter for in-gel fluorescence imaging or a biotin tag for streptavidin enrichment and LC-MS/MS-based target identification. Existing ABPP approaches characterize global SH activity based on mobility in gel or MS-based target identification and cannot reveal the identity of the cell-type responsible for an individual SH activity originating from complex proteomes.

**Results:**

Here, by using an activity probe with broad reactivity towards the SH family, we advance the ABPP methodology to glioma brain cryosections, enabling for the first time high-resolution confocal fluorescence imaging of global SH activity in the tumor microenvironment. Tumor-associated cell types were identified by extensive immunohistochemistry on activity probe-labeled sections. Tissue-ABPP indicated heightened SH activity in glioma vs. normal brain and unveiled activity hotspots originating from tumor-associated neutrophils (TANs), rather than tumor-associated macrophages (TAMs). Thorough optimization and validation was provided by parallel gel-based ABPP combined with LC-MS/MS-based target verification.

**Conclusions:**

Our study advances the ABPP methodology to tissue sections, enabling high-resolution confocal fluorescence imaging of global SH activity in anatomically preserved complex native cellular environment. To achieve global portrait of SH activity throughout the section, a probe with broad reactivity towards the SH family members was employed. As ABPP requires no a priori knowledge of the identity of the target, we envisage no imaginable reason why the presently described approach would not work for sections regardless of species and tissue source.

## Background

The serine hydrolases (SHs) form a diverse family of enzymes with a predicted number of ∼240 in humans, falling into two subfamilies: the serine proteases (∼125 members) and the metabolic SHs (mSHs ∼115 members) [1, 2]. The mSHs include small-molecule hydrolases, such as lipases, esterases and amidases and utilize a conserved serine nucleophile to hydrolyze amide, ester, and thioester bonds in different types of substrates including metabolites, lipids and peptides. Importantly, SHs have emerged as therapeutic targets in diseases such as cancer, obesity, diabetes, and neurological diseases [3].

The advent of chemoproteomic techniques some 20 years ago, activity-based protein profiling (ABPP) in particular, allowed for the first time proteome-wide profiling of SH activity in cells and tissue homogenates [4]. The prototype activity probe for SHs features the active site-targeted warhead, typically a fluorophosphonate (FP), linked to a fluorescent reporter allowing in-gel imaging of SH activity in proteomes after SDS-PAGE separation. An advanced platform combining ABPP and multidimensional protein identification techniques (ABPP-MudPIT) was introduced to facilitate high-content functional proteomics discovery of potential new markers of human diseases [5].

In the comparative mode, ABPP enables comparison of SH activity pattern between different proteomes, e.g. aggressive vs. non-aggressive cancer cells [6]. Indeed, such studies have highlighted previously unrecognized importance of SH family members as metabolic nodes orchestrating the availability of lipids involved in oncogenic signaling.

In the competitive mode, ABPP has proven its power in the discovery and selectivity testing of novel inhibitors targeting individual SHs [7]. In this approach, the proteome is first treated with the inhibitor (commonly a serine-nucleophile targeting covalent inhibitor), binding of the inhibitor masks the active site, preventing subsequent labeling with the activity probe. EnPlex, an advanced high-throughput platform based on ABPP principles has been introduced as a feasible approach for SH superfamily-wide selectivity profiling that could be incorporated into the early stage of drug discovery [8].

ABPP can also serve a powerful platform for mass-spectrometry (MS) -based target identification from complex proteomes. In this case, the FP-warhead is linked to a biotin tag, enabling streptavidin enrichment and subsequent LC/MS-MS-based target identification.

Current ABPP approaches characterize global SH activity based on mobility in gel or MS-based target identification from homogenates and cannot reveal the identity of the cell-types responsible for an individual SH activity originating from complex proteomes, suggesting that the full potential of this technology may not have yet been harnessed. To date, quenched activity probe-based live cell fluorescence imaging has been described for a handful of proteases, such as cysteine cathepsins [9]. The quenched fluorescent probes were applied for labeling of active proteases in fresh-frozen cancer tissues [10], for macrophage detection in atherosclerotic plaques [11], and as a diagnostic tool to image skin tumor margins [12]. Recently, a small-molecule chemical toolbox was constructed for parallel imaging of human neutrophil serine proteases (NSPs). The toolbox comprised activity-probes with different fluorophores showing minimal wavelength overlap and highly selective natural and unnatural amino acid recognition sequences tailored for the four individual NSPs [13]. This elegant approach enabled for the first-time simultaneous imaging of the four NSPs in living neutrophils by fluorescence microscopy.

We reasoned that ABPP-based imaging of SH activity should be feasible at high resolution in complex native proteomes such as brain tissue while preserving the anatomical details of this delicate organ. Brain cryosections serve as fascinating premise to directly explore this issue as functional responses such as receptor-stimulated G protein activity and mSH-regulated endocannabinoid tone can be readily monitored in brain cryosections without compromising the anatomical integrity [14, 15].

Here, by using an activity probe with broad reactivity towards the SH family, we advance the ABPP methodology to glioma brain cryosections, enabling high-resolution confocal fluorescence imaging of global SH activity in glioma brain with well-preserved cyto-architecture. We unveil heightened SH activity in the tumor, as compared to healthy brain. Heterogeneous distribution pattern of SH activity within the tumor microenvironment was explored in detail by identification of the glioma-associated cell types using immunohistochemical markers. Cross-validation was provided by classical gel-based ABPP using homogenates of healthy brain and glioma, as well as by LC/MS-MS-based target identification. These studied revealed quite unexpectedly that SH activity hotspots in glioma originate from tumor-associated neutrophils (TANs), rather than tumor-associated macrophages (TAMs). We anticipate that tissue-ABPP should enable a wide range of applications for high-resolution confocal imaging of global SH activity in practically any type of tissue and species, opening new avenues for cellular and subcellular localization of SH activity in complex proteomes with preserved anatomy.

## Results

The principal motivation for this work was to extend the utility of ABPP towards applications enabling high-resolution imaging of global SH activity in cryosections of complex proteome, namely glioma brain, while preserving the delicate cyto-architecture of the tumor microenvironment. We chose to use a promiscuous activity probe for these studies in order to portrait the global SH activity profile in glioma brain sections. The tissue- and gel-based ABPP approaches that were utilized in this study are illustrated in Figure S1.

### The Glioma Model

Glioblastoma multiforme (GBM) is the most malignant and most frequent brain tumor accounting for more than 65% of all cases [16]. As the name implies, GBMs have a wide spectrum of histological morphologies ranging from small-cell type to very pleomorphic giant-cell forms with poor differentiation and gliosarcomas [16, 17]. We used the rat syngeneic BT4C gliosarcoma model [18] in which the glioma grows in a sarcomatous pattern and is typically composed of a pleomorphic population of tumor cells (Figure S2). We imaged tumors in vivo 22–24 days after implantation using MRI (Figure S3) and animals were sacrificed 5–11 days later. Brain tissue was excised from saline-perfused animals and used for cryosectioning (tissue-ABPP) or preparation of homogenates that were used in cross-validation experiments by gel-ABPP. Animals of both sexes were used.

### Optimization and Validation of ABPP Protocol for Brain Cryosections

As our ultimate goal was to achieve high-resolution imaging of SH activity in cryosections without significantly compromising anatomical integrity, we considered fixation necessary to preserve delicate cyto-architecture. On the other hand, fixation should be sufficiently mild in order to preserve enzymatic activity, a prerequisite for the probe to covalently label catalytically competent SHs. We found that in contrast to acetone or methanol, fixation with paraformaldehyde preserved tissue integrity and inhibitor sensitivity of TAMRA-FP labeling (Figure S4).

We chose 0.5 μM probe concentration as a compromise between signal intensity and cost-affordable probe amount (Figure S5). Concerning assay buffer, we found that for applications where maximal TAMRA-FP signal is the desired final readout, Tris or phosphate buffer (pH 7.4) without BSA supplementation is optimal. However, for applications where TAMRA-FP labeling step is followed by immunohistochemistry, BSA is included to block tissue prior to antibody addition (Figure S6).

### Distribution and Overall Characteristics of TAMRA-FP Signal in Glioma and Control Brain

**Fig. 1:**
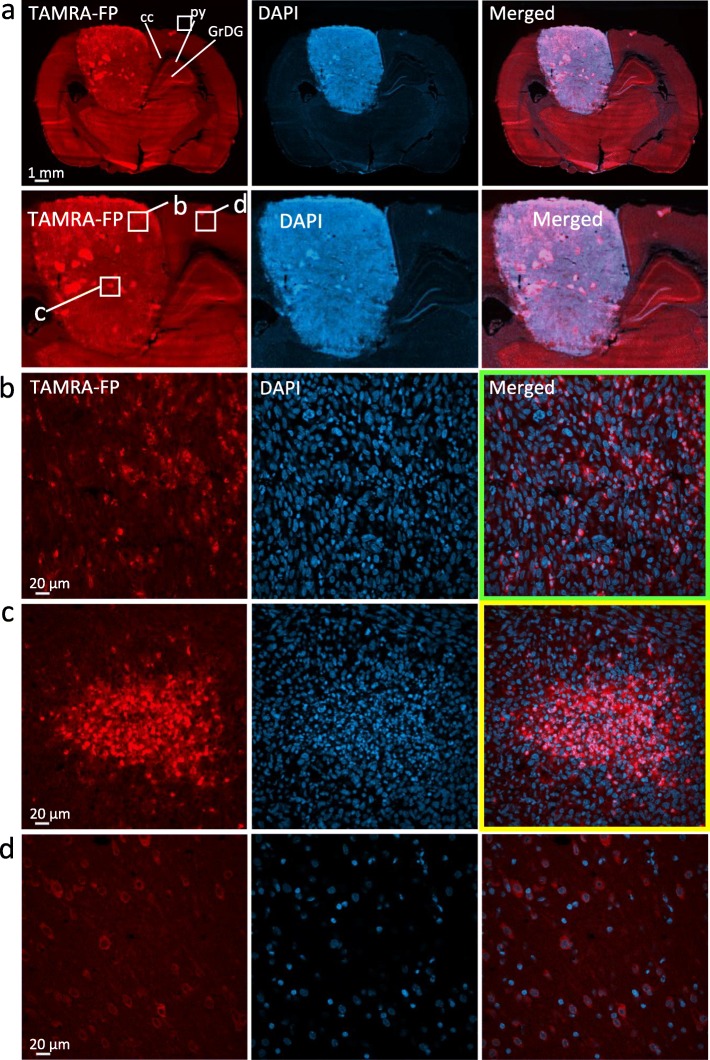
Distribution and overall characteristics of TAMRA-FP signal in glioma and control brain. Note relatively intense and heterogeneously distributed TAMRA-FP fluorescence over the glioma as compared to most regions of the healthy brain. Nuclear DAPI staining indicates dense cell population in glioma as compared to most regions of the healthy brain. Note relatively intense TAMRA-FP and DAPI signal also in cell-dense hippocampal pyramidal cell layer (py) and granular layer of dentate gyrus (GrDG) as well as relatively weak TAMRA-FP signal in white matter tracts of corpus callosum (cc) (**a**), suggesting that the method offers sufficient sensitivity to enable imaging of TAMRA-FP fluorescence not only in the tumor, but also in various regions of the healthy brain. Building on tissue-ABPP images of gliomas from different animals, a common pattern of TAMRA-FP labeling was established: TAMRA-FP hotspots (**b**), characterized by intense and widely-distributed non-nuclear TAMRA-FP signal over the glioma, originating from evenly distributed individual cells. We define the second pattern as TAMRA-FP hotspot clusters (**c**), characterized by intense non-nuclear TAMRA-FP signal originating from cell clusters. In healthy cortical region shown for comparison (**d**), TAMRA-FP signal is less intense and localizes mainly to cytosol and plasma membrane. The section illustrated here for TAMRA-FP and DAPI staining was further immunostained for the phagocyte marker CD11b/c and is presented again (Figure S18). 3D-animation of merged TAMRA-FP-DAPI fluorescence throughout the section thickness in TAMRA-FP hotspots (green lining) and TAMRA-FP hotspot clusters (yellow lining) is shown in Supplementary Video 1

As shown in Fig. 1, relatively intense and heterogeneously distributed TAMRA-FP labeling was evident over the glioma. In general, TAMRA-FP fluorescence was more intense over the glioma as compared to most regions of the healthy brain. Nuclear DAPI staining indicated dense cell population in glioma as compared to most regions of the healthy brain. This likely partly accounts for the heightened TAMRA-FP signal in glioma. Intense TAMRA-FP labeling was evident also in cell-dense structures of the healthy brain, such as hippocampal pyramidal cell layer and granular layer of dentate gyrus (Fig. 1a) and cerebellar Purkinje cell layer (Figure S6). On the other hand, white matter tracts (Fig. 1a) showed low TAMRA-FP labeling. Collectively these findings indicate that under the conditions employed, the method is sufficiently sensitive to allow imaging of TAMRA-FP labeling not only over the tumor, but also in various regions of the healthy brain.

Building on tissue-ABPP images of gliomas from different animals, a common pattern of TAMRA-FP labeling emerged and the following classification will be used to facilitate signal interpretation in forthcoming immunohistochemistry. A common TAMRA-FP labeling pattern with widest distribution over the glioma was characterized by non-nuclear intense fluorescence originating from evenly distributed individual cells (Fig. 1b, Video S1). In what follows, we define these cells “TAMRA-FP hotspots”. However, the most intense TAMRA-FP labeling pattern was non-nuclear and originated from cell clusters with variable size (Fig. 1c, Video S1). We define this labeling pattern “TAMRA-FP hotspot clusters”. In healthy cortex shown for comparison, TAMRA-FP fluorescence was less intense and mainly localized to cytosol and plasma membrane (Fig. 1d).

### Probe Labeling of Tumor and Healthy Brain SHs is Differentially Sensitive to SH Inhibitors

**Fig. 2:**
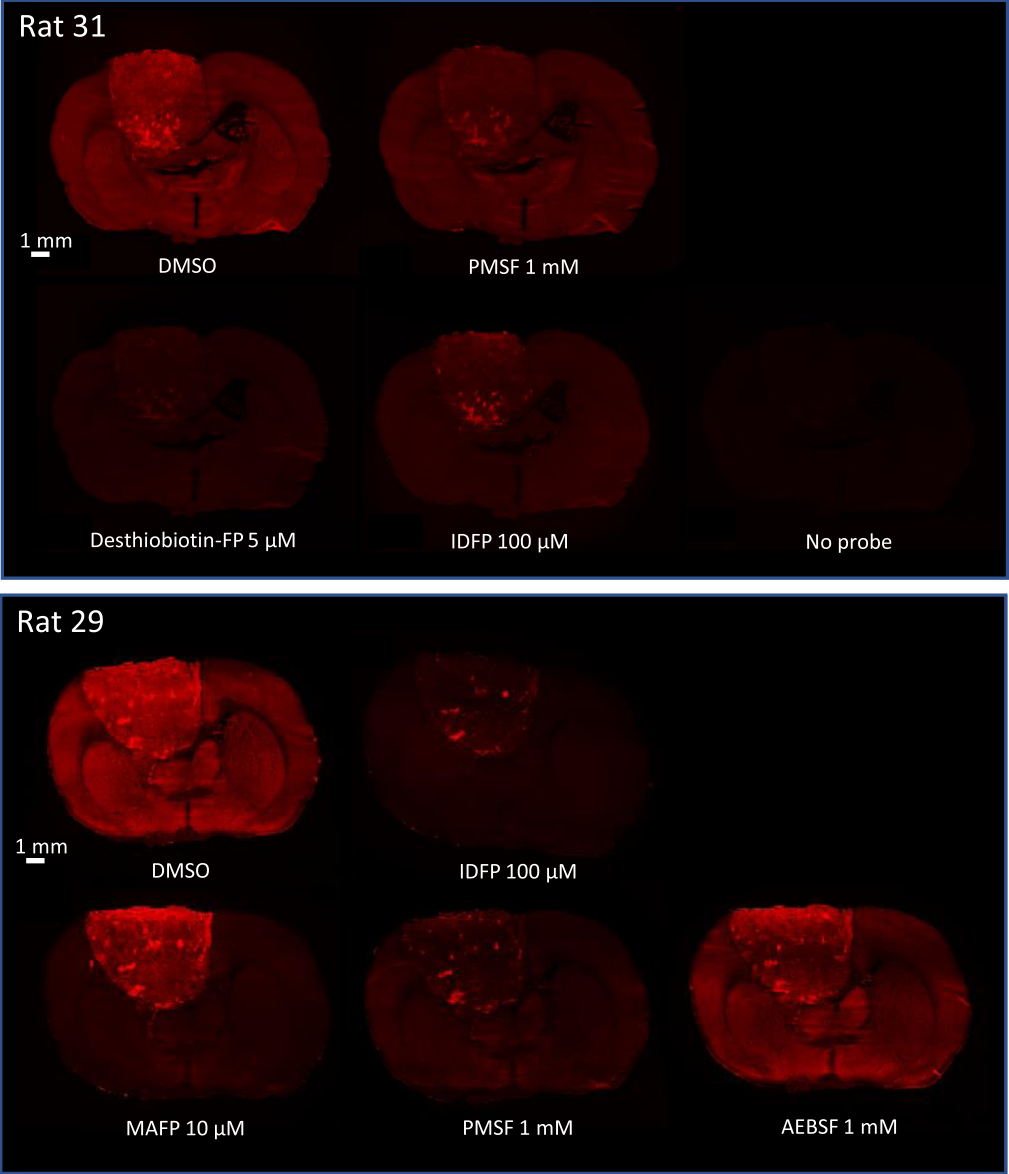
Competitive tissue-ABPP demonstrating that TAMRA-FP reports SH activity in glioma brain sections. Sections were pretreated for 1 h with the indicated concentrations of various serine-nucleophile targeting inhibitors and processed for TAMRA-FP labeling and confocal fluorescence imaging as detailed in Materials and Methods. Sections from two individual rats bearing the tumor were used and images from two independent experiments are shown as separate panels with DMSO control included in both experiments. Desthiobiotin-FP (5 μM) efficiently inhibits TAMRA-FP labeling throughout the brain section. However, a weak residual signal persists as spots over the glioma tissue. Note absence of fluorescence in the section processed without TAMRA-FP, indicating no detectable autofluorescence in the Cy3-window used for TAMRA-FP imaging. Note that PMSF (1 mM), in contrast to AEBSF (1 mM), and IDFP (100 μM) both efficiently inhibit TAMRA-FP labeling throughout the brain sections, yet leaving some residual activity over the tumor. Note in particular that MAFP (10 μM) efficiently inhibits probe labeling throughout the healthy brain regions but only modestly inhibited labeling of TAMRA-FP hotspots in the tumor. However, partial MAFP-sensitivity of TAMRA-FP signal was evident in tumors regions showing moderate probe fluorescence. The scale bars represent 1 mm. Images were adjusted for brightness and contrast

To assure that TAMRA-FP specifically reports SH activity under the conditions of tissue-ABPP, we used the competitive approach testing a panel of serine-nucleophile targeting broad-spectrum inhibitors, containing either FP [DeBi-FP (desthiobiotin-FP), MAFP (methyl arachidonyl fluorophosphonate), IDFP (isopropyl dodecylfluorophosphonate)] or sulfonylfluoride [AEBSF (aminoethylbenzenesulfonyl fluoride), PMSF (phenylmethylsulfonyl fluoride)] as the warhead. Inhibitors were purposely used at maximally effective concentrations to ensure comprehensive blockade of SH activity prior to TAMRA-FP labeling. As evident from Fig. 2, desthiobiotin-FP sharing the warhead-linker moiety with TAMRA-FP, effectively inhibited TAMRA-FP binding throughout the brain section, although weak residual labeling was evident as sparse spots over the glioma. Further, treatment with PMSF and IDFP efficiently prevented probe labeling in most brain regions, although some residual signal persisted over the tumor. Intriguingly, while the mSH-inhibitor MAFP efficiently blocked probe binding in most regions of the healthy brain, it was less effective in preventing TAMRA-FP labeling of the tumor, indicating that while MAFP-sensitive SH activity predominated in normal brain, SH activity originating from glioma was rather insensitive to this inhibitor. We considered this as a notable finding, as MAFP is known to act as a pan-SH inhibitor potently targeting the vast majority of mSHs [8]. When applied to rat brain cryosections in a comparable setting, our previous study indicated that MAFP (10 μM) was able to comprehensively block mSH-mediated hydrolysis of the major endocannabinoid 2-arachidonoylglycerol [14].

### Comparative Gel-Based ABPP of SH Activity in Homogenates of Glioma and Control Brain

**Fig. 3:**
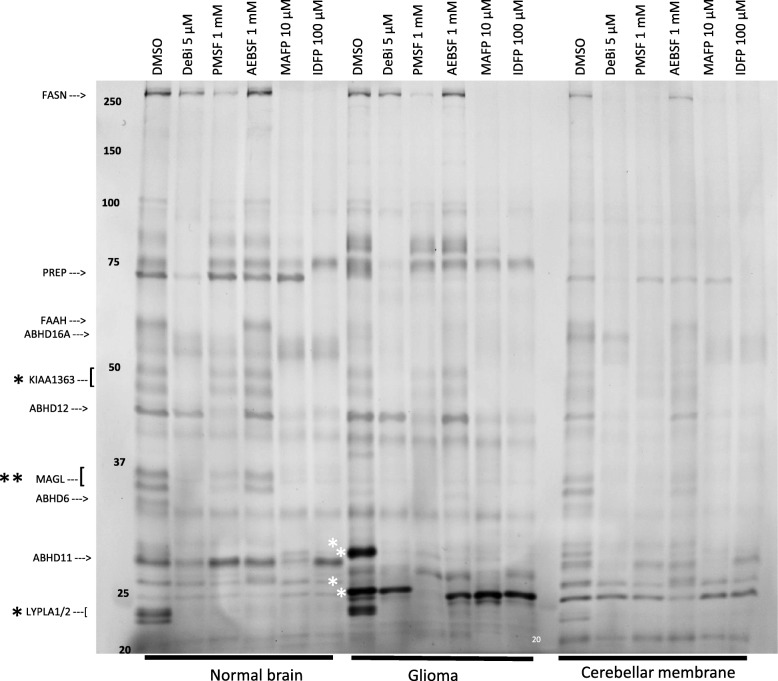
Comparative and competitive gel-based ABPP reveals distinct SH activity profiles of glioma and healthy brain. Rat cerebellar membranes were included as an additional control to facilitate SH band comparison and identification. Proteomes (1 mg/ml) were treated for 1 h with DMSO or the indicated concentrations of the SH inhibitors, after which TAMRA-FP labelling was conducted for 1 h as detailed in Materials and Methods. The reaction was quenched and 10 μg protein was loaded per lane and separated by SDS-PAGE. TAMRA-FP labeled bands appear as black after in-gel imaging. Position of molecular weight markers (in kDa) is indicated on the gel. Based on previous studies from this and other laboratories [14, 19–27] many healthy brain-resident SHs were identified, as indicated at left. Note comparable activities of KIAA1363 and LYPLA1/2 doublets (black asterisk) in control brain and glioma. Note also prominent activity of MAGL doublet (double black asterisk) in control brain as opposed to hardly detectable activity in glioma. Note that the glioma contains two prominent SH bands, migrating at ~ 25 and ~ 30 kDa (white asterisks). It is noteworthy that the respective SH bands show low-to-non-detectable activity in control brain. Inhibitor profiling reveals that the glioma 30 kDa band is sensitive to all tested inhibitor whereas the ~ 25 kDa band is fully sensitive to PMSF and less so to deshiobiotin-FP (DeBi), but resistant to other tested inhibitors, including MAFP. The gel is representative of two independent ABPP runs with similar outcome. ABHD1, ABHD6, ABHD11, ABHD12, ABHD16A, α/β-hydrolase domain-containing 1, 6, 11, 12 and 16A; FAAH, fatty acid amide hydrolase; FASN, fatty acid synthase; KIAA1363, also known as AADACL1 or NCEH1 (neutral cholesteryl ester hydrolase 1); LYPLA1/2, lysophospholipase A1/A2 (also known as acyl-protein thioesterases 1/2 or APT1/APT2); MAGL, monoacylglycerol lipase; PREP, prolyl oligopeptidase, also known as POP. Image was adjusted for brightness and contrast

We cross-validated the tissue-ABPP findings in gel-based ABPP using homogenates of glioma and control brain. In addition, we used rat cerebellar membranes to facilitate comparison, as previous gel-based ABPP has substantially relied on SH profiling in rodent brain membrane proteomes [14, 19–27]. Building on such studies from this and other laboratories [14, 19–27], we could identify many of the SH bands based on inhibitor sensitivity and mobility pattern in SDS-PAGE. These studies revealed that while many of the SH bands were common to the three proteomes (Fig. 3), brain-resident SHs, including monoacylglycerol lipase (MAGL ~ 35 kDa) and KIAA1363 (~ 50 kDa) showed variable activity in glioma. Both were proposed to play protumorigenic role in various cancers [6]. While comparable activity was evident for KIAA1363, MAGL showed low-to-non-detectable activity in glioma, suggesting that it might not play a major role in this glioma model. In addition, two distinct mSHs, namely lysophospholipases A1 and A2 (LYPLA1/2 ~ 25 kDa) showed prominent, yet comparable activity in control brain and glioma. LYPLA1/2 are depalmitoylases regulating membrane-association and oncogenic signaling of Ras, and are inhibited by palmostatin B ([28] and references therein).

Of note, the glioma possessed two prominent SH bands migrating at ~ 25 and ~ 30 kDa (Fig. 3). It is noteworthy that the respective SH bands showed low-to-non-detectable activity in control brain. We verified that the profile differences were present in homogenates from seven glioma rats (Figure S7). Inhibitor profiling revealed that the ~ 30 kDa band was sensitive to all tested inhibitors whereas the ~ 25 kDa band was sensitive to PMSF and deshiobiotin-FP, being resistant to other inhibitors, including MAFP. Collectively, the tissue and gel-based ABPP experiments suggest that MAFP-resistant TAMRA-FP hotspots in the tumor likely reflects activity of the dominant ~ 25 kDa band, while the bulk of MAFP-sensitive SH activity likely represent activity of the ~ 30 kDa prominent SH band. Noteworthy, in line with the outcome with glioma sections, PMSF efficiently prevented probe labeling of glioma homogenate in gel-ABPP (Fig. 3). The gel-ABPP findings not only cross-validate findings from tissue-ABPP but additionally indicate that the SH activity profile of glioma is distinct from that of normal brain and must therefore reflect activity originating from cell types not normally residing in brain.

### Tissue-ABPP Combined with Immunohistochemistry Enables Subcellular Localization of SH Activity within the Tumor Microenvironment

The glioma is a highly heterogeneous cancer and consists of several cell types, including tumor cells, endothelial cells from angiogenic vessels, neurons, scar-forming astroglia, as well as infiltrating monocytes/macrophages that are recruited from the periphery [29–31]. We approached this cellular complexity by immunohistochemistry using various cell-type markers that were applied on TAMRA-FP labeled sections.

To visualize tumor cells, sections were stained for Ki67, a nuclear marker of proliferating cell populations. Proliferating scattered cell populations were detected in glioma but as expected, were absent from healthy brain (Figure S8). Ki67-positive cells showed poor co-localization with TAMRA-FP hotspots or TAMRA-FP hotspot clusters.

GFAP-positive astrocytes appeared as a dense cell population encircling the tumor (Figure S9). Although scattered GFAP-positive cells were detected within the tumor, the bulk of tumor was devoid of astrocytes, in line with previous findings with this model [29]. The sparse presence of astrocytes is consistent with the gliosarcoma character of this model [18]. Although astrocytes showed moderate SH activity, TAMRA-FP hotspots or TAMRA-FP hotspot clusters showed poor co-localization with GFAP-positive cells. Throughout the healthy brain, cells with star-shaped morphology (i.e. astrocytes) were visible.

Tumor vasculature, visualized using the endothelial markers von Willebrand factor and CD34, revealed relatively low TAMRA-FP signal originating from endothelial cells (Figure S10). However, TAMRA-FP hotspot clusters resided often in the vicinity of the vessels. This glioma model shows leaky blood vessels with compromised blood-brain-barrier (BBB) function, especially at the tumor edge (Figure S1). Sections were stained for the redox-sensitive marker heme oxygenase-1 (HO-1) to visualize areas prone to redox stress (Figure S11). A heterogeneous pattern of HO-1 staining was evident in the tumor with no detectable signal in control brain. However, although HO-1 positive cells localized to regions of intense SH activity, neither TAMRA-FP hotspots nor TAMRA-FP hotspot clusters stained strongly for HO-1.

In the absence of truly selective microglial marker, we stained sections for Iba1 (Figure S12), being aware of that this marker also labels activated monocytes and macrophages. We observed heterogeneous presence of Iba1-positive cells throughout the tumor with intense staining in tumor margins, and noted partial overlap of Iba1 staining with TAMRA-FP hotspots. However, no Iba1-positive cells were evident in tumor regions of TAMRA-FP hotspot clusters. As expected, Iba1-positive cells with characteristic microglial morphology were detected in the healthy brain (Figure S12).

### SH Activity in Relation to Hyaluronan (HA) – CD44 Axis and Markers of Tumor Stiffness

Stromal HA accumulation is considered as a protumorigenic factor in several solid tumors [32, 33]. Extensive modeling of the extracellular matrix associated with biomechanical changes, often called tumor “stiffness”, is a hallmark of “cancerized” fibrotic stroma [32]. The HA receptor CD44 promotes cancer cell motility, tumor growth, angiogenesis as well as resistance to chemo- and radiotherapy, and is overexpressed in various tumors, including GBM [34]. We clarified whether TAMRA-FP hotspots localize to tumor regions undergoing matrix remodeling using the stiffness markers pMLC2 and tenascin C along with HA and CD44. HA was detected in the extracellular matrix throughout the brain with more intense staining in glioma, especially the glioma edges (Figure S13). HA was abundant in areas of TAMRA-FP hotspots. In contrast, HA was sparse in regions of TAMRA-FP hotspot clusters, whereas these clusters were surrounded by HA-enriched tissue. Like HA, CD44 was enriched in the glioma with low expression in normal brain (Figure S14). Like HA, CD44 was abundant in glioma regions of TAMRA-FP hotspots but was undetectable in regions of TAMRA-FP hotspot clusters, yet these clusters were surrounded by CD44-positive cells. pMLC2 showed intense staining over the glioma but was absent from healthy brain (Figure S15). Interestingly, the stiffness marker frequently co-localized with TAMRA-FP hotspots. In contrast, pMLC2-positive cells were rare in the central part of TAMRA-FP hotspot clusters whereas TAMRA-FP-positive cells at the edge of these clusters expressed pMLC2 and were also surrounded by pMLC2-positive cells. The outcome with tenascin C (Figure S16) was similar to what was observed with pMLC2.

### SH Activity in Relation to Cells of Myeloid and Lymphoid Origin

Bone-marrow-derived cell populations are abundant in tumors, including GBM [30, 31]. In human and rodent gliomas, tumor-associated macrophages (TAMs) are the major class of tumor-promoting immune cells and were also previously detected in this model [29]. We pursued to identify TAMs and other bone-marrow-derived cells in glioma. Interestingly, both TAMRA-FP hotspots and hot spot clusters expressed the hematopoietic marker CD45 (Figure S17) or the phagocyte marker CD11b/c (Figure S18). Although macrophages were abundant in glioma regions showing prominent SH activity, neither TAMRA-FP hotspots nor TAMRA-FP hotspot clusters originated from cells stained for the macrophage markers CD68 (Figure S19), CD163 (Figure S20) or CD169 (Figure S21). Lymphoid cells were present in glioma regions showing prominent SH activity and TAMRA-FP hotspots partially overlapped with CD4- and CD8-positive cells, whereas TAMRA-FP hotspot clusters sparsely expressed the T-cell markers (Figures S22 and S23).

### Tumor-Associated Mast Cells Partly Account for the High SH Activity

We found low expression of the mast cell marker FcεRIγ within TAMRA-FP hotspots whereas FcεRIγ-positive cells were enriched in TAMRA-FP hotspot clusters, showing marked co-localization with TAMRA-FP (Figure S24). Besides mast cells, FcεRIγ labels eosinophils, basophils and monocytes, so this marker alone cannot verify the presence of mast cells. Mast cell-specific serine proteases include tryptases and chymases. We stained the sections for chymase using CMA1 antibody. Faint expression of CMA1 was evident in glioma where CMA1-positive cells co-localized with TAMRA-FP hotspots, whereas CMA1-positive cells were scarce in TAMRA-FP hotspot clusters (Figure S25). Unfortunately, we found no additional antibodies that qualified for further verification of mast cell presence. Further, attempts to detect mast cells by enzymo-histochemical staining of serine protease activity using peptide substrates tailored for human tryptase and chymase [35] were unsuccessful (data not shown).

### Tumor-Associated Neutrophils (TANs) Largely Account for Tumor SH Activity Hotspots

**Fig. 4:**
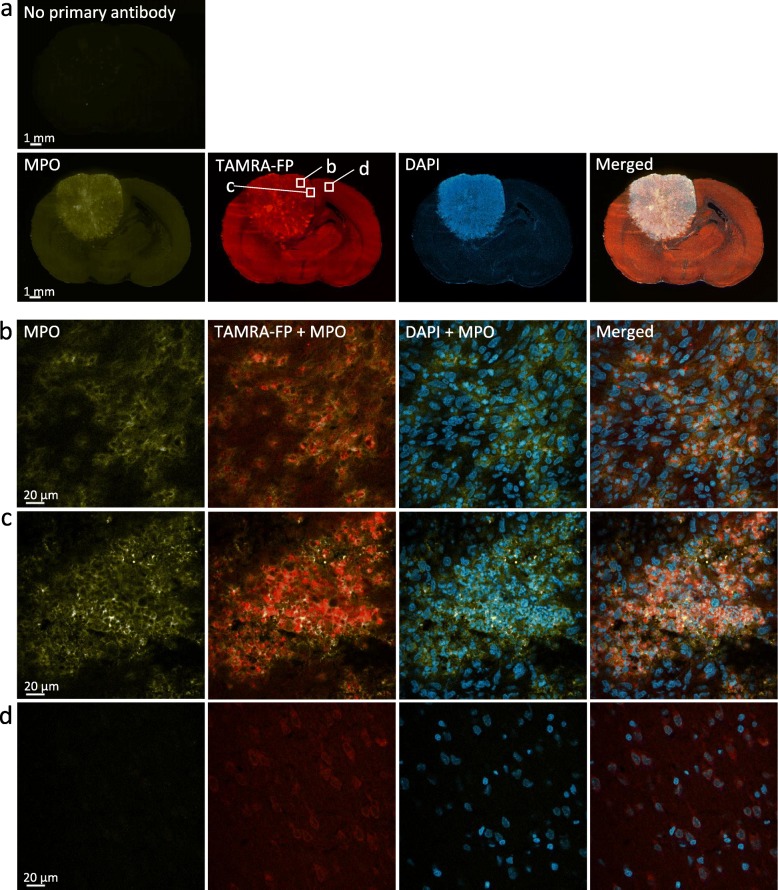
Confocal imaging of SH activity in relation to myeloperoxidase (MPO), a marker for neutrophils. Sections went through the tissue-ABPP protocol to label SHs (red) and were thereafter immunostained for MPO (yellow), followed by DAPI staining to visualize nuclei (blue). Panel **a** shows overall staining pattern throughout the coronal section plane. A control section undergoing identical staining protocol with no primary antibody is illustrated at top. Panel **b** shows staining pattern in glioma region characterized by intense SH activity originating from individual cells (TAMRA-FP hotspots). Panel **c** shows staining pattern in glioma region characterized by intense SH activity originating from cell clusters (TAMRA-FP hotspot clusters). Panel **d** shows staining pattern in healthy brain (cortex). Note detectable expression of MPO in the glioma (**a**). Note MPO-positive cells in the region of TAMRA-FP hotspots and marked co-localization of MPO with the TAMRA-FP signal (**b**). Note abundance of MPO-positive cells within TAMRA-FP hotspot clusters, as well as close match of MPO staining with TAMRA-FP hotspot clusters (**c**). MPO is not visible in control cortical region (**d**). Primary antibody rabbit anti-MPO (Abcam, cat# ab9535), dilution 1:25, secondary antibody Goat anti-rabbit IgG-Alexa Fluor 647 conjugate, dilution 1:100. Sections were from female rat 11. Scale bars: 1 mm in a, 20 μm in b-d. Images were adjusted for brightness and contrast

**Fig. 5:**
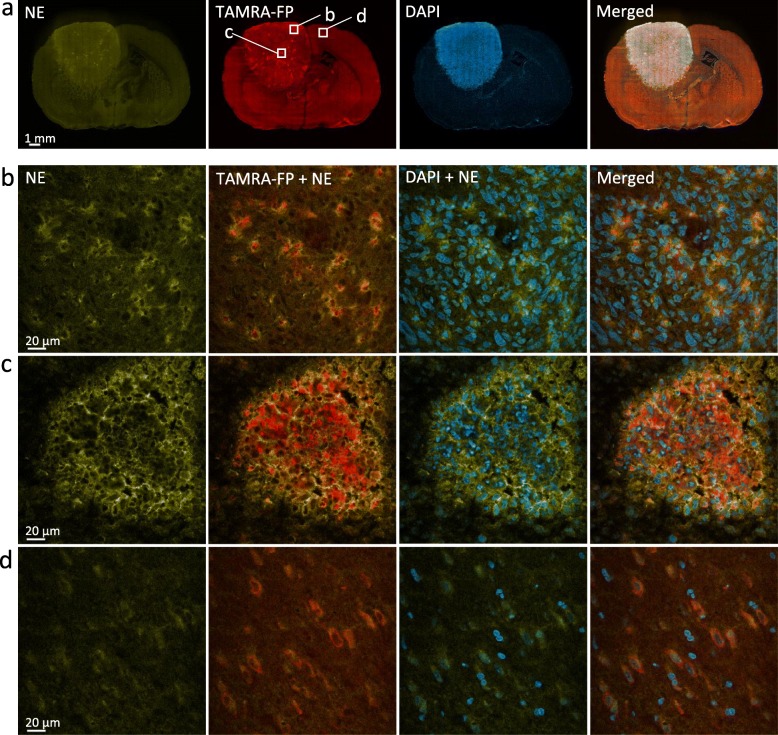
Confocal imaging of SH activity in relation to neutrophil elastase (NE). Sections went through the tissue-ABPP protocol to label SHs (red) and were thereafter immunostained for NE (yellow), followed by DAPI staining to visualize nuclei (blue). Panel **a** shows overall staining pattern throughout the coronal section plane. A control section undergoing identical staining protocol with no primary antibody was not available for this experiment. Panel **b** shows staining pattern in glioma region characterized by intense SH activity originating from individual cells (TAMRA-FP hotspots). Panel **c** shows staining pattern in glioma region characterized by intense SH activity originating from cell clusters (TAMRA-FP hotspot clusters). Panel **d** shows staining pattern in healthy brain (cortex). Note detectable expression of NE throughout the glioma (**a**). Note in particular NE-positive cells in the region of TAMRA-FP hotspots and close match of NE-positive cells with the TAMRA-FP signal (**b**). Note strong NE-immunostaining of the TAMRA-FP hotspot clusters and close match of NE staining within the TAMRA-FP hotspot cluster (**c**). NE is not visible in control cortical region (**d**). Primary antibody rabbit anti-NE (Abcam, cat# ab21595), dilution 1:500, secondary antibody Goat anti-rabbit IgG-Alexa Fluor 647 conjugate, dilution 1:100. Sections were from female rat 11. Scale bars: 1 mm in a, 20 μm in b-d. Images were adjusted for brightness and contrast

Neutrophils encompass four serine proteases (NSPs), namely neutrophil elastase (NE), cathepsin G (CTSG), proteinase 3 (PR3), and neutrophil proteinase 4 (NSP4, also known as PRSS57) [13, 36]. We stained glioma sections for the neutrophil marker myeloperoxidase (MPO) and found that MPO-positive cells localized to TAMRA-FP hotspots (Fig. 4). Further, MPO-positive cells were abundant in TAMRA-FP hotspot clusters (Fig. 4). The outcome was essentially the same in sections stained for the NSP elastase (Fig. 5).

**Fig. 6:**
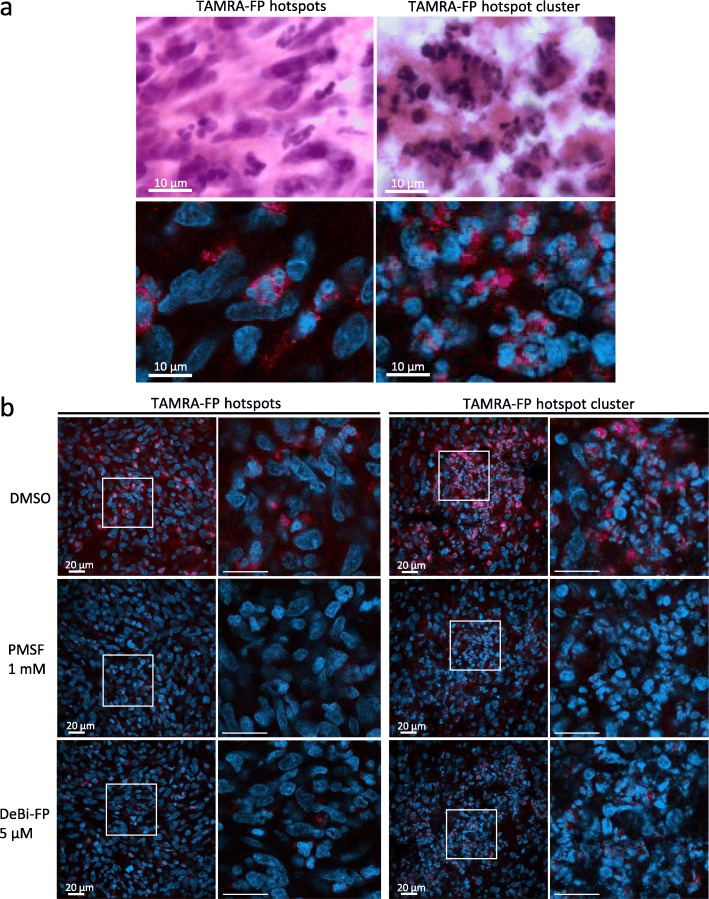
High-resolution imaging of TAMRA-FP hotspots and TAMRA-FP hotspot clusters and their inhibitor sensitivity in glioma. In a, the upper panel shows hematoxin-eosin (H&E) stained section. For images in the lower panel, sections went through the tissue-ABPP protocol to label SHs (red), followed by DAPI staining to visualize nuclei (blue). Note presence of multi-nucleated cells with TAN-like morphology in regions of TAMRA-FP hotspots and TAMRA-FP hotspot clusters. In **b**, sections were pretreated with DMSO or with the SH inhibitors PMSF (1 mM) or deshiobiotin-FP (DeBi-FP, 5 μM) for 1 h at RT, after which they went through the tissue-ABPP protocol to label SHs (red), followed by DAPI staining to visualize nuclei (blue). Note that throughout the examined regions, TAMRA-FP labeling is sensitive to the inhibitors. Scale bars 10 μm (**a**) and 20 μm (**b**). Images were adjusted for brightness and contrast

Noteworthy, high-resolution fluorescence imaging revealed that TAMRA-FP hotspots had the morphological characteristics of neutrophil-type multi-nucleated cells (Fig. 6). Importantly, TAMRA-FP labeling of these cells was sensitive to the SH inhibitors PMSF and DeBi-FP, indicating that TAMRA-FP fluorescence in the glioma hotspots and hotspot clusters indeed represents SH activity.

In sham-operated animals, TAMRA-FP hotspots with TAN-like morphology were rare at the site of injection (Figure S26), indicating that the surgical operation per se did not cause TAN accumulation. Collectively these findings indicated that TANs represent the principal cell-type accounting for TAMRA-FP hotspots and hotspot clusters.

### ABPP of Glioma Proteome Using Novel Activity Probes Designed for Human Serine Proteases

In further efforts to characterize glioma serine protease activity, we utilized newly introduced activity probes with preference for trypsin and elastase [9] using both gel- and tissue-based ABPP approaches. These probes bear a diphenylphosphonate (DPP) warhead, which targets serine proteases more specifically than the FP warhead which broadly targets the mSH family [37]. Besides glioma, we profiled tumor cells and bone-marrow-derived mononuclear cells (rBM). Interestingly, the elastase-preferring probe V-DPP detected 3–4 proteins (~ 25 kDa) in rBM and weakly two bands of similar size in glioma (Figure S27). Probe binding was sensitive to SH inhibitors, indicating that V-DPP specifically reported elastase-type serine protease activity. The trypsin-preferring probe PK-DPP gave no detectable signal in gel-ABPP (Figure S27). When tested in tissue-ABPP in place of TAMRA-FP, probe binding was hardly visible and neither probe labeled inhibitor-sensitive targets in glioma (Figure S28).

We evaluated also novel activity probes bearing the DPP warhead that were tailored to report individually activities of the four human NSPs [13]. Unfortunately, the NSP-probes did not recognize any TAMRA-FP sensitive bands in gel-ABPP of rat proteomes (Figure S29). When tested in tissue-ABPP in place of TAMRA-FP, the NSP-probes readily bound to sections, yet in a non-specific manner, as probe binding was not prevented by prior treatment with TAMRA-FP (Figure S29).

As a final step, we used gel-ABPP to assess inhibitor sensitivity of TAMRA-FP labeled proteins in neutrophils and rBMs (Figure S30). Only few prominent bands migrating at ~ 25–30 kDa were present in these proteomes and TAMRA-FP labeling of these bands was inhibited by PMSF, desthiobiotin-FP and AEBSF, but was largely resistant to MAFP and IDFP, consistent with the pharmacology of the ~ 25 kDa glioma band. Compound 22, designed to potently target human CTSG [38], was inactive in the rat proteomes whereas another CTSG inhibitor (CTSG-I) blocked TAMRA-FP labeling of the ~ 25 kDa band in both samples. In tissue-ABPP, the inhibitor did not prevent TAMRA-FP labeling of hotspots or hotspot clusters (Figure S30).

### LC-MS/MS Analysis of the Glioma SH Bands Migrating at ~ 25–30 kDa

**Fig. 7:**
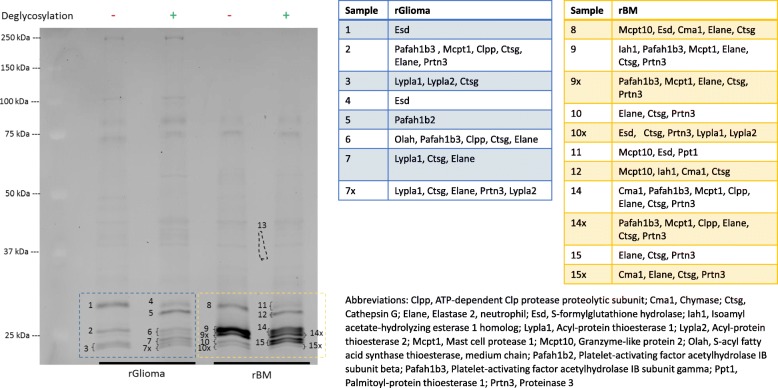
LC-MS/MS-based identification of the ~ 25–30 kDa SH bands in rat glioma and bone marrow-derived mononuclear cells. Gel-ABPP was conducted using rat glioma homogenates (rGlioma) or lysates of rat bone marrow-derived mononuclear cells (rBM) as detailed in Materials and Methods. To facilitate SDS-PAGE separation of proteins with similar size, the proteomes (4 mg/ml) were deglycosylated (**+**) or underwent control treatment (**−**) prior to TAMRA-FP labeling. For deglycosylation, samples were treated with Protein Deglycosylation Mix II (New England Biolabs, Cat#P6044S) for 1 h at RT as per kit instructions, after which TAMRA-FP labeling was conducted using routine gel-ABPP protocol. Following in-gel fluorescence imaging, gel-pieces encompassing the SH bands of interest (numbered 1-15x) were cut and subjected to LC-MS/MS analysis, as described in Materials and Methods. Gel-pieces marked with x were cut as one sample containing also the numbered gel-piece and split thereafter, yielding two separate samples that were subjected to LC-MS/MS. The SHs identified from the glioma samples (1-7x) are listed in the middle (blue-white table) and those identified from rBM (8-15x) at right (yellow-white table). Note presence of the NSPs CTSG (Ctsg), NE (elastase 2, Elane) and PR3 (Prtn3) in the ~ 25 kDa gel-pieces of both proteomes. Note also presence of platelet-activating factor acetylhydrolases 1b2 and 1b3 (PAFAH1b3 and PAFAH1b2) in the 25–30 kDa gel-pieces of both proteomes. The complete LC-MS/MS data listing all identified proteins and additionally data on BT4C glioma cells is available as Supplementary File 1

Gel-pieces encompassing the prominent SH bands in glioma, rBMs and tumor cells were cut after in-gel fluorescence scanning and subjected to LC-MS/MS-based target identification. To facilitate SDS-PAGE separation of proteins with similar size, proteomes were deglycosylated prior to TAMRA-FP labeling. The complete LC-MS/MS data with all identified proteins is available as Supplementary File 1. Interestingly, neutrophil serine proteases CTSG (Ctsg), NE (Elane) and PR3 (Prtn3) were among the SHs present in the ~ 25 kDa gel-pieces of glioma and bone marrow samples (Fig. 7), in strong support for findings from tissue-ABPP. Interestingly, the ~ 25–30 kDa gel-pieces encompassed also two MAFP-sensitive SHs [8], namely PAF-acetylhydrolases 1b2 and 1b3 (PAFAH1b3 and PAFAH1b2). Collectively, our experiments indicate that the MAFP-resistant TAMRA-FP signal in the tumor is principally due to neutrophil serine proteases migrating in the ~ 25 kDa band, while the bulk of MAFP-sensitive activity in glioma is likely due to PAFAH1b3 and PAFAH1b2.

## Discussion

Our study widens the applicability of the chemoproteomic technology to tissue sections, enabling for the first time high-resolution confocal fluorescence imaging of family-wide SH activity in anatomically preserved native cellular environment. Thorough optimization and validation was provided by parallel gel-based ABPP, extensive immunohistochemical mapping of TAMRA-FP signal origin, as well as LC-MS/MS-based target verification. Collectively all the evidence demonstrates convincingly that tissue-ABPP faithfully reports SH activity in cryosections at subcellular resolution. We emphasize that it is the TAMRA-FP labeling combined with immunohistochemistry and DAPI staining, each step utilizing different fluorophores with minimal wavelength overlap that makes tissue-ABPP a remarkably powerful new approach to portrait global SH activity in a complex proteome with preserved cyto-architecture.

The main focus of our studies was to achieve high-resolution imaging of global SH activity throughout the glioma microenvironment, necessitating systematic immunohistochemical mapping of origins of SH activity and its localization with cell-type and tissue stiffness markers. Together with inhibitor-sensitivity of TAMRA-FP labeling, these experiments provide the key validation for tissue-ABPP, ruling out the possibility that TAMRA-FP binding would be nonspecific. Besides illuminating the complexity of tumor microenvironment, our study unveiled heightened SH activity in glioma vs. normal brain and excluded major tumor-associated cells-types such as TAMs as a source of this activity. Unexpectedly, immunohistochemistry pinpointed TANs as the principal cell-type generating SH activity hotspots in glioma.

The activity probe that we used is commercially available and similar FP-probes have been previously used and extensively characterized for gel-based ABPP applications [2, 6, 7]. Furthermore, the coverage of mSH-family members recognized by the FP-probes is notable (> 80%) [37]. It could be argued that the use of broad-spectrum probe complicates interpretation of signals emerging from cryosections and therefore quenched probes with limited reactivity would be preferable. However, such probes are not yet widely available and as our principal aim was to provide global portrait of SH activity within the tumor microenvironment, the use of broad-spectrum probe was fully justified. In analogy, the vast majority of gel-based ABPP studies prefer to utilize broad-spectrum probes in order to achieve the global SH activity profile of the studied proteome. However, tissue-ABPP studies in future could utilize also newly-developed activity probes, tailored to target a particular subset of mSHs [39].

SHs are key regulators of metabolic pathways in diseases including cancer, neurodegenerative diseases, metabolic syndrome and atherosclerosis. Tissue-ABPP offers a new tool to explore the myriad roles of SHs likely play in physiology and disease. We anticipate that tissue-ABPP should find broad immediate applications. We applied tissue-ABPP also successfully to spleen sections, mainly to provide additional validation in native immune tissue for TAMRA-FP labeling in relation to some of the antibodies (Figures S31 and S32). Previously, a single study has presented low-resolution TAMRA-FP imaging of AEBSF-sensitive SH activity in prostate cancer specimen in supplementary figure [40]. Although preliminary, that work extends the applicability of tissue-ABPP to human proteomes. As ABPP requires no a priori knowledge of the identity of the target, we envisage no imaginable reason why tissue-ABPP would not work for sections regardless of species and tissue source.

Both cell morphology and immunohistochemical evidence implicated neutrophils as the principal cell type generating TAMRA-FP hotspots and hotspot clusters in glioma. We emphasize that the hotspots described here represent SH activity, as TAMRA-FP labeling was sensitive to the SH inhibitors PMSF and DeBi-FP (Fig. 6). Furthermore, LC-MS/MS verified the presence of NSPs in the gel-pieces encompassing the intense ~ 25 kDa TAMRA-FP bands, allowing to conclude that NSPs account for the bulk of this activity. In further support are findings showing that the elastase-preferring V-DPP-probe detected TAMRA-FP-sensitive ~ 25 kDa bands in glioma and that neutrophil and glioma proteomes shared similarly migrating intense SH bands with related pharmacology. Collectively these findings strongly suggest that NSPs rather than mSHs accounted for the TAMRA-FP hots spots and hotspot clusters.

The newly developed DPP-activity probes [13] and CTSG inhibitor Compound 22 [38] were designed to specifically target human NSPs. In our study, the compounds behaved as expected when tested using human proteomes (Figure S29 and data not shown). However, our cross-validation experiments clearly indicated that the Cy5-probes did not recognize mutual targets with TAMRA-FP in rat neutrophils or glioma. This was not totally unexpected, as both probe and inhibitor design exploited unique substrate recognition sequences around the catalytic sites which are not necessarily shared between human and rat NSPs. It is likely that the different warhead (DPP vs. FP) could also account for major differences in the reactivity and sensitivity of these probes to the inhibitors.

TANs are newcomers at the mainstage of cancer research [41]. In GBM, TANs seem to play mainly a tumorigenic role [42]. A recent study suggested involvement of TANs in GBM recurrence after radiotherapy by showing that neutrophils promoted cancer cells stemness [43]. In clinical samples, a positive correlation existed between neutrophils and patients diagnosed with recurrent tumor [43]. Future work could elucidate the role of TANs and NSPs in more detail.

Interestingly, the ~ 30 kDa SH was enriched in glioma and was not detected in healthy brain or neutrophils. Collectively our tissue and gel-based ABPP experiments suggested that the majority of the MAFP-sensitive SH activity in glioma likely reflects activity of the ~ 30 kDa prominent SH band. LC-MS/MS disclosed PAFAH1b3 and PAFAH1b2, among others, in the gel-pieces encompassing the ~ 25–30 kDa TAMRA-FP bands. Noteworthy, these mSHs are MAFP-sensitive [8] and were proposed to play protumorigenic role in various cancers [44–46]. Future work should elucidate the role of PAFAH1b3 and PAFAH1b2 in glioma.

The presently used rat gliosarcoma model has been useful to test novel chemotherapeutic targeting strategies, antitumor effects of gene therapy, anti-angiogenic agents alone or in combination with radiation and chemotherapy ([18] and references therein). We chose this model for convenience as it was previously used by researchers at our university [29] and the expertise to execute such studies was readily available.

Gel- and MS-based ABPP has shown that isolated mouse brain primary cells such as neurons, astrocytes and microglia each show characteristic SH activity profiles [47]. Tissue-ABPP offers a powerful tool for detailed investigation of this issue in situ with all normal cell types present. Such studies should be of wide interest to increase knowledge on the myriad roles that SHs likely play in the CNS. Based on studies from this and other laboratories [14, 19–27], gel-ABPP using brain membranes typically detects 20–30 SH bands, depending on the brain region studied (see e.g. Figure 3). The band intensities vary, reflecting activities of the individual SHs. Weak or hardly detectable bands set the practical limit for sensitivity of gel-ABPP. As the majority of mSHs are expressed in brain [1, 2], only a fraction of these can be detected using gel-ABPP. By analogy, the situation is likely similar concerning sensitivity of tissue-ABPP. However, confocal imaging settings can be adjusted to optimally capture TAMRA-FP signal also from brain regions showing low fluorescence. Our study indicates that under the conditions employed, tissue-ABPP should offer adequate sensitivity to enable imaging of SH activity not only in the tumor microenvironment but also in different regions of the healthy brain (Figs. 1 and S33). A straightforward starting point could be comparative tissue-ABPP of an individual SH, e.g. the principal endocannabinoid hydrolase MAGL using brain sections of wild-type and MAGL-deficient mice, as MAGL knockout does not lead to compensatory changes in the activity of other SHs, as evidenced by gel-based ABPP [25]. Competitive tissue-ABPP would offer a complementary approach for sections where MAGL is chemically inactivated with highly selective and ultra-potent inhibitors, such as JJKK-048 and KML29 [3, 14].

## Conclusions

Our study advances the ABPP approach to tissue sections, enabling high-resolution confocal fluorescence imaging of global SH activity in anatomically preserved complex native cellular environment. To achieve global portrait of SH activity throughout the tissue section, we used a probe with broad reactivity towards the SH family members. Thorough optimization and validation, complemented by gel-based ABPP, extensive immunohistochemical mapping enabling localization of SH hotspot activity, combined with LC-MS/MS-based target verification, collectively demonstrate that tissue-ABPP faithfully reports SH activity in cryosections. Tissue-ABPP is expected to provide sufficient degree of sensitivity, allowing profiling of not only heightened SH activity in the tumor, but also lower levels of SH activities in different regions of the healthy brain. As ABPP requires no a priori knowledge of the identity of the target, we envisage no imaginable reason why the presently described approach would not work for sections regardless of species and tissue source.

## Materials and Methods

The principal aim of this study was to achieve high-resolution imaging of global SH activity in glioma brain cryosections throughout the tumor microenvironment, necessitating systematic mapping of sources of SH activity by using cell-type specific immunohistochemical markers. To validate the tissue-ABPP approach, traditional gel-ABPP methodology combined with LC-MS/MS-based target identification was employed.

### Chemicals and Reagents

The following activity probes were used: TAMRA-FP (ActivX TAMRA-FP serine hydrolase probe, cat# 88318) and DeBi-FP (ActivX Desthiobiotin-FP Serine Hydrolase Probe, cat# 88317) were purchased from Thermo Scientific. The Cy5-labeled serine protease probes PK-DPP and V-DPP bearing a diphenylphosphonate (DPP) warhead [48] were used in selected experiments (Figures S27–28). Similarly, the Cy5-labeled NSP probes bearing a diphenylphosphonate (DPP) warhead [13] were used in selected experiments (Figure S29).

The following inhibitors were used: AEBSF (Sigma, cat#A8456), PMSF (Sigma, cat# P7626), IDFP (Cayman Chemical, cat# 10215), MAFP (Sigma, cat#M2939), CTSG-I (Calbiochem, cat# 219372). Compound 22 was obtained from Joakim Swedberg (at that time in the Institute for Molecular Bioscience, University of Queensland, Brisbane, Australia).

For immunohistochemistry, various antibodies from different sources were used. The hyaluronan binding probe (bHABR) was prepared in-house [49] (Figure S13). The following secondary antibodies were used: Goat anti-rabbit IgG-Alexa Fluor 647 conjugate (Thermo Scientific, cat# A-21245), Donkey anti-mouse IgG-Alexa Fluor 647 conjugate (Thermo Scientific, cat# A-31571),

Donkey anti-goat IgG-Alexa Fluor 647 conjugate (Thermo Scientific, cat# A-21447), and DyLight 488 Steptavidin (Vector Laboratories, cat# SA-5488). Primary antibody source, dilution used in immunostaining and choice of secondary antibodies are given in the captions of the images showing immunostained sections (Figures S8-S26 and S31-S32). Nuclei were stained with DAPI (4′,6-diamidino-2-phenylindole) (Sigma, cat# D8417). The BSA used was essentially fatty acid free (Sigma cat#A0281). All other chemicals were of highest purity available.

### Cell Culture

BT4C cells were grown in Dulbecco’s modified eagle’s medium DMEM (EuroClone, high glucose, with stable L-Glutamine, cat# ECM0103L) supplemented with 10% FBS (Euroclone, cat# ECS0180L) in a humidified cell culture incubator with 5% CO_2_ environment. The cells were maintained and sub-cultured two-three times a week at 1:6 splitting ratio. For preparation of the cell suspension for rat malignant glioma model, cells were trypsinized and collected in normal culture media (DMEM supplemented with 10% FBS) followed by a brief centrifugation at 1000 rpm, for 4 min at 4 °C, and re-suspended in fresh OPTI-MEM®I reduced serum medium (Gibco, cat# 31985–062). Cells were then counted and diluted in the same medium to 2 × 10^6^ cells/ml so that each 5 μl of cell suspension contained approximately 10,000 cells. For collecting cell pellets, cells were grown in several T-175 flasks until they become 95% confluent. On the day of collection, the cell culture media was aspirated from each flask, washed with cold 1xPBS, and cells were collected mechanically by using cell lifter/scraper in presence of 5 ml of cold 1x PBS. Cell fractions collected from different flasks were then combined into a 50 ml conical tube, washed with 1xPBS, centrifuged at low speed (300×g, for 10 min at 4 °C) to obtain opaque cell pellet. The pellet was stored at − 80 °C.

### Animals

#### Malignant Rat Glioma Model

In total 34 BDIX rats (18 males and 16 females, Envigo, Huntingdon, UK) weighing 127–267 g were used. The rats were anesthetized i.p. with ketamine (Ketalar®, 60 mg/kg) and medetomidine (Domitor®, 0.4 mg/kg) and placed in a stereotactic apparatus. After skin incision, a hole was drilled 1 mm posterior to the bregma and to the 2 mm right of sagittal suture. 10,000 BT4C tumor cells in 5 μl of OPTI-MEM®I reduced serum medium were injected with a Hamilton syringe within right corpus callosum (depth 2.5–3.0 mm). To avoid backflow, the injection was done slowly over 2–3 min. The needle was left in place for 2 min and then slowly removed. Skin incision was closed with stiches followed by s.c. injections of antisedative (atipamezole, Antisedan®, 1 mg/kg) and analgesic (carprofen, Rimadyl®, 5 mg/kg). Control rats were processed similarly, except that cells were omitted and only OPTI-MEM®I reduced serum medium was inoculated.

#### Tissue Harvesting from Glioma Animals

The rats were sacrificed 28–38 days after inoculation of the cells (the rats bearing tumors 28–31 days after inoculation). Animals were stunned with CO_2_ and then transcardially perfused with PBS. From majority of the rats, the whole brain was removed, dipped briefly in cold (− 80 °C) isopentane and stored on dry ice. From six rats in total, the tumor and corresponding brain piece from contralateral cerebral hemisphere were separated and were frozen on dry ice. For bone marrow collection, total of four bones (Tibia, Femur, Humerus, and Radius) were collected from each animal and stored in cold 1x PBS for further processing.

#### Control Rats Used for Harvesting Immune Cells and Tissue

For collecting rat spleens, cerebella, and fresh blood as the source of neutrophils, 10–12 week-old male Rcc:Han WIST rats (Laboratory Animal Centre, University of Eastern Finland) were used. Rats were decapitated and blood was collected with a funnel into a 50 ml conical tube soaked with EDTA (1.5–1.8 mg/ml of fresh blood). The blood was then processed within 2 h of collection. The cerebella were dissected, dipped briefly in cold (− 80 °C) isopentane and stored on dry ice. The spleens were collected, frozen on dry ice and then stored at − 80 °C.

#### Mice Used in Preliminary Tissue-ABPP Experiments

To test fixatives and TAMRA-FP concentrations during tissue-ABPP method optimization (Figures S4–S5), 4-week-old male JAXC57BL/6 J mice (Laboratory Animal Centre, University of Eastern Finland) were used. Mice were decapitated, the whole brain was removed, dipped briefly in cold (− 80 °C) isopentane and frozen on dry ice and then stored at − 80 °C.

### MRI

Magnetic resonance imaging (MRI) was used to verify tumor existence 12–14 and 22–24 days after inoculation of the cells. Anesthesia was induced with 5% isoflurane in a mixture of 70:30% N_2_O:O_2_ and was maintained at 1.5% isoflurane. MRI scanning was done with a 7 T Bruker PharmaScan system and ParaVision® 5.1 software (Bruker, Billerica, MA, USA). Turbo-RARE imaging sequence with the following parameters was used: TE 12.5 ms/50 ms, TR 4000 ms, RARE factor 8, FOV 2 cm, 256 × 256 matrix, slice thickness 1 mm with 15 slices. The images were processed with a home-build Matlab program Aedes (University of Eastern Finland).

### Cryosectioning

Coronal rat brain sections, horizontal mouse brain sections and rat spleen sections (20 μm thick) were cut at − 19 °C to − 21 °C using a Leica cryostat (Leica Biosystems, IL, USA). The sections (3 sections per slide) were thaw-mounted onto Superfrost®Plus slides (Menzel-Gläser, Germany), dried for 1–3 h at RT under a constant stream of air and stored thereafter at − 80 °C. Rat and mouse brain sections were cut according to Rat Brain Atlas (Paxinos & Watson 1998).

### Tissue-ABPP

Tissue-ABPP was used either for imaging SH activity alone or in combination with immunohistochemistry and nuclear staining, as detailed below.

#### Protocol for Imaging SH Activity

The assay was performed at RT (20–22 °C) unless otherwise stated. Generally, the fixing and all washing steps were performed in large volume (~ 200 ml) by dipping the slides into indicated buffer. Other steps of the protocol were performed by pipetting the indicated volume of buffer on the sections and by incubating the slides in a humidified chamber placed on a low-speed horizontal shaker.

The slides (3 tissue sections per slide) were thawed under a constant stream of air and thereafter each section was lined with a mini PAP pen (Fisher Scientific, cat# 10464573). The slides were fixed with 4% paraformaldehyde (PFA) supplemented with 0.01% glutaraldehyde in 0.1 M phosphate buffer (pH 7.4) for 10 min and rinsed thereafter 2 × 5 min in 1xPBS. The assay protocol consisted of preincubations 2 × 10 min in the assay buffer (250 μl/section) containing 50 mM Tris-HCl, pH 7.4; 1 mM EDTA; 100 mM NaCl; 5 mM MgCl_2_ and 0.1% (w/v) BSA, followed by 60 min incubation in the assay buffer (250 μl/section) in the presence of inhibitors or 1% (v/v) DMSO as a solvent. After washes 3 × 1 min in the assay buffer (250 μl/section), the slides were incubated for 60 min with TAMRA-FP in the assay buffer (95 μl/section, routinely 0.5 μM final concentration) and thereafter washed 3 × 10 min in 0.1 M phosphate buffer (pH 7.4). In some experiments, Cy-5 labelled probes were used instead of TAMRA-FP as detailed in Figures S28 and S29. In preliminary experiments, presented in Figures S1 and S4-S5, the slides were dried under constant stream of air and imaged thereafter using a Fuji gel scanner. In subsequent experiments, the slides were further stained with nuclear stain DAPI (2 μg/ml in 0.1 M phosphate buffer, 300 μl/section) for 15 min at 37 °C and washed 2 × 5 min with 0.1 M phosphate buffer (pH 7.4). Finally, the slides were embedded with Vectashield® Antifade mounting medium (cat# H-1000, Vector laboratories, CA, USA).

#### Tissue-ABPP Combined with Immunohistochemistry and Nuclear Staining

When immune markers were used, the preceding protocol for TAMRA-FP labeling is the same as described above. The TAMRA-FP labeled sections were washed 5 × 5 min with 1xPBS supplemented with 1% (w/v) BSA (210 μl/slide). The sections were incubated overnight with primary antibody (195 μl/slide) at 4 °C, washed 3 × 10 min with 0.1 M phosphate buffer (pH 7.4) followed by 60 min incubation with secondary antibody (195 μl/slide). Both primary and secondary antibodies were diluted in 1xPBS supplemented with 1% (w/v) BSA. Finally, the slides were washed 3 × 5 min with 0.1 M phosphate buffer (pH 7.4), stained with DAPI (2 μg/ml, 300 μl/slide) for 15 min at 37 °C, washed 2 × 5 min with 0.1 M phosphate buffer (pH 7.4) and embedded with Vectashield® Antifade mounting medium.

### Histology

To visualize tumor morphology, the sections were fist fixed with 4% PFA supplemented with 0.01% glutaraldehyde and then stained with Mayer’s hematoxylin and eosin. Imaging was performed with a Zeiss Axio Imager M2 microscope (Carl Zeiss Microimaging, Jena, Germany).

### Fluorescence Imaging

#### Fluorescence Scanning

For gel imaging and overview of tissue-ABPP slides, ChemiDoc™ MP imaging system (Bio-Rad, Hercules, CA, USA) was used. To visualize TAMRA-FP, Cy3 blot application (602/50, Green Epi) was used. For Cy5 probes or immunostained sections, Cy5 blot application (700/50, Red Epi) was used. In the early phase of this study (Figures S1, S4-S5), TAMRA-FP-labeled sections were imaged using Fuji gel scanner (λ_ex_ 552 nm/λ_em_ 575 nm).

#### Confocal Microscopy

The confocal microscope images were obtained with a Zeiss Axio Observer inverted microscope (10x, 40x (oil) or 63x (oil) -objectives) equipped with LSM800 confocal module (Carl Zeiss Microimaging GmbH, Jena, Germany). TAMRA-FP, DAPI, and secondary antibodies were imaged with 561 nm (λ_ex_ 543 nm/λ_em_ 567 nm), 405 nm (λ_ex_ 353 nm/λ_em_ 465 nm), and 640 nm (λ_ex_ 653 nm/λ_em_ 668 nm) lasers, respectively. Secondary antibody used with bHABR (Figure S13) was imaged with 488 nm (λ_ex_ 495 nm/λ_em_ 519 nm) laser. ZEN 2.3 (Blue) and ZEN 2.3 lite softwares (Carl Zeiss Microimaging GmbH) were utilized for image processing and image analysis.

### Rat Bone Marrow Cell Isolation

For bone marrow collection, total four bones (Tibia, Femur, Humerus, and Radius) were collected from each animal removing extra materials covering these bones, and stored in cold 1xPBS. The tip of each bone was cut with scissors, and flushed into a 50 ml conical tube with ice-cold 1xPBS using a 25G-needle, until no red color was visible. The sample was then homogenized using a pipette, filtered through Corning® cell strainer (CORNING, cat# 431750, 40 μm nylon), and centrifuged at 443×g for 4 min at 4 °C to pellet the cells, and suspended in 4 ml of 1xPBS. Four ml of ice cold ficoll-paque plus (GE Healthcare, 17–1440-02, density 1.077 ± 0.001 g/ml) was pipetted into a 10 ml transparent tube, and bone marrow cells (4 ml) were then overlaid on top of ficoll-paque plus carefully avoiding mixing of the two layers, then centrifuged at 443×g, 20 °C for 40 min without-brake in a swinging bucket centrifuge. The mononuclear cells were collected from the interface of PBS (top layer), and Ficoll-Red blood cells with granulocytes (two bottom layers). The cells were then washed by adding 3–6 volumes of 1xPBS followed by centrifugation at 1560×g for 4 min at 4 °C to remove extra Ficoll. The solid-white pellets were then stored at − 80 °C.

### Human and Rat Blood Neutrophil Isolation

For the isolation of neutrophils from human and rat blood, we used ficoll-paque plus (GE Healthcare, cat# 17–1440-02, Density 1.077 ± 0.001 g/ml) density gradient protocol adapted from [50, 51]. Human and rat samples were handled separately. Briefly, fresh blood samples were collected from 5 rats (~33 ml) and a human donor (~12 ml) in sodium EDTA (1.5–1.8 mg/ml of fresh blood). Five ml of blood was overlaid carefully on 5.0 ml of ficoll-paque plus in a 15 ml conical tube, centrifuged at 500×g for 35 min at RT with brake off. The granulocyte-rich pellet was collected by removing three upper layers (plasma, mononuclear cells, and remaining ficoll-paque plus), and processed further by preparing 1x HBSS-buffer (LONZA, BioWhittaker, cat# 04-315Q) suspension, 3% (w/v) dextran (Dextran T500, Pharmacia, cat# 17–0320-01, 450,000–550,000 M.W.) sedimentation, collecting granulocyte-enriched supernatant to a new tube; sample was centrifuged (300×g for 10 min at RT) and after removing the supernatant, remaining erythrocytes were lysed using hypotonic buffer, re-equilibrated to restore isotonicity, followed by centrifugation (300×g for 10 min at RT). Final washing and centrifugation (300×g for 10 min at RT) was done using HBSS buffer, resulting to clear white pellet. The pellet was stored at − 80 °C.

### Preparation of Proteomes for Gel-Based ABPP

Cell pellet was thawed in ice, and 100-500 μl of 1xPBS was added to each tube depending on the size of the pellet (approximately 1 mm-sized pellet in height, was resuspended in 100 μl of 1xPBS), mixed properly, spun, and went through freeze-thaw lysis procedure for at least five cycles (freezing at − 80 °C for 15 min or on dry ice for 5 min, thawing at 37 °C water bath for 1 min). Tissue samples were thawed in ice, weighted and required amount of 50 mM Tris-HCl, pH 7.4 + 150 mM NaCl buffer was added based on 1 ml volume per 1 g wet tissue-ratio, homogenized in glass-glass homogenizer in ice. The samples were collected into separate 2 ml Eppendorf tubes after washing with double volume of the Tris-buffer used initially. Finally, homogenized tissue samples and freeze-thaw-treated cell suspensions were sonicated on ice to obtain completely homogenized samples. Samples were then aliquoted, protein concentration was determined using BCA-200 Protein Assay Kit (PIERCE, 23226) as per company’s protocol, and stored at − 80 °C. Rat cerebellar membranes were prepared as previously described [52].

### Competitive Gel-Based ABPP

Competitive gel-ABPP was conducted using the Cy3-labeled probe TAMRA-FP or in selected experiments (Figures S28 and S29) various Cy5-labeled serine protease probes, following general outlines of our previous publication [53]. Briefly, 25 μl of proteomes (5–10 μg protein) diluted in PBS were preincubated with the indicated inhibitors (50-fold desired final concentration) or a vehicle (DMSO) for 1 h at RT, followed by addition of activity probe (in the case of TAMRA-FP, final concentration was 1 μM). The reaction was quenched by adding 2xSDS-loading buffer, followed by protein separation by SDS-PAGE (10%). For some lanes, Precision Plus Protein™ standards (BIO-RAD, cat# 161–0373) were included. In early phase of this study, TAMRA-FP fluorescence was imaged using Fujifilm FLA-3000 laser fluorescence scanner (Fujifilm, Tokyo, Japan) (Fluor. 532 nm; Filter: O580 nm). Thereafter, ChemiDoc™ MP imaging system (BIO-RAD, Hercules, California, USA) was used as follows: Cy3 blot application (602/50, Green Epi, Manual Exposure 10s–120s) was used, and Cy5 blot application (700/50, Red Epi, Manual Exposure 1 s) was used to image MW markers and all Cy5-labelled probes.

### Sample Preparation for LC-MS/MS

Gel casting and ABPP were done with special care in order to minimize potential contamination by foreign proteins. Gel electrophoresis and imaging was as described above. As TAMRA-FP labelling is visible only in imager under specific wavelength, the cutting of intended bands was done by keeping gel in a new, clear square BioAssay Dish (Corning, cat# 431111, 245 mmx 245 mm) put on a horizontally positioned computer screen where the imaged picture was opened and positioned accurately (as accurate as possible). The gel pieces was cut using scalpel no. 11 and collected into labelled 1.5 ml Eppendorf tube. The cut gel was then imaged again to check the accuracy of sample collection (Supplementary File 1_LC-MS-MS data). Collected samples were then wrapped with Parafilm®, stored in − 80 °C freezer, and shipped to Helsinki proteomics lab in dry ice contained box.

### LC-MS/MS Analysis

Protein bands were cut out of the polyacrylamide gel (Bio-Rad, USA) and “in-gel” digested cystein bonds were reduced with 0,045 M dithiothreitol (Sigma, cat# D0632) for 20 min at 37 °C and alkylated with 0,1 M iodoacetamide (Sigma-Aldrich, cat# 57670) at room temperature. Samples were digested by adding 0,75 μg trypsin (Sequencing Grade Modified Trypsin, V5111, Promega). After digestion peptides were purified with C18 microspin columns (Harvard Apparatus) according to manufactures protocol and re-dissolved in 30 μl.

Liquid chromatography coupled to tandem mass spectrometry (LC-MS/MS) analysis was carried out on an EASY-nLC1000 (Thermo Fisher Scientific, Germany) connected to a Velos Pro-Orbitrap Elite hybrid mass spectrometer (Thermo Fisher Scientific, Germany) with nano electrospray ion source (Thermo Fisher Scientific, Germany). The LC-MS/MS samples were separated using a two-column setup consisting of a 2 cm C18-A1 trap column (Thermo Fisher Scientific, Germany), followed by 10 cm C18-A2 analytical column (Thermo Fisher Scientific, Germany). The linear separation gradient consisted of 5% buffer B in 5 min, 35% buffer B in 60 min, 80% buffer B in 5 min and 100% buffer B in 10 min at a flow rate of 0,3 μl/min (buffer A: 0,1% TFA in 1% acetonitrile; buffer B: 0,1% TFA acid in 98% acetonitrile). 6 μl of sample was injected per LC-MS/MS run and analyzed. Full MS scan was acquired with a resolution of 60,000 at normal mass range in the orbitrap analyzer the method was set to fragment the 20 most intense precursor ions with CID (energy 35). Data was acquired using LTQ Tune software.

Acquired MS2 scans were searched against *Rattus norvegicus* protein data-base using the Sequest search algorithms in Thermo Proteome Discoverer. Allowed mass error for the precursor ions was 15 ppm. And for the fragment in 0,8 Da. A static modification parameter was set for carbamidomethyl + 57,021 Da (C) of cysteine residue. Methionine oxidation (+ 15,995 Da (M)) and TAMRA-FP (+ 659,312 Da (S, Y)) were set as dynamic modifications. Only full-tryptic peptides were allowed for scoring maximum of 1 missed cleavages were considered.

## Supplementary information

**Additional file 1 **: **Figure S1.** Activity-based protein profiling (ABPP) and the power of this approach to unveil SH activity in glioma. **Figure S2.** Characteristics of the rat BT4C gliosarcoma model. **Figure S3.** Coronal plane MRI images of glioma and control brains. **Figure S4.** Testing various fixation method for rodent brain sections. **Figure S5.** Effect of TAMRA-FP concentration on fluorescence signal in mouse brain sections. **Figure S6.** Effects of pH and buffer composition on TAMRA-FP signal and its inhibitor sensitivity. **Figure S7.** Comparative gel-based ABPP of seven animals confirming distinct SH activity profiles between glioma and control brain. **Figure S8.** Confocal imaging of SH activity in relation to proliferating tumor cells. **Figure S9.** Confocal imaging of SH activity in relation to astrocytes. **Figure S10.** Confocal imaging of SH activity in relation to blood vessels. **Figure S11.** Confocal imaging of SH activity in relation to heme oxygenase 1 (HO-1). **Figure S12.** Confocal imaging of SH activity in relation to microglial marker Iba1. **Figure S13.** Confocal imaging of SH activity in relation to hyaluronan (HA). **Figure S14.** Confocal imaging of SH activity in relation to HA receptor CD44. **Figure S15.** Confocal imaging of SH activity in relation to the stiffness marker pMLC2. **Figure S16.** Confocal imaging of SH activity in relation to the stiffness marker tenascin C. **Figure S17.** Confocal imaging of SH activity in relation to CD45, a marker for nucleated hematopoietic cells. **Figure S18.** Confocal imaging of SH activity in relation to CD11b/c, a marker for phagocytes. **Figure S19.** Confocal imaging of SH activity in relation to CD68, a marker for monocytes and macrophages. **Figure S20.** Confocal imaging of SH activity in relation to CD163, a marker for monocytes and macrophages. **Figure S21.** Confocal imaging of SH activity in relation to CD169, a marker for macrophages. **Figure S22.** Confocal imaging of SH activity in relation to T cell marker CD4. **Figure S23.** Confocal imaging of SH activity in relation to T cell marker CD8. **Figure S24.** Confocal imaging of SH activity in relation to FcεRIγ, a marker for mast cells, eosinophils, basophils and monocytes. **Figure S25.** Confocal imaging of SH activity in relation to chymase (CMA1), a marker for mast cells. **Figure S26.** TAMRA-FP signal at the site of injection in sham-operated animals. **Figure S27.** Gel-ABPP of rat glioma proteomes using Cy5-labeled serine protease activity probes PK-DPP and V-DPP. **Figure S28.** Tissue-ABPP of glioma sections using Cy5-labeled activity probes PK-DPP and V-DPP. **Figure S29.** ABPP of rat neutrophil and glioma samples using Cy5-labeled neutrophil serine protease (NSP) probes in combination with TAMRA-FP. **Figure S30.** Inhibitor profiles of human cathepsin G (hCTSG) and the prominent 25–30 kDa SH bands in rat bone-marrow-derived mononuclear cells and neutrophils. **Figure S3**1**.** High-resolution imaging of TAMRA-FP hotspots and their inhibitor sensitivity in rat spleen. **Figure S32.** Confocal imaging of SH activity in rat spleen sections in relation to selected immunomarkers. **Figure S33.** Tissue-ABPP offers sufficient sensitivity to enable imaging of TAMRA-FP fluorescence in regions of the healthy brain.

**Additional file 2 Video S1.** 3D-animation of merged TAMRA-FP-DAPI fluorescence throughout the section thickness in TAMRA-FP hotspots and TAMRA-FP hotspot clusters (related to Fig. 1).

**Additional file 3.** Complete LC-MS/MS data of all proteins identified from the ABPP gel-pieces.

## Data Availability

The datasets supporting the conclusions of this article are included within the article and its additional files. Correspondence and request for materials should be addressed to J.T.L. or J.R.S., to P. K and M.D. (NSP probes) or to L.E.M. (elastase/tryptase probes).

## Abbreviations

ABHD: α/β-hydrolase domain-containing
ABPP: Activity-based protein profiling
ABPP-MudPIT: A platform combining ABPP and multidimensional protein identification techniques
AEBSF: Aminoethylbenzenesulfonyl fluoride
APT1/APT2: Acyl-protein thioesterase 1/2
BBB: Blood-brain-barrier
BSA: Bovine serum albumin
CD: Cluster of differentiation
CTSG: Cathepsin G
CTSG-I: CTSG inhibitor
DAPI: 4′,6-diamidine-2′-phenylindole
DeBi-FP: Desthiobiotin-FP
DPP: Diphenylphosphonate
EnPlex: A high-throughput platform based on ABPP
FAAH: Fatty acid amide hydrolase
FASN: Fatty acid synthase
FP: Fluorophosphonate
GBM: Glioblastoma multiforme
GFAP: Glial fibrillary acidic protein
HA: Hyaluronan
HO-1: Heme oxygenase-1
IDFP: Isopropyl dodecylfluorophosphonate
LC/MS-MS: Liquid chromatography coupled to tandem mass spectrometry
LYPLA1/2: Lysophospholipase A1/A2
MAFP: Methyl arachidonyl fluorophosphonate
MAGL: Monoacylglycerol lipase
MPO: Myeloperoxidase
mSH: Metabolic serine hydrolase
NCEH1: Neutral cholesteryl ester hydrolase 1
NE: Neutrophil elastase (NE)
NSP: Neutrophil serine protease
NSP4: Neutrophil proteinase 4
PAFAH1b2/1b3: PAF-acetylhydrolase 1b2/1b3
pMLC2: Phosphorylated myosin light chain 2
PMSF: Phenylmethylsulfonyl fluoride
PR3: Proteinase 3
PREP/POP: Prolyl oligopeptidase
rBM: Rat bone-marrow-derived mononuclear cells
SDS-PAGE: Sodium dodecyl sulfate polyacrylamide gel electrophoresis
SH: Serine hydrolase
TAMRA-FP: Serine hydrolase probe
TAMs: Tumor-associated macrophages
TANs: Tumor-associated neutrophils
