# Supplementary material for: High-Resolution Confocal Fluorescence Imaging of Serine Hydrolase Activity in Cryosections – Application to Glioma Brain Unveils Activity Hotspots Originating from Tumor-Associated Neutrophils
Source: Biol Proced Online. 2020 Mar 15;22:6. doi: 10.1186/s12575-020-00118-4 (PMC7073015; doi:10.1186/s12575-020-00118-4)
Supplement: Supplementary file 1 — Additional file 1 : Figure S1. Activity-based protein profiling (ABPP) and the power of this approach to unveil SH activity in glioma. Figure S2. Characteristics of the rat BT4C gliosarcoma model. Figure S3. Coronal plane MRI images of glioma and control brains. Figure S4. Testing various fixation method for rodent brain sections. Figure S5. Effect of TAMRA-FP concentration on fluorescence signal in mouse brain sections. Figure S6. Effects of pH and buffer composition on TAMRA-FP signal and its inhibitor sensitivity. Figure S7. Comparative gel-based ABPP of seven animals confirming distinct SH activity profiles between glioma and control brain. Figure S8. Confocal imaging of SH activity in relation to proliferating tumor cells. Figure S9. Confocal imaging of SH activity in relation to astrocytes. Figure S10. Confocal imaging of SH activity in relation to blood vessels. Figure S11. Confocal imaging of SH activity in relation to heme oxygenase 1 (HO-1). Figure S12. Confocal imaging of SH activity in relation to microglial marker Iba1. Figure S13. Confocal imaging of SH activity in relation to hyaluronan (HA). Figure S14. Confocal imaging of SH activity in relation to HA receptor CD44. Figure S15. Confocal imaging of SH activity in relation to the stiffness marker pMLC2. Figure S16. Confocal imaging of SH activity in relation to the stiffness marker tenascin C. Figure S17. Confocal imaging of SH activity in relation to CD45, a marker for nucleated hematopoietic cells. Figure S18. Confocal imaging of SH activity in relation to CD11b/c, a marker for phagocytes. Figure S19. Confocal imaging of SH activity in relation to CD68, a marker for monocytes and macrophages. Figure S20. Confocal imaging of SH activity in relation to CD163, a marker for monocytes and macrophages. Figure S21. Confocal imaging of SH activity in relation to CD169, a marker for macrophages. Figure S22. Confocal imaging of SH activity in relation to T cell marker CD4. Figure S23. Confocal imaging of SH a [file 12575_2020_118_MOESM1_ESM.pdf]

Supplemental information 31.1.2020

High-resolution confocal fluorescence imaging of serine hydrolase activity in cryosections – Application to glioma brain unveils activity hotspots originating from tumor-associated neutrophils

Niina Aaltonen, Prosanta K. Singha, Hermina Jakupović, Thomas Wirth, Haritha Samaranayake, Sanna Pasonen-Seppänen, Kirsi Rilla, Markku Varjosalo, Laura E. Edgington-Mitchell, Paulina Kasperkiewicz, Marcin Drag, Sara Kälvälä, Eemeli Moisio, Juha R. Savinainen & Jarmo T. Laitinen

Inventory

Supplementary Figures

Figure S1. Activity-based protein profiling (ABPP) and the power of this approach to unveil SH activity in glioma.

Figure S2. Characteristics of the rat BT4C gliosarcoma model.

Figure S3. Coronal plane MRI images of glioma and control brains.

Figure S4. Testing various fixation method for rodent brain sections.

Figure S5. Effect of TAMRA-FP concentration on fluorescence signal in mouse brain sections.

Figure S6. Effects of pH and buffer composition on TAMRA-FP signal and its inhibitor sensitivity.

Figure S7. Comparative gel-based ABPP of seven animals confirming distinct SH activity profiles between glioma and control brain.

Figure S8. Confocal imaging of SH activity in relation to proliferating tumor cells.

Figure S9. Confocal imaging of SH activity in relation to astrocytes.

Figure S10. Confocal imaging of SH activity in relation to blood vessels.

Figure S11. Confocal imaging of SH activity in relation to heme oxygenase 1 (HO-1).

Figure S12. Confocal imaging of SH activity in relation to microglial marker Iba1.

Figure S13. Confocal imaging of SH activity in relation to hyaluronan (HA).

Figure S14. Confocal imaging of SH activity in relation to HA receptor CD44.

Figure S15. Confocal imaging of SH activity in relation to the stiffness marker pMLC2.

Figure S16. Confocal imaging of SH activity in relation to the stiffness marker tenascin C.

Figure S17. Confocal imaging of SH activity in relation to CD45, a marker for nucleated hematopoietic cells.

Figure S18. Confocal imaging of SH activity in relation to CD11b/c, a marker for phagocytes.

Figure S19. Confocal imaging of SH activity in relation to CD68, a marker for monocytes and macrophages.

Figure S20. Confocal imaging of SH activity in relation to CD163, a marker for monocytes and macrophages.

Figure S21. Confocal imaging of SH activity in relation to CD169, a marker for macrophages.

Figure S22. Confocal imaging of SH activity in relation to T cell marker CD4.

Figure S23. Confocal imaging of SH activity in relation to T cell marker CD8.

Figure S24. Confocal imaging of SH activity in relation to FcεRIγ, a marker for mast cells, eosinophils, basophils and monocytes.

Figure S25. Confocal imaging of SH activity in relation to chymase (CMA1), a marker for mast cells.

Figure S26. TAMRA-FP signal at the site of injection in sham-operated animals.

Figure S27. Gel-ABPP of rat glioma proteomes using Cy5-labeled serine protease activity probes PK-DPP and V-DPP.

Figure S28. Tissue-ABPP of glioma sections using Cy5-labeled activity probes PK-DPP and V-DPP.

Figure S29. ABPP of rat neutrophil and glioma samples using Cy5-labeled neutrophil serine protease (NSP) probes in combination with TAMRA-FP.

Figure S30. Inhibitor profiles of human cathepsin G (hCTSG) and the prominent 25-30 kDa SH bands in rat bone-marrow-derived mononuclear cells and neutrophils.

Figure S31. High-resolution imaging of TAMRA-FP hotspots and their inhibitor sensitivity in rat spleen.

Figure S32. Confocal imaging of SH activity in rat spleen sections in relation to selected immunomarkers.

Figure S33. Tissue-ABPP offers sufficient sensitivity to enable imaging of TAMRA-FP fluorescence in regions of the healthy brain.

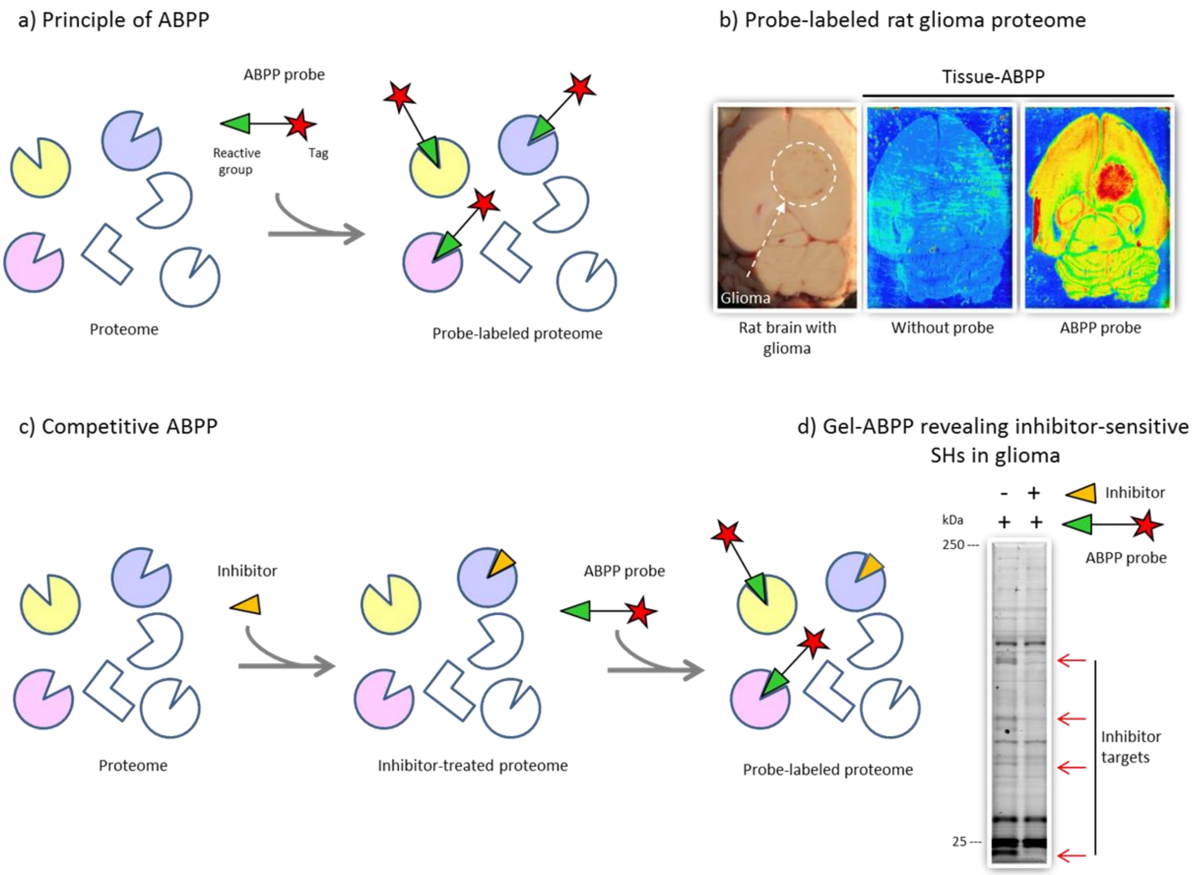

Figure S1. Activity-based protein profiling (ABPP) and the power of this approach to unveil SH activity in glioma. a) An active site-targeted covalent probe comprises a reactive fluorophosphonate (FP) group (warhead) that is coupled via a linker to a reporter tag (e.g. fluorescent dye or biotin) to covalently label, detect, enrich and identify active SHs (colored objects in this scheme). **b)** Tissue-ABPP unveils heightened SH activity in rat glioma. As compared to healthy brain tissue, the tumor lights up in red due to high SH activity, evident in the brain section treated with the ABPP probe TAMRA-FP (panel at right). Due to leaky blood vessels, the glioma borders appear as a brownish halo, as illustrated for the brain used in cryosectioning (panel at left). In competitive ABPP (**c**), the proteome is first treated with an inhibitor that covalently binds to the catalytic serine, inactivating the target enzyme. Thereafter, the proteome is reacted with the activity probe. Probe labeling of the inhibitor-targeted enzyme is prevented (a single target is illustrated here). **d)** Competitive gel-based ABPP revealing molecular size, relative abundance and inhibitor-sensitivity of the glioma-enriched SHs. The proteins are resolved using SDS-PAGE and visualized after in-gel fluorescence scanning. Treatment with a broadly-acting lipase inhibitor prevents probe labeling of several SH bands (red arrows). Images in panels A and C are modified from [1].

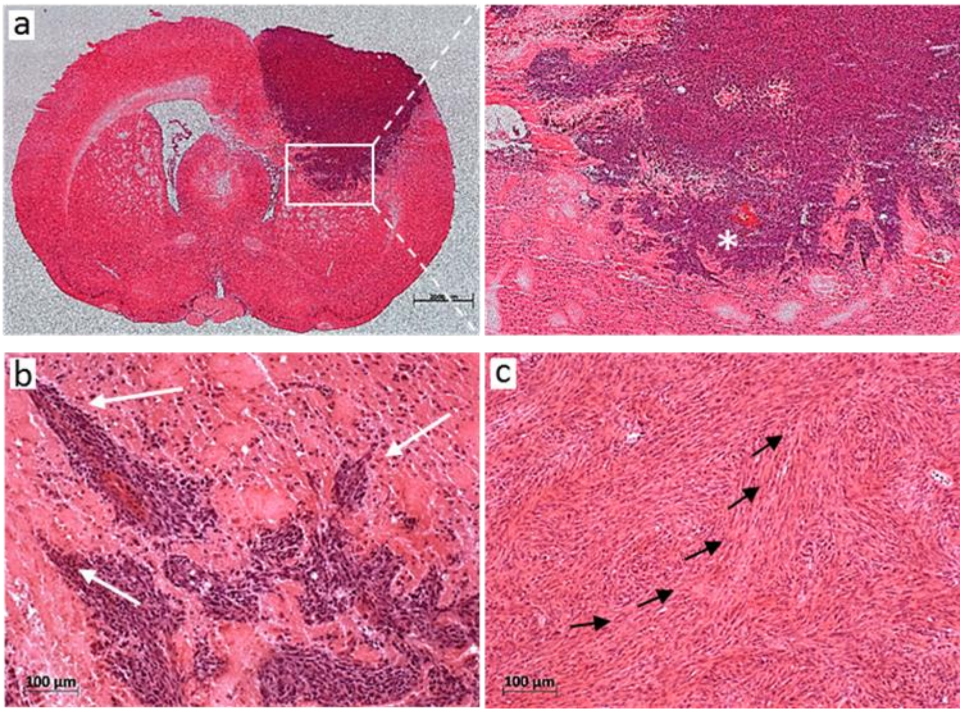

Figure S2. Characteristics of the rat BT4C gliosarcoma model. Tumors were generated as detailed in Materials and Methods and their presence verified by MRI imaging in vivo 22-24 days after implantation of tumor cells (Figure S3). Animals were sacrificed 5-11 days after MRI imaging, i.e. 4-5 weeks after implantation of tumor cells. 20 µm thick coronal brain cryosections were fixed with 4% PFA supplemented with 0.01% glutaraldehyde and stained with hematoxylin-eosin (H&E). The tumor grows expansively, invading the surrounding normal brain. Microhemorrhages are seen in tumor periphery (white asterisk) (**a**). White arrows point leading edges of migrating tumor cells (**b**). In tumor bed, H&E staining reveals sarcomatous pattern of glioma growth (black arrows) with elongated, spindle shaped cells (**c**). Scale bars 2 mm (a) and 100 µm (b and c).

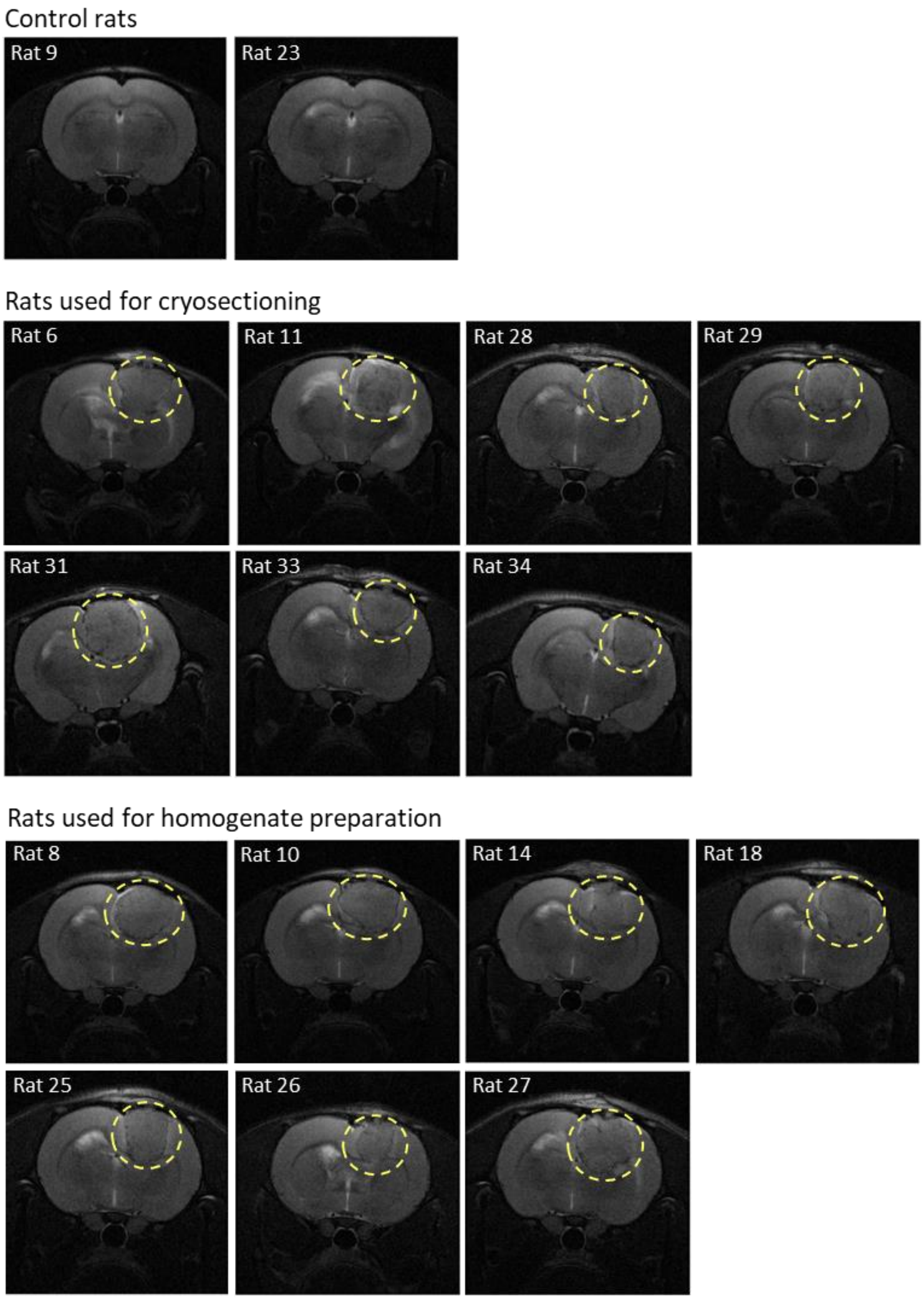

Figure S3. Coronal plane MRI images of glioma and control brains. Brains were used for cryosectioning (tissue-ABPP) or for preparation of homogenates (gel-based ABPP for validation experiments). Rats 9 and 23 were injected with the tumor cell medium only and served as controls to provide sections from the site of tumor implantation. Gliomas are visible inside the dashed circles. Images were adjusted for brightness and contrast.

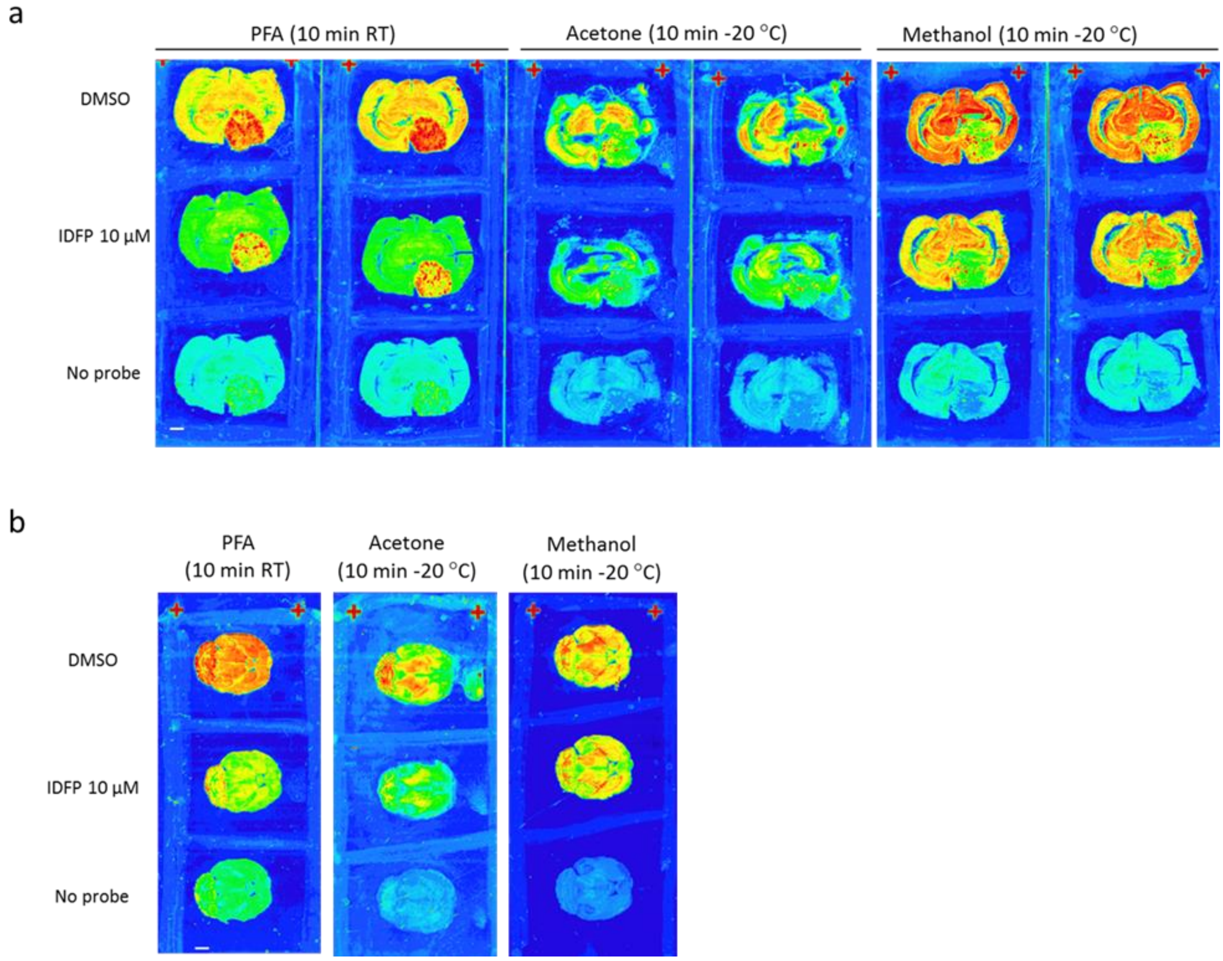

Figure S4. Testing various fixation method for rodent brain sections. Coronal sections (20 µm thick) of glioma brain (**a**) or horizontal sections of mouse brain (**b**) were fixed using either 4% paraformaldehyde supplemented with 0.01 % glutaraldehyde (PFA, 10 min at RT), or acetone (10 min at -20 °C) or methanol (10 min at -20 °C), washed for 2x5 min in 0.1 M PBS and processed thereafter for tissue-ABPP as detailed in Materials and Methods. Sections were pretreated with DMSO (top and bottom rows) or with the broad-spectrum SH inhibitor isopropyl dodecylfluorophosphonate (IDFP, 10 µM) for 1h at RT, after which they were incubated for 1h at RT with (top and middle rows) or without (bottom row) TAMRA-FP (0.5 µM). After washes, sections were imaged using Fuji gel scanner (λ_{ex} 552 nm/λ_{em} 575 nm) for visual assessment of tissue integrity and inhibitor sensitivity of TAMRA-FP labeling. Fluorescence intensity is shown in arbitrary color-scale where red and blue denotes high and low fluorescence, respectively. The images in panels **a** and **b** originate from separate experiments and fluorescence colors are not comparable between the experiments. Note that while PFA fixation preserves both tissue integrity and inhibitor sensitivity of TAMRA-FP labeling, both parameters are compromised in sections fixed with acetone or methanol. Images in **a** are from duplicate slides with three consecutive coronal sections in each, cut from glioma rat 2 or from slides with three consecutive horizontal sections of normal mouse brain in each (**b**). Scale bar (white) 2 mm.

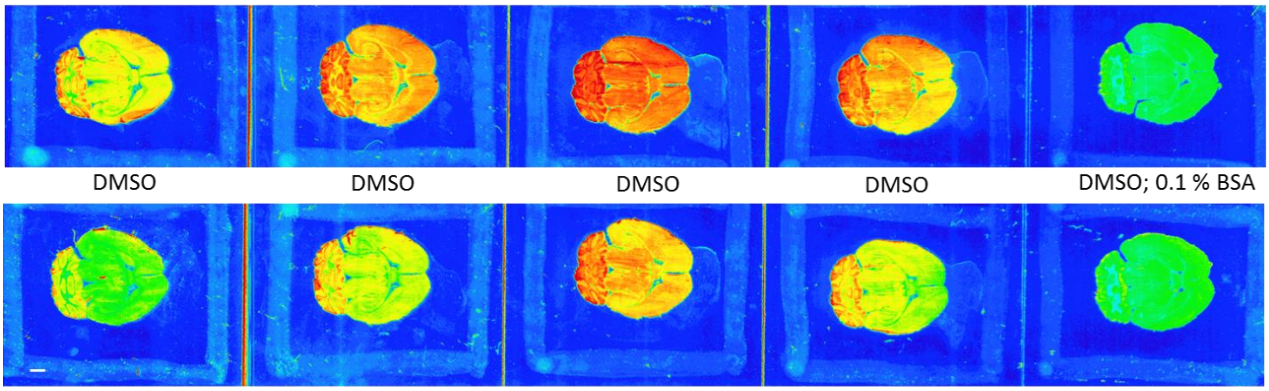

Figure S5. Effect of TAMRA-FP concentration on fluorescence signal in mouse brain sections. Horizontal mouse brain sections (20 µm thick) were fixed using PFA (10 min at RT), washed for 2x5 min in 0.1 M PBS and processed for tissue-ABPP as detailed in Materials and Methods. Sections were pretreated with DMSO or with 10 µM IDFP for 1h at RT, after which they were incubated for 1h at RT with or without increasing concentrations of TAMRA-FP (0.2, 0.5 or 2 µM). After washes, sections were imaged using Fuji gel scanner (λ_{ex} 552 nm/λ_{em} 575 nm) for visual assessment of fluorescence intensity and inhibitor sensitivity of probe labeling. Intensity of the fluorescence signal is shown as arbitrary colors where red and blue denotes high and low fluorescence, respectively. Note increasing signal intensity with increasing probe concentration. Note also IDFP-sensitivity of TAMRA-FP labeling regardless of probe concentration. Note also that omission of BSA (0.1 % w/v) only marginally affects signal intensity or inhibitor sensitivity. Scale bar (white) 2 mm.

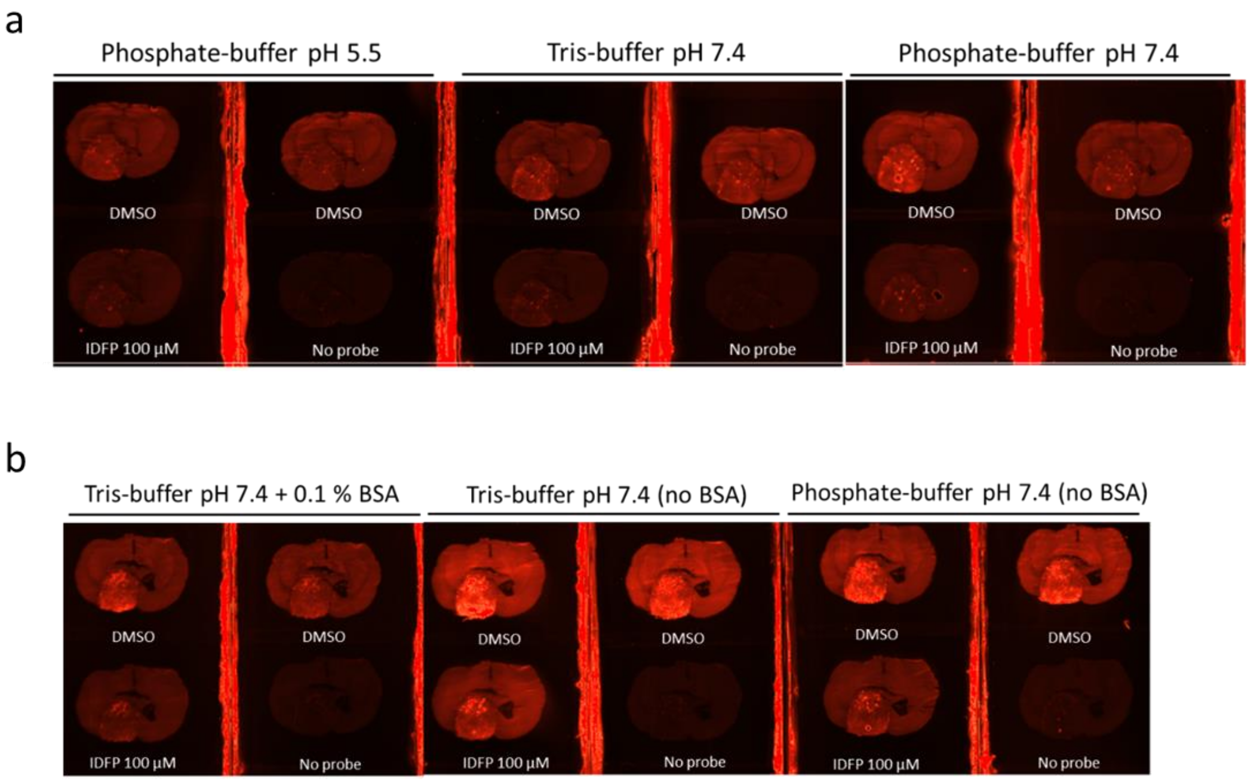

Figure S6. Effects of pH and buffer composition on TAMRA-FP signal and its inhibitor sensitivity. Coronal sections (20 µm thick) of rat glioma brain were fixed using PFA (10 min at RT), washed for 2x5 min in 0.1 M PBS and processed for tissue-ABPP, as detailed in Materials and Methods. Sections were pretreated with DMSO or with 100 µM IDFP for 1h at RT, followed by labeling step for 1h at RT with or without TAMRA-FP (0.5 µM) using the indicated buffer systems with varying pH (**a**) or buffer composition at pH 7.4 (**b**). After washes, sections were imaged using BioRad gel scanner (Cy3-window) for visual assessment of fluorescence intensity and inhibitor sensitivity of probe labeling. TAMRA-FP fluorescence is in red. **a.** Phosphate- vs. Tris-buffer (Tris-$MgCl_2$-NaCl-EDTA, pH 7.4), both containing 0.1 % BSA (w/v). Note that TAMRA-FP fluorescence is more intense at pH 7.4, being somewhat weaker in Tris-buffer at the same pH, and clearly blunted in the phosphate-buffer at pH 5.5. **b.** TAMRA-FP signal is comparable in Tris- vs. phosphate-buffer, inclusion of 0.1 % BSA clearly blunts TAMRA-FP fluorescence. Note that inhibitor sensitivity of TAMRA-FP labeling is retained in all conditions. For applications where imaging of maximal TAMRA-FP labeling is the desired endpoint readout, optimal signal is obtained in Tris- or phosphate buffer in the absence of BSA. For applications where TAMRA-FP labeling is followed by immunohistochemistry applied on the same section, inclusion of BSA is justified as a preceding facilitatory step to block tissue before application of primary antibody. Images were adjusted for brightness and contrast.

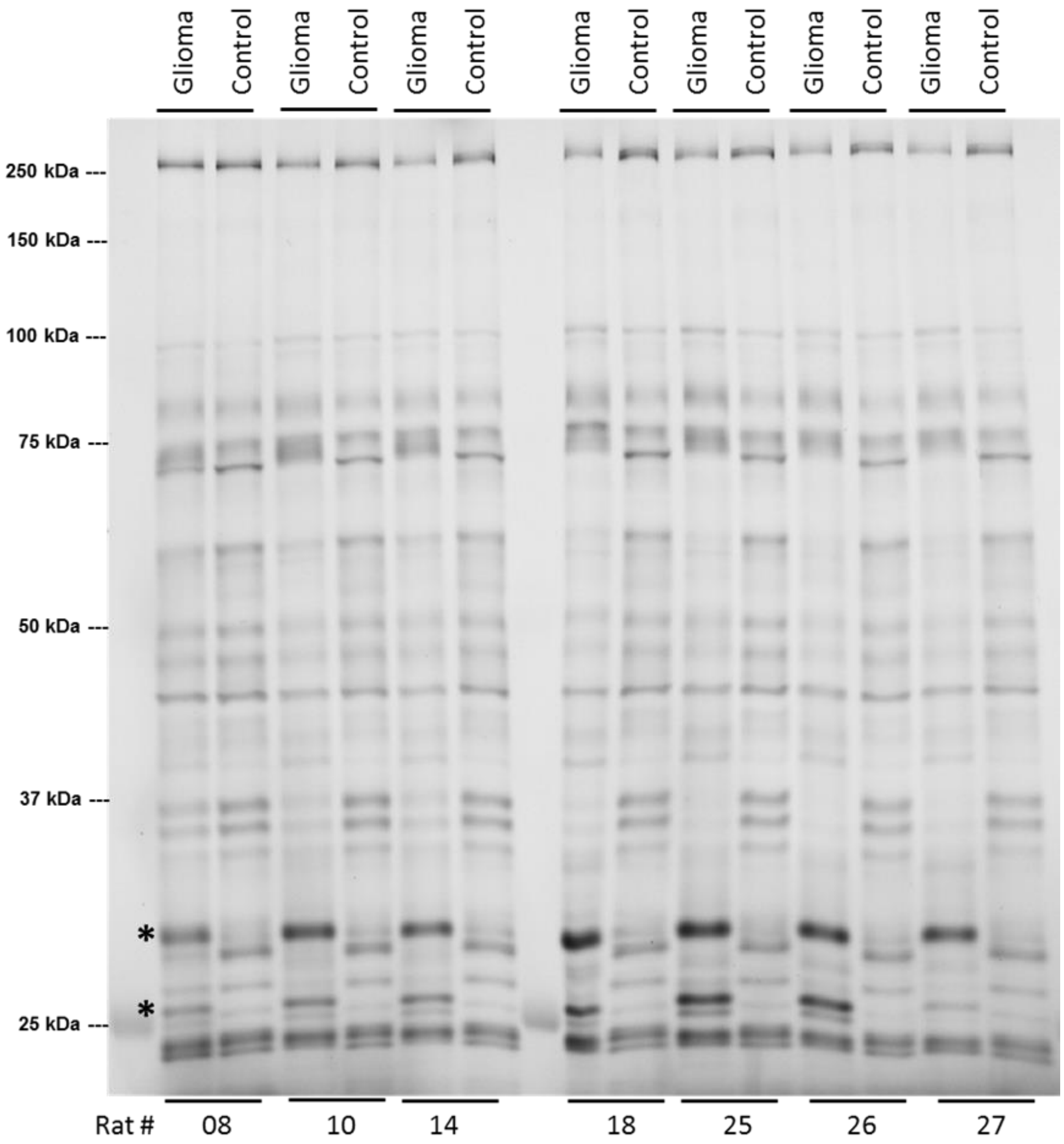

Figure S7. Comparative gel-based ABPP of seven animals confirming distinct SH activity profiles between glioma and control brain. Proteomes (1 mg/ml) were treated with TAMRA-FP (1 µM) for 1h at RT as detailed in Materials and Methods. The reaction was quenched and 12.5 µg protein was loaded per lane, followed by protein separation using SDS-PAGE. Shown are TAMRA-FP labeled bands (dark) after in-gel imaging. Position of molecular weight markers is indicated at left. Note similar activity profile between the seven gliomas; glioma-enriched SH bands (black asterisks) migrate at ~25 and ~30 kDa. Note also similar activity profiles between the seven control brains and particularly that the SH activity profiles of glioma and control brain are distinct. Image was adjusted for brightness and contrast.

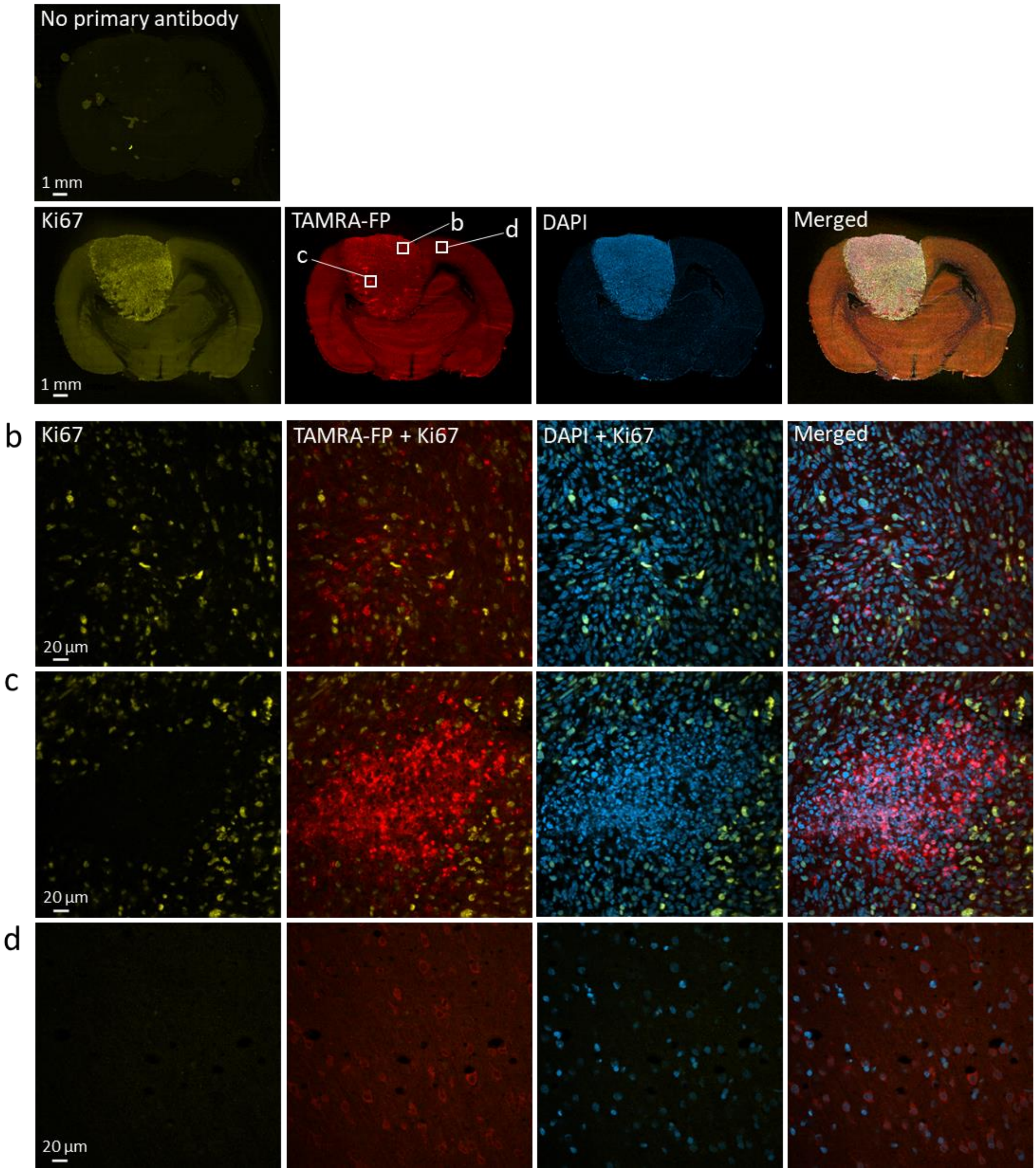

Figure S8. Confocal imaging of SH activity in relation to proliferating tumor cells. Sections went through the tissue-ABPP protocol to label SHs (red) and were thereafter immunostained for Ki67 to visualize proliferating cells (yellow), followed by DAPI staining to visualize nuclei (blue). Panel **a** shows overall staining pattern throughout the coronal section plane. A control section undergoing identical staining protocol with no primary antibody is illustrated at top. Panel **b** shows staining pattern in glioma region characterized by intense SH activity originating from individual cells (TAMRA-FP hotspots). Panel **c** shows staining pattern in glioma region characterized by intense SH activity originating from cell clusters (TAMRA-FP hotspot clusters). Panel **d** shows staining pattern in healthy brain (cortex). Note scattered population of proliferating cells with large nuclei and with distinct morphology in region of TAMRA-FP hotspots (**b**). Note proliferating cells around the region with TAMRA-FP hotspot clusters and lack of co-localization with TAMPA-FP signal (**c**). Note absence of Ki67 immunostaining from the healthy brain (**d**). Magnification 10x in **a**, 40x in **b-d**. Primary antibody rabbit anti-Ki67 (Abcam, cat# ab15580), dilution 1:500, secondary antibody goat anti-rabbit IgG-Alexa Fluor 647 conjugate, dilution 1:100. Sections were from male rat 29. Scale bars: 1 mm in a, 20 µm in b-d. Images were adjusted for brightness and contrast.

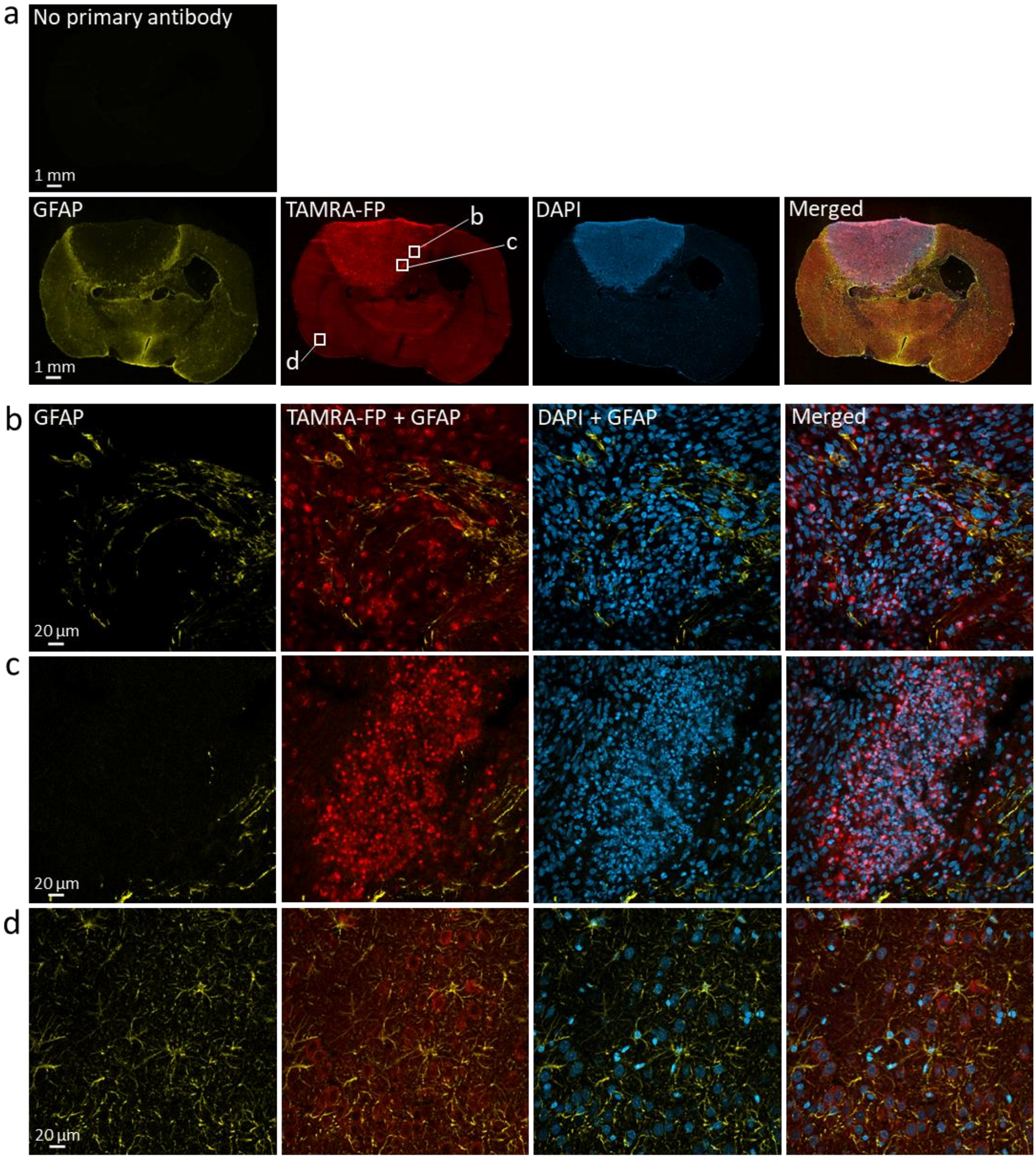

Figure S9. Confocal imaging of SH activity in relation to astrocytes. Sections went through the tissue-ABPP protocol to label SHs (red) and were thereafter immunostained for GFAP to visualize astrocytes (yellow), followed by DAPI staining to visualize nuclei (blue). Panel **a** shows overall staining pattern throughout the coronal section plane. A control section undergoing identical staining protocol with no primary antibody is illustrated at top. Panel **b** shows staining pattern in glioma region characterized by intense SH activity originating from individual cells (TAMRA-FP hotspots). Panel **c** shows staining pattern in glioma region characterized by intense SH activity originating from cell clusters (TAMRA-FP hotspot clusters). Panel **d** shows staining pattern in control region (basal forebrain). Note dense astrocyte population encircling the tumor (**a**). Note sparse overall presence of astrocytes in the tumor (**a**) and their presence in the tumor margin (**b**). Note poor co-localization of GFAP-positive cells with TAMRA-FP hotspots (**b**). Note also lack of astrocytes from TAMRA-FP hotspot clusters (**c**). In healthy brain, star-shaped cells (i.e. astrocytes) are clearly visible (**d**). Magnification 10x in **a**, 40x in **b-d**. Primary antibody goat anti-GFAP (Abcam, cat# ab53554), dilution 1:500, secondary antibody donkey anti-goat IgG-Alexa Fluor 647 conjugate, dilution 1:500. Sections were from male rat 33. Scale bars: 1 mm in a, 20 µm in b-d. Images were adjusted for brightness and contrast.

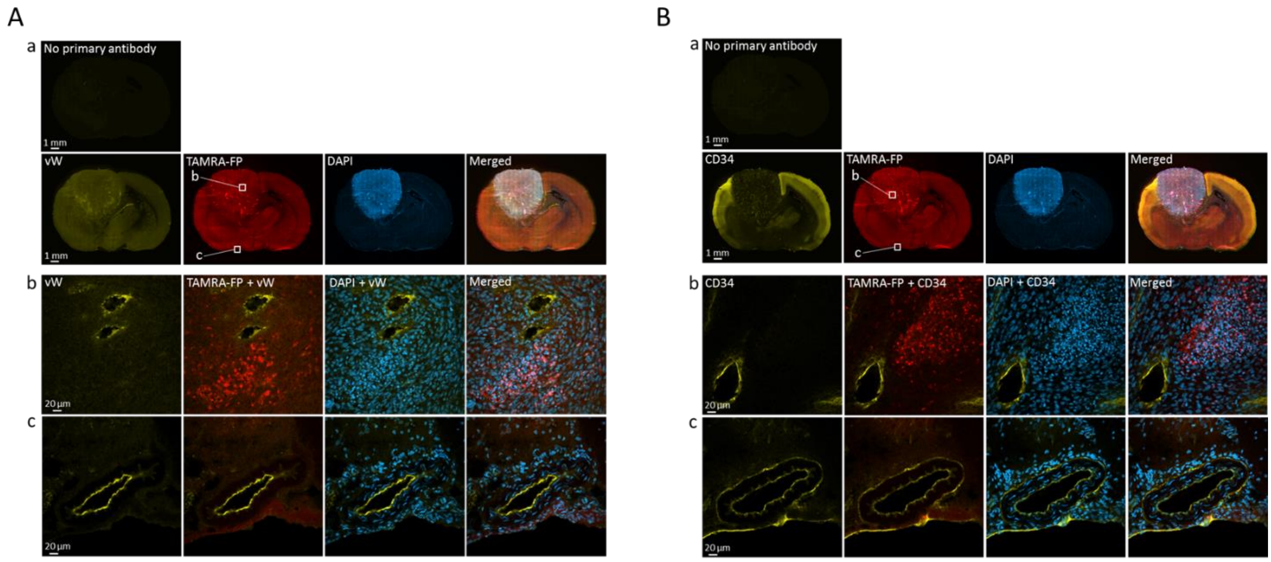

Figure S10. Confocal imaging of SH activity in relation to blood vessels. Vasculature was visualized using the endothelial markers von Willebrand factor (vW) and CD34. Sections went through the tissue-ABPP protocol to label SHs (red) and were thereafter immunostained for vW (**A**) or CD34 (**B**) to visualize endothelial cells (yellow), followed by DAPI staining to visualize nuclei (blue). Panel **a** shows overall staining pattern throughout the coronal section plane. A control section undergoing identical staining protocol with no primary antibody is illustrated at top. Panel **b** shows staining pattern in glioma region with intense SH activity (TAMRA-FP hotspot clusters). Panel **c** shows staining pattern in control brain (hypotalamus). Note intense labeling of endothelial cells lining the vessels by both markers (**b**). Note TAMRA-FP hotspot clusters in close vicinity of the vessels (**b**). In healthy brain (**c**), endothelial cells lining the vessels are labeled by both markers. Magnification 10x in **a**, 40x in **b-c**. In **A**, primary antibody rabbit anti-von Willebrand Factor (Abcam, cat# ab6994), dilution 1:2000, secondary antibody goat anti-rabbit IgG-Alexa Fluor 647 conjugate, dilution 1:500. Sections were from female rat 11. In **B**, primary antibody goat anti-CD34 (R&D systems, cat# AF4117), dilution 1:500, secondary antibody donkey anti-goat IgG-Alexa Fluor 647 conjugate, dilution 1:100. Sections were from female rat 11. Scale bars: 1 mm in a, 20 µm in b-c. Images were adjusted for brightness and contrast.

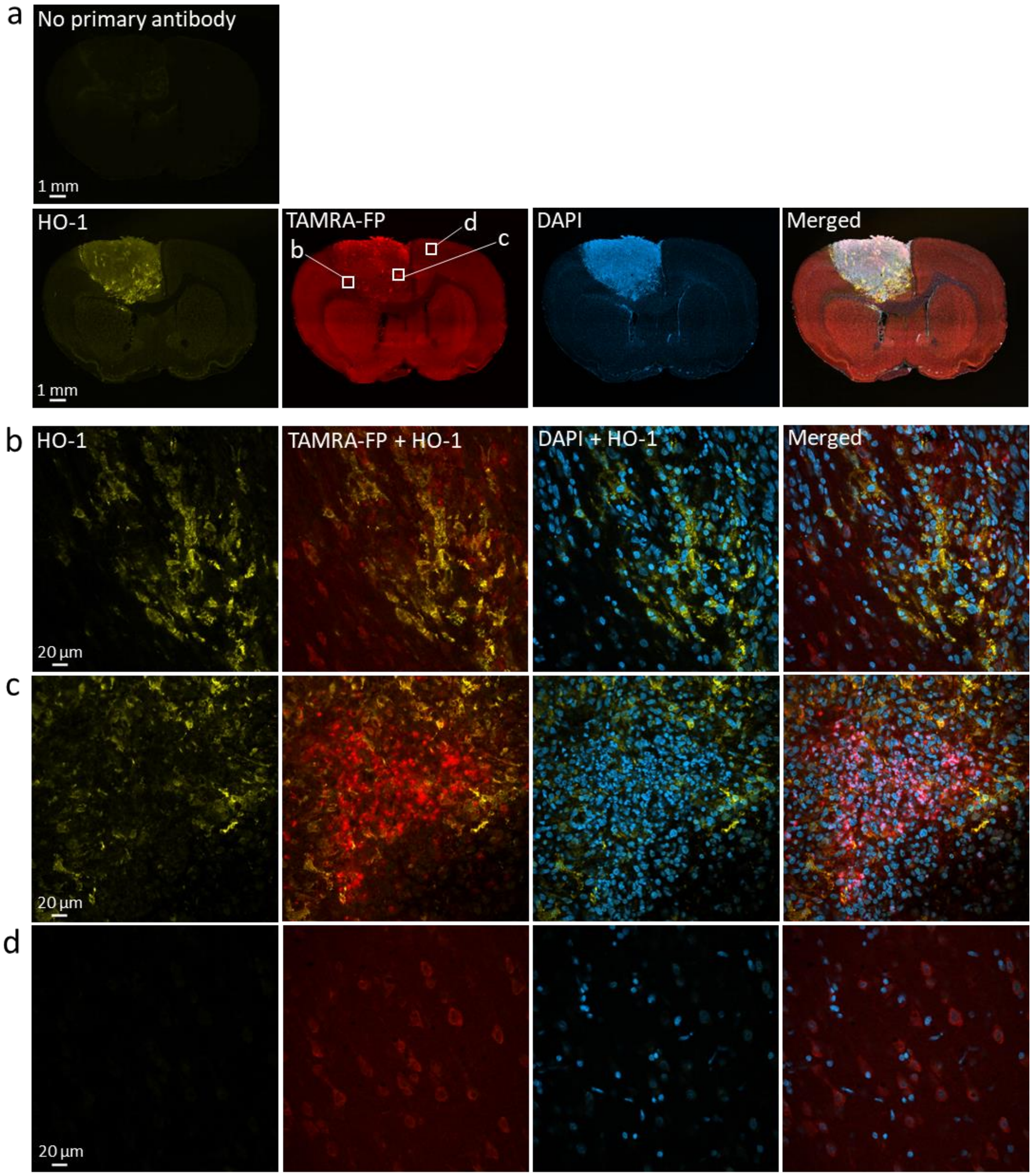

Figure S11. Confocal imaging of SH activity in relation to heme oxygenase 1 (HO-1). Sections went through the tissue-ABPP protocol to label SHs (red) and were thereafter immunostained for HO-1 to visualize redox-sensitive areas (yellow), followed by DAPI staining to visualize nuclei (blue). Panel **a** shows overall staining pattern throughout the coronal section plane. A control section undergoing identical staining protocol with no primary antibody is illustrated at top. Panel **b** shows staining pattern in glioma region characterized by intense SH activity originating from individual cells (TAMRA-FP hotspots). Panel **c** shows staining pattern in glioma region characterized by intense SH activity originating from cell clusters (TAMRA-FP hotspot clusters). Panel **d** shows staining pattern in healthy brain (cortex). Note heterogeneous pattern of HO-1 expression over the tumor and its absence from healthy brain. Note poor co-localization of TAMRA-FP labeling with the stress marker. Primary antibody mouse anti-heme oxygenase 1 (GTS-1, Abcam, cat# ab12220), dilution 1:500, secondary antibody donkey anti-mouse IgG-Alexa Fluor 647 conjugate, dilution 1:100. Sections were from male rat 29. Scale bars: 1 mm in a, 20 µm in b-d. Images were adjusted for brightness and contrast.

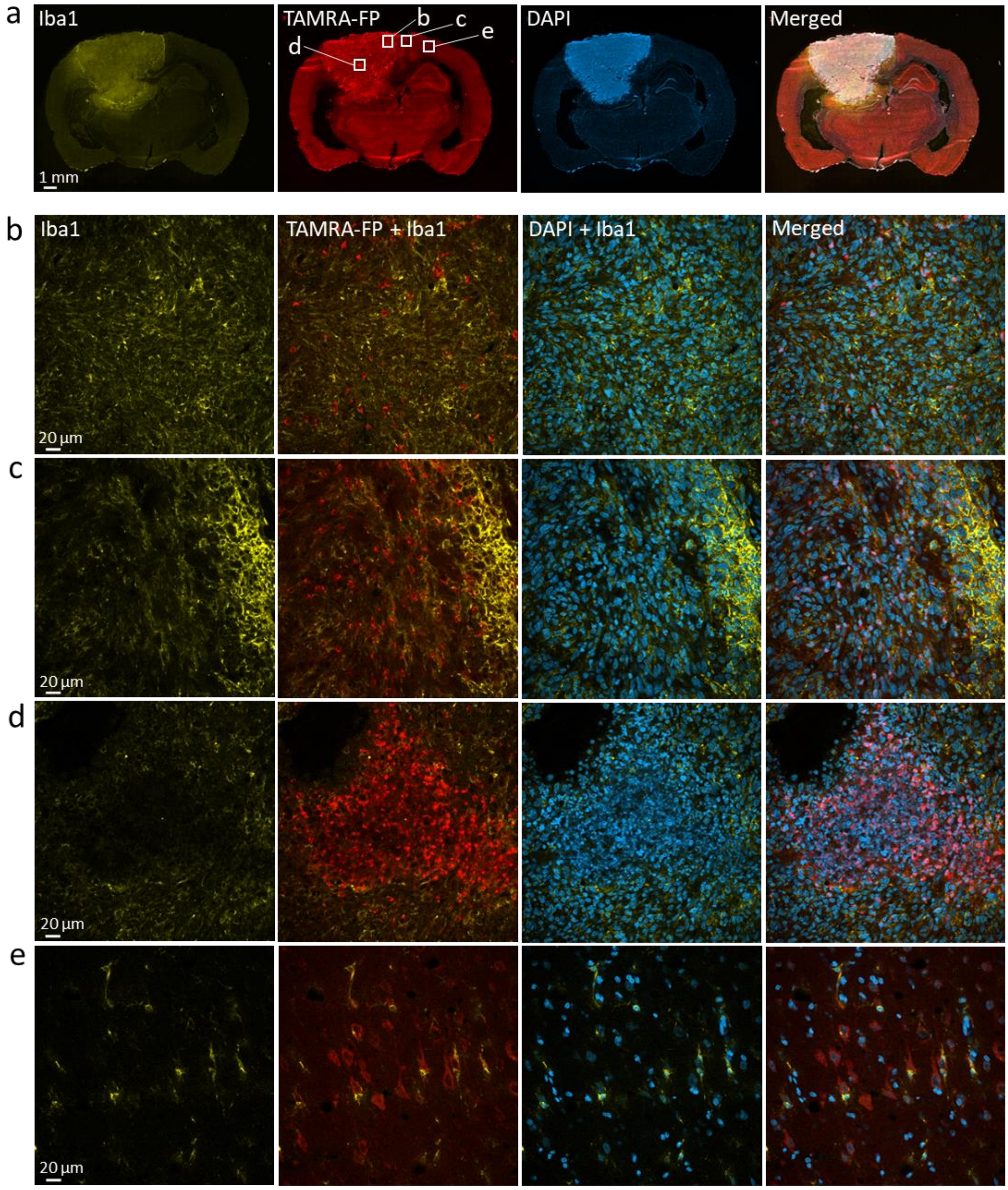

Figure S12. Confocal imaging of SH activity in relation to microglial marker Iba1. Sections went through the tissue-ABPP protocol to label SHs (red) and were thereafter immunostained for Iba1 to visualize microglia (yellow), followed by DAPI staining to visualize nuclei (blue). Panel **a** shows overall staining pattern throughout the coronal section plane. Due to technical problem, a control section without primary antibody is not available for this experiment. Panel **b** shows staining pattern in glioma region characterized by intense SH activity originating from individual cells (TAMRA-FP hotspots). Panel **c** shows staining pattern in glioma border with more Iba1-positive cells residing on the healthy side. Panel **d** shows staining pattern in glioma region characterized by intense SH activity originating from cell clusters (TAMRA-FP hotspot clusters). Panel **e** shows staining pattern in control brain (cortex). Note heterogeneous presence of Iba1-positive cells throughout the tumor (**a**) and especially its border (**c**). Note partial overlap of Iba1 staining with TAMRA-FP hotspots (**b**). In contrast, no Iba1-positive cells are visible in region of TAMRA-FP hotspot clusters (**d**). In control brain, Iba1-positive cells with characteristic microglia morphology and low TAMRA-FP fluorescence are visible. Primary antibody goat anti-Iba1 (Abcam, cat# ab5076), dilution 1:1000, secondary antibody donkey anti-goat IgG-Alexa Fluor 647 conjugate, dilution 1:100. Sections were from female rat 11. Scale bars: 1 mm in a, 20 µm in b-d. Images were adjusted for brightness and contrast.

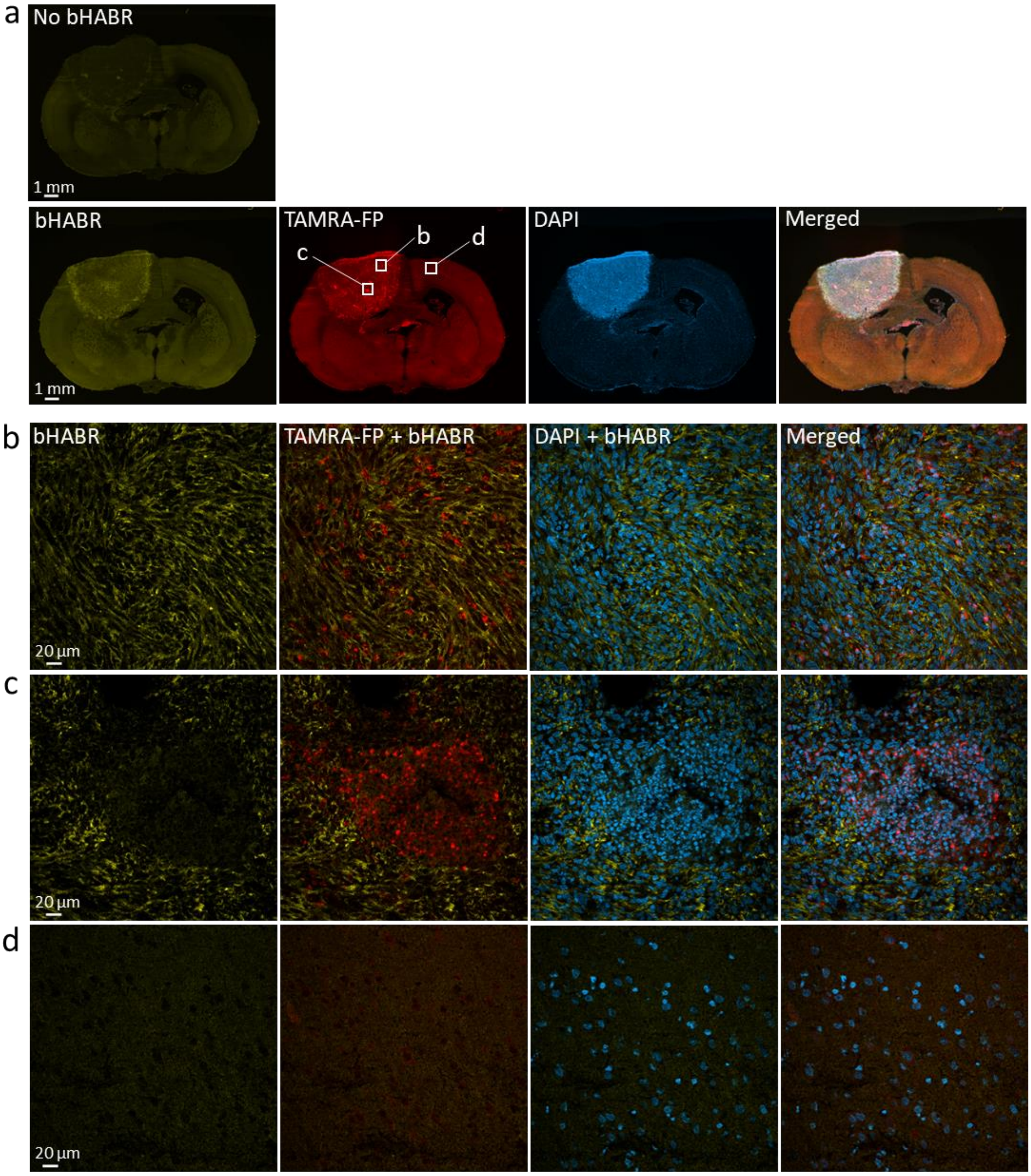

Figure S13. Confocal imaging of SH activity in relation to hyaluronan (HA). Sections went through the tissue-ABPP protocol to label SHs (red) and were thereafter stained for HA (yellow), followed by DAPI staining to visualize nuclei (blue). Panel **a** shows overall staining pattern throughout the coronal section plane. A control section undergoing identical staining protocol with no bHABR is illustrated at top. Panel **b** shows staining pattern in glioma region characterized by intense SH activity originating from individual cells (TAMRA-FP hotspots). Panel **c** shows staining pattern in glioma region characterized by intense SH activity originating from cell clusters (TAMRA-FP hotspot clusters). Panel **d** shows staining pattern in healthy brain (cortex). Note abundance of HA in the extracellular matrix throughout the brain with more intense staining over glioma, notably glioma edges. Note presence of HA in region of TAMRA-FP hotspots (**b**). Note also absence of HA from TAMRA-FP hotspot clusters, as well as encircling of these clusters by HA-enriched matrix (**c**). Relatively low abundance of HA is evident in healthy brain (**d**). HA-binding probe (bHABR, prepared in-house), dilution 1:30, secondary antibody DyLight488 streptavidin, dilution 1:900. Sections were from male rat 34. Scale bars: 1 mm in a, 20 µm in b-d. Images were adjusted for brightness and contrast.

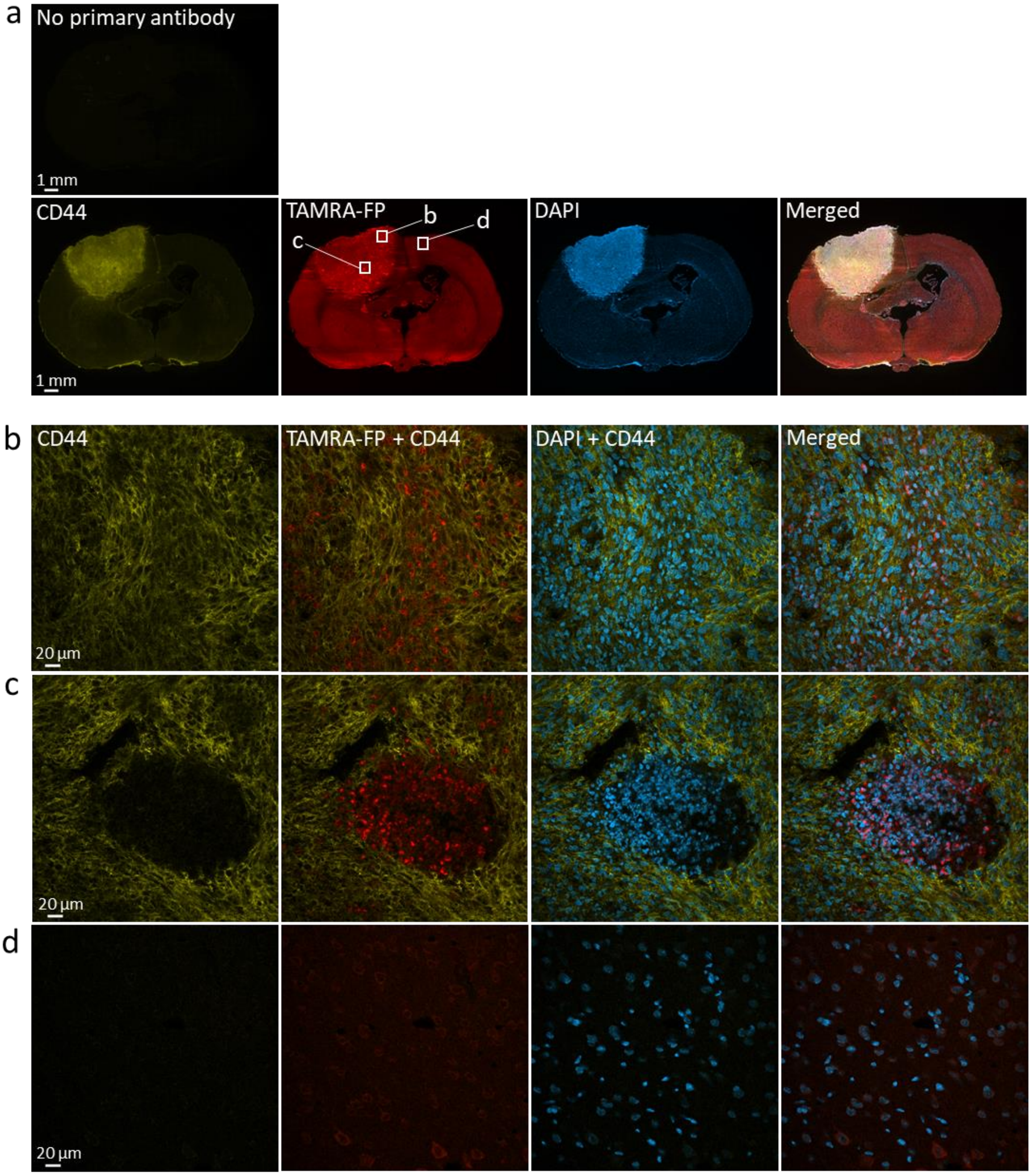

Figure S14. Confocal imaging of SH activity in relation to HA receptor CD44. Sections went through the tissue-ABPP protocol to label SHs (red) and were thereafter immunostained for CD44 (yellow), followed by DAPI staining to visualize nuclei (blue). Panel **a** shows overall staining pattern throughout the coronal section plane. A control section undergoing identical staining protocol with no primary antibody is illustrated at top. Panel **b** shows staining pattern in glioma region characterized by intense SH activity originating from individual cells (TAMRA-FP hotspots). Panel **c** shows staining pattern in glioma region characterized by intense SH activity originating from cell clusters (TAMRA-FP hotspot clusters). Panel **d** shows staining pattern in healthy brain (cortex). Note wide, heterogeneous distribution of CD44 in glioma. Note CD44-positive cells in region of TAMRA-FP hotspots (**b**). Note absence of CD44 from TAMRA-FP hotspot clusters (**c**). Note also that these clusters are surrounded by CD44-positive cells (**c**). CD44 is undetectable in healthy brain (**d**). Primary antibody rabbit anti-CD44 (Abcam, cat# ab157107), dilution 1:2000, secondary antibody goat anti-rabbit IgG-Alexa Fluor 647 conjugate, dilution 1:100. Sections were from male rat 34. Scale bars: 1 mm in a, 20 µm in b-d. Images were adjusted for brightness and contrast.

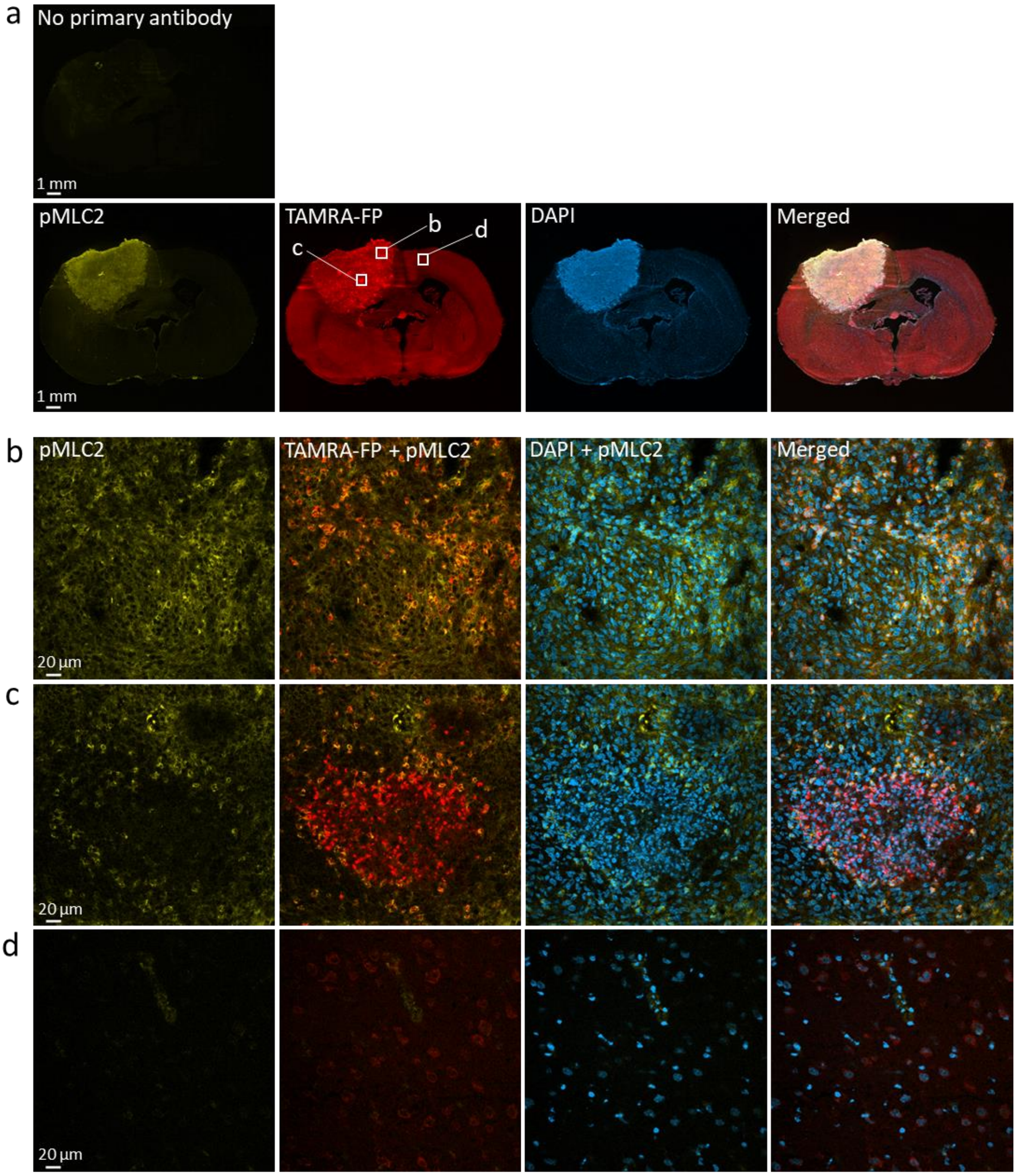

Figure S15. Confocal imaging of SH activity in relation to the stiffness marker pMLC2. Sections went through the tissue-ABPP protocol to label SHs (red) and were thereafter immunostained for pMLC2 (yellow), followed by DAPI staining to visualize nuclei (blue). Panel **a** shows overall staining pattern throughout the coronal section plane. A control section undergoing identical staining protocol with no primary antibody is illustrated at top. Panel **b** shows staining pattern in glioma region characterized by intense SH activity originating from individual cells (TAMRA-FP hotspots). Panel **c** shows staining pattern in glioma region characterized by intense SH activity originating from cell clusters (TAMRA-FP hotspot clusters). Panel **d** shows staining pattern in healthy brain (cortex). Note abundance of pMLC2 throughout the glioma (**a**) and that the stiffness marker co-localizes with TAMRA-FP hotspots (**b**). Note low pMLC2 expression within the TAMRA-FP hotspot clusters (**c**). Note also that the TAMRA-FP positive cluster rim and cells surrounded by the cluster contain pMLC2-positive cells (**c**). pMLC2 is undetectable in cortical control region (**d**). Primary antibody mouse anti-Phospho-Myosin Light Chain 2 (pMLC2, Cell Signaling Technology, cat# 3675s), dilution 1:50, secondary antibody donkey anti-mouse IgG-Alexa Fluor 647 conjugate, dilution 1:100. Sections were from male rat 34. Scale bars: 1 mm in a, 20 µm in b-d. Images were adjusted for brightness and contrast.

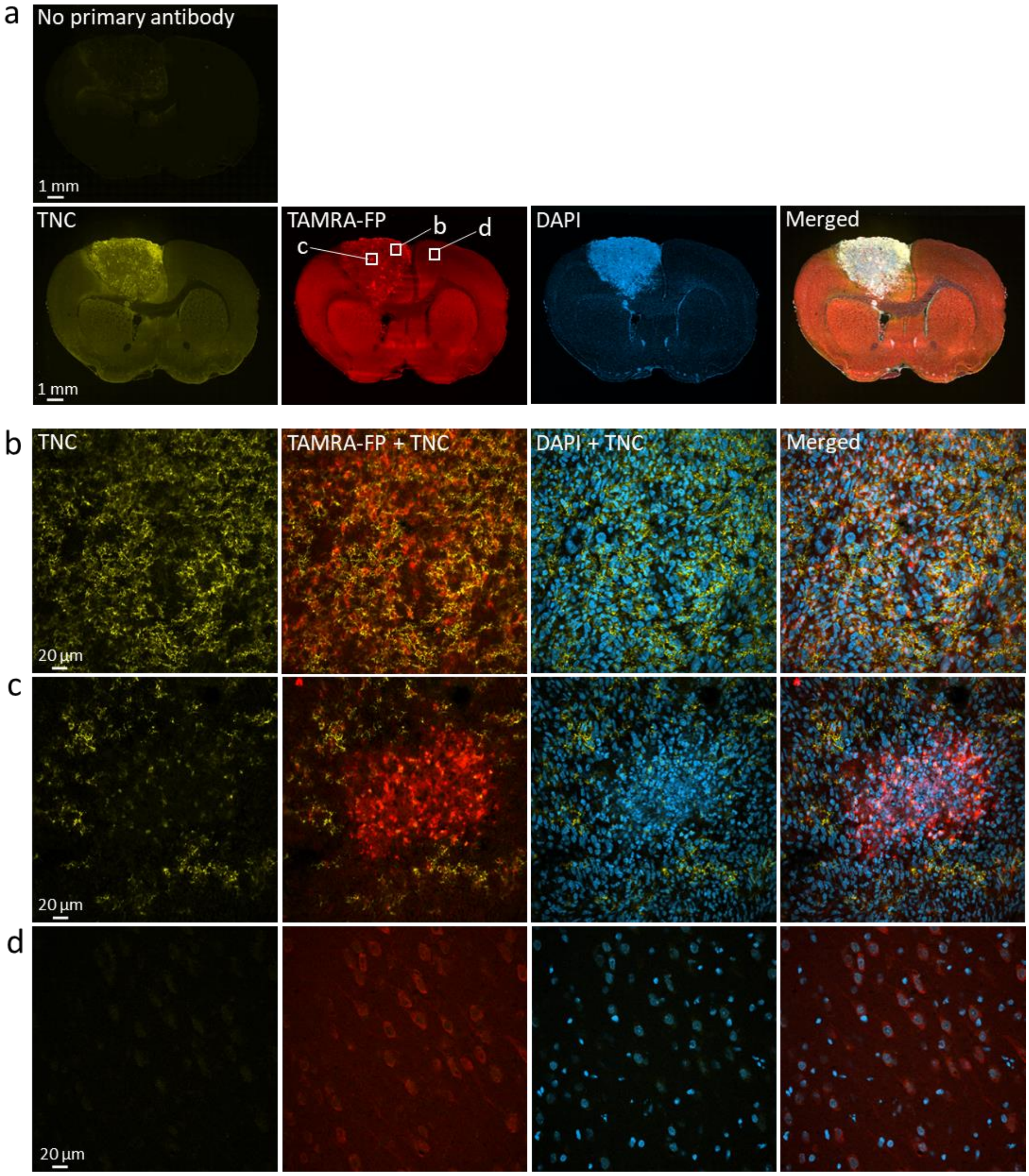

Figure S16. Confocal imaging of SH activity in relation to the stiffness marker tenascin C. Sections went through the tissue-ABPP protocol to label SHs (red) and were thereafter immunostained for tenascin C (TNC, yellow), followed by DAPI staining to visualize nuclei (blue). Panel **a** shows overall staining pattern throughout the coronal section plane. A control section undergoing identical staining protocol with no primary antibody is illustrated at top. Panel **b** shows staining pattern in glioma region characterized by intense SH activity originating from individual cells (TAMRA-FP hotspots). Panel **c** shows staining pattern in glioma region characterized by intense SH activity originating from cell clusters (TAMRA-FP hotspot clusters). Panel **d** shows staining pattern in healthy brain (cortex). Note heterogeneous expression of tenascin C in the glioma (**a**) and that tenascin C co-localizes with TAMRA-FP hotspots (**b**). Note low tenascin C expression in TAMRA-FP hotspot clusters and that the cells around these clusters frequently express tenascin C (**c**). Tenascin C is undetectable in cortical control region (**d**). Primary antibody mouse anti-Tenascin C (Thermo Scientific, cat# MA5-16086), dilution 1:100, secondary antibody donkey anti-mouse IgG-Alexa Fluor 647 conjugate, dilution 1:100. Sections were from male rat 29. Scale bars: 1 mm in a, 20 µm in b-d. Images were adjusted for brightness and contrast.

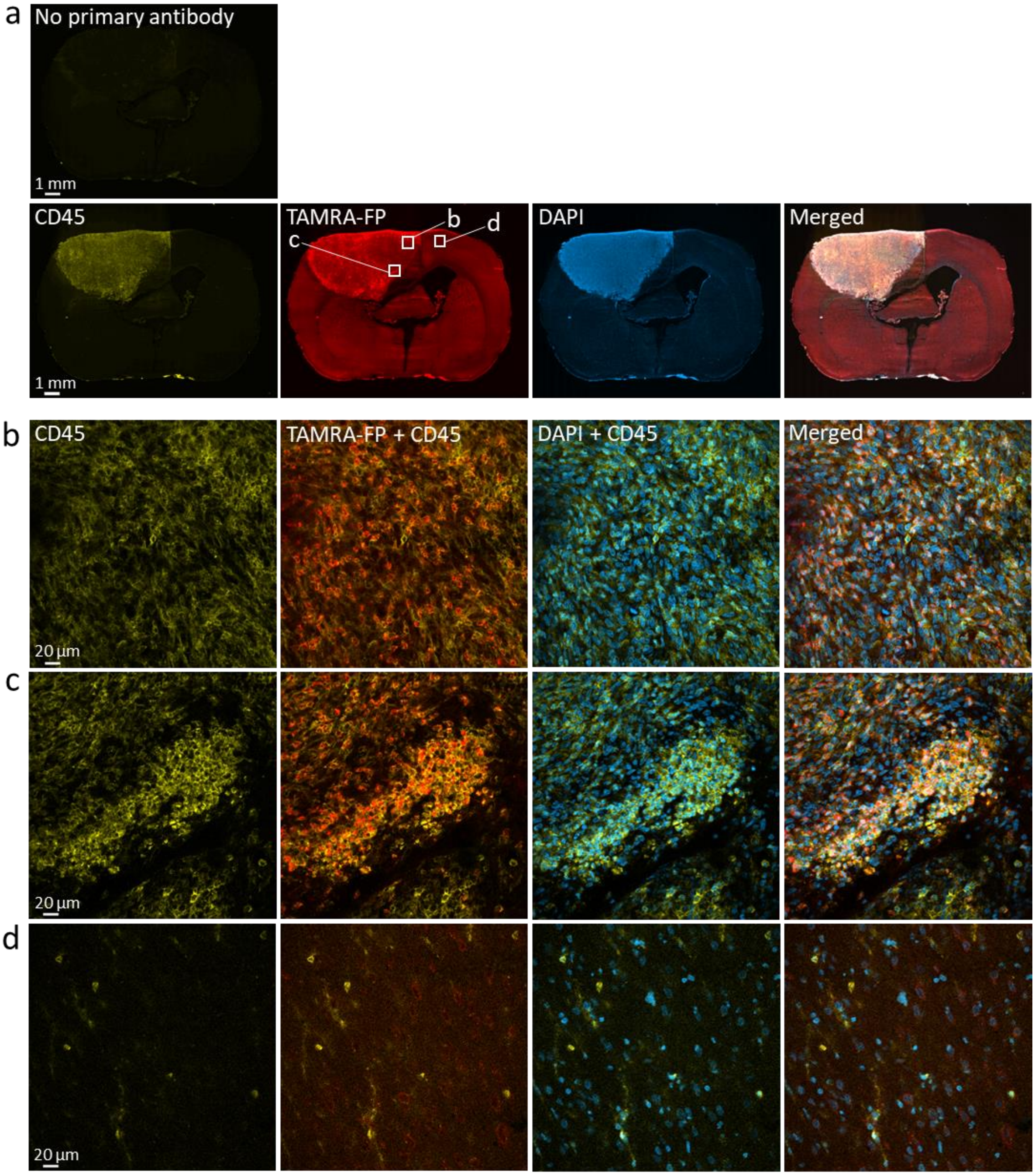

Figure S17. Confocal imaging of SH activity in relation to CD45, a marker for nucleated hematopoietic cells. Sections went through the tissue-ABPP protocol to label SHs (red) and were thereafter immunostained for CD45 (yellow), followed by DAPI staining to visualize nuclei (blue). Panel **a** shows overall staining pattern throughout the coronal section plane. A control section undergoing identical staining protocol with no primary antibody is illustrated at top. Panel **b** shows staining pattern in glioma region characterized by intense SH activity originating from individual cells (TAMRA-FP hotspots). Panel **c** shows staining pattern in glioma region characterized by intense SH activity originating from cell clusters (TAMRA-FP hotspot clusters). Panel **d** shows staining pattern in healthy brain (cortex). Note ample expression of CD45 in the glioma (**a**) and that this marker shows co-localization with TAMRA-FP hotspots (**b**). Note also abundance of CD45 and its co-localization with TAMRA-FP hotspot clusters (**c**). Sparse immunostaining is visible in control cortical region (**d**). Primary antibody mouse anti-CD45 (MRC-OX1, Abcam, ab33923), dilution 1:500, secondary antibody donkey anti-mouse IgG-Alexa Fluor 647 conjugate, dilution 1:100. Sections were from male rat 33. Scale bars: 1 mm in a, 20 µm in b-d. Images were adjusted for brightness and contrast.

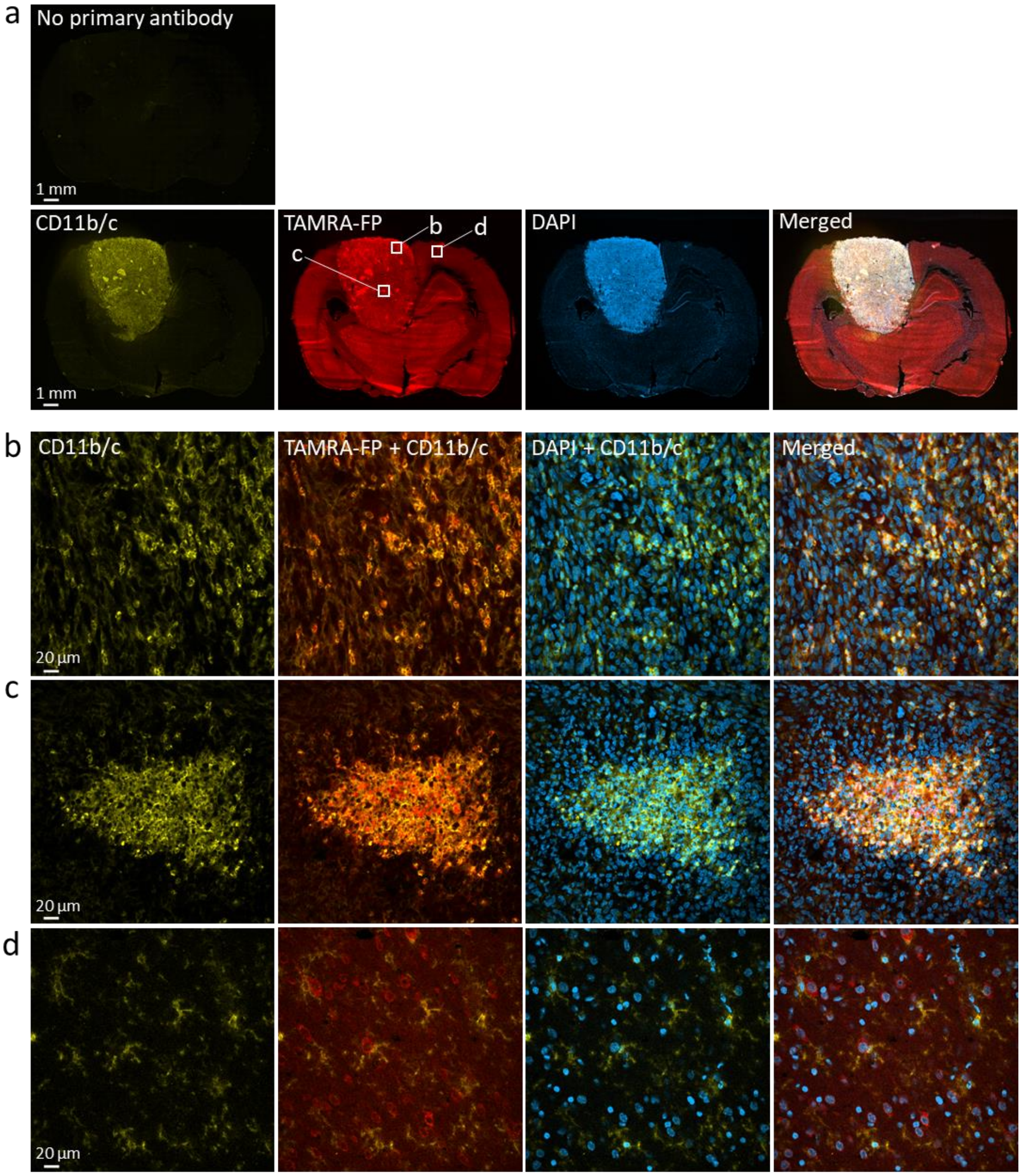

Figure S18. Confocal imaging of SH activity in relation to CD11b/c, a marker for phagocytes. Sections went through the tissue-ABPP protocol to label SHs (red) and were thereafter immunostained for CD11b/c (yellow), followed by DAPI staining to visualize nuclei (blue). Panel **a** shows overall staining pattern throughout the coronal section plane. A control section undergoing identical staining protocol with no primary antibody is illustrated at top. Panel **b** shows staining pattern in glioma region characterized by intense SH activity originating from individual cells (TAMRA-FP hotspots). Panel **c** shows staining pattern in glioma region characterized by intense SH activity originating from cell clusters (TAMRA-FP hotspot clusters). Panel **d** shows staining pattern in healthy brain (cortex). Note ample expression of CD11b/c in the glioma (**a**) and that this marker co-localizes with TAMRA-FP hotspots (**b**). Note also high CD11b/c expression and co-localization with TAMRA-FP hotspot clusters (**c**). CD11b/c-positive cells with characteristic microglial morphology are visible in cortical control region (**d**). Primary antibody mouse anti-CD11b/c (OX42, Abcam, ab1211), dilution 1:1000, secondary antibody donkey anti-mouse IgG-Alexa Fluor 647 conjugate, dilution 1:100. Sections were from male rat 29. Scale bars: 1 mm in a, 20 µm in b-d. Images were adjusted for brightness and contrast.

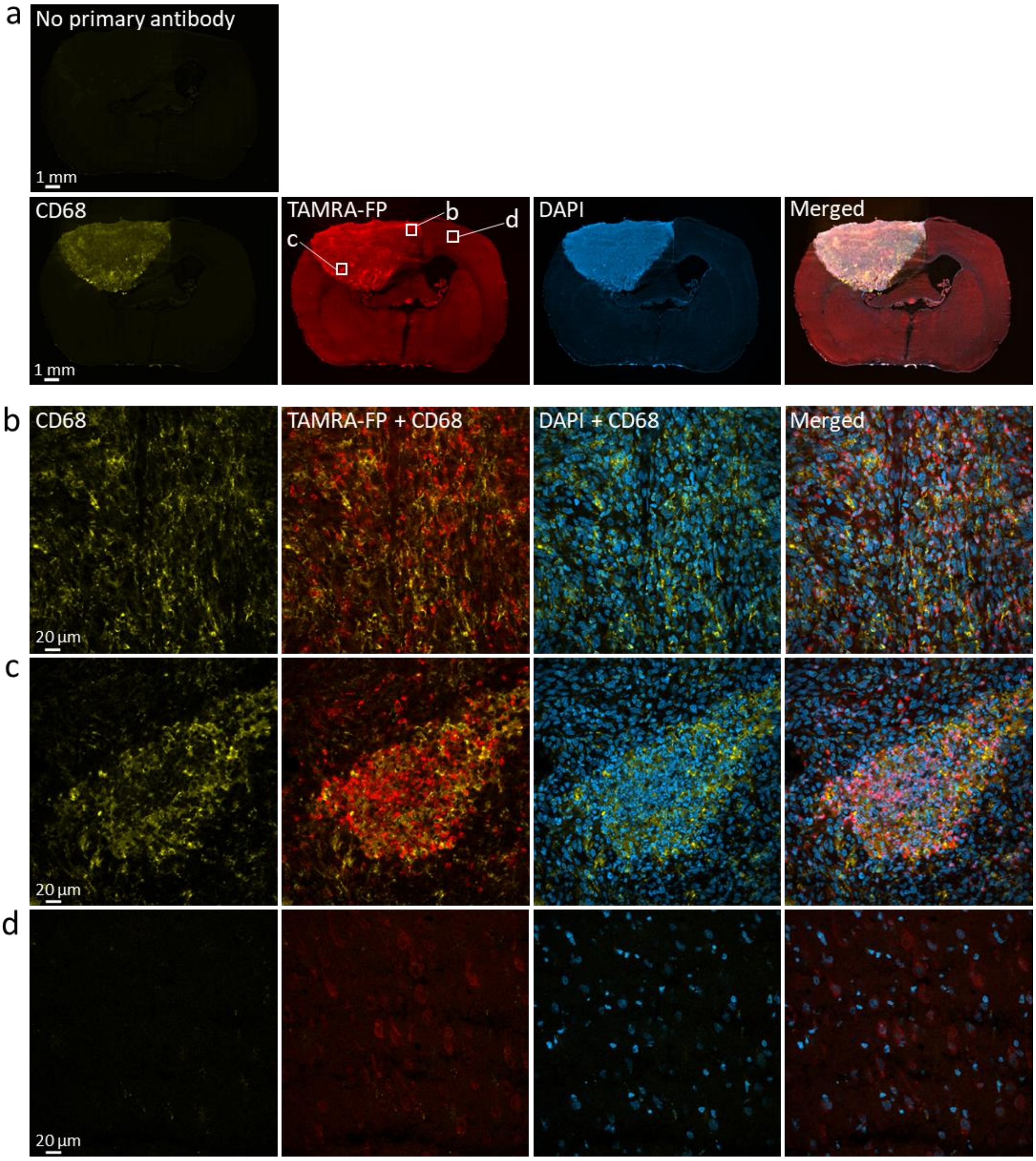

Figure S19. Confocal imaging of SH activity in relation to CD68, a marker for monocytes and macrophages. Sections went through the tissue-ABPP protocol to label SHs (red) and were thereafter immunostained for CD68 (yellow), followed by DAPI staining to visualize nuclei (blue). Panel **a** shows overall staining pattern throughout the coronal section plane. A control section undergoing identical staining protocol with no primary antibody is illustrated at top. Panel **b** shows staining pattern in glioma region characterized by intense SH activity originating from individual cells (TAMRA-FP hotspots). Panel **c** shows staining pattern in glioma region characterized by intense SH activity originating from cell clusters (TAMRA-FP hotspot clusters). Panel **d** shows staining pattern in healthy brain (cortex). Note intense expression of CD68 in the glioma (**a**) and its partial co-localization with TAMRA-FP hotspots (**b**). Note enrichment of CD68 and its partial co-localization with TAMRA-FP hotspot clusters (**c**). CD68 is undetectable in control cortical region (**d**). Primary antibody mouse anti-CD68 (ED1, Bio-Rad, cat# MCA341R), dilution 1:1000, secondary antibody donkey anti-mouse IgG-Alexa Fluor 647 conjugate, dilution 1:100. Sections were from male rat 33. Scale bars: 1 mm in a, 20 µm in b-d. Images were adjusted for brightness and contrast.

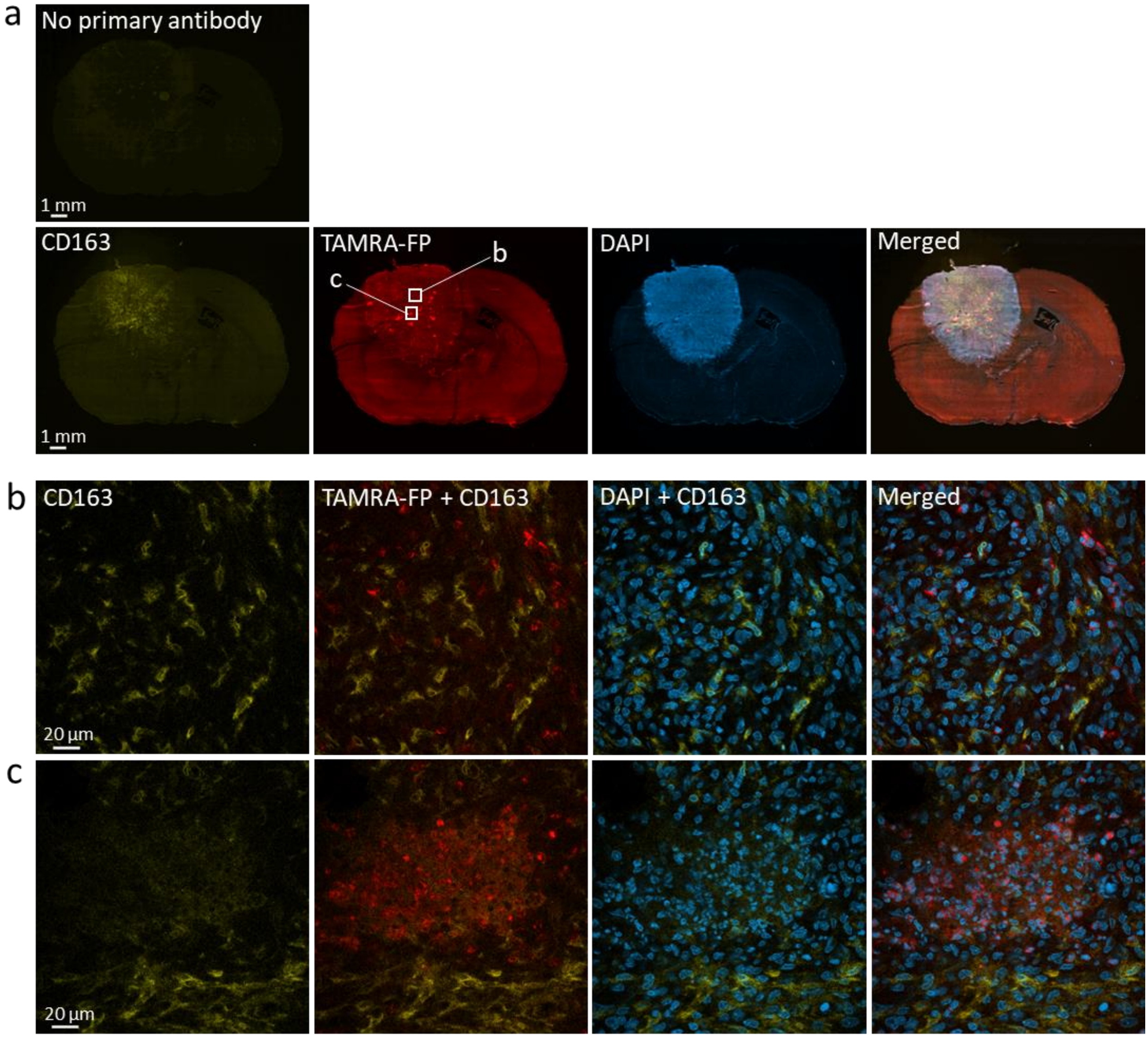

Figure S20. Confocal imaging of SH activity in relation to CD163, a marker for monocytes and macrophages. Sections went through the tissue-ABPP protocol to label SHs (red) and were thereafter immunostained for CD163 (yellow), followed by DAPI staining to visualize nuclei (blue). Panel **a** shows overall staining pattern throughout the coronal section plane. A control section undergoing identical staining protocol with no primary antibody is illustrated at top. Panel **b** shows staining pattern in glioma region characterized by intense SH activity originating from individual cells (TAMRA-FP hotspots). Panel **c** shows staining pattern in glioma region characterized by intense SH activity originating from cell clusters (TAMRA-FP hotspot clusters). Note detectable expression of CD68 in the glioma and its absence from other brain regions (**a**). Note poor co-localization of CD68-positive cells with TAMRA-FP hotspots (**b**) or with TAMRA-FP hotspot clusters (**c**). Primary antibody rabbit anti-CD163 (Abcam, cat# ab182422), dilution 1:75, secondary antibody Goat anti-rabbit IgG-Alexa Fluor 647 conjugate, dilution 1:100. Sections were from female rat 11. Scale bars: 1 mm in a, 20 µm in b-c. Images were adjusted for brightness and contrast.

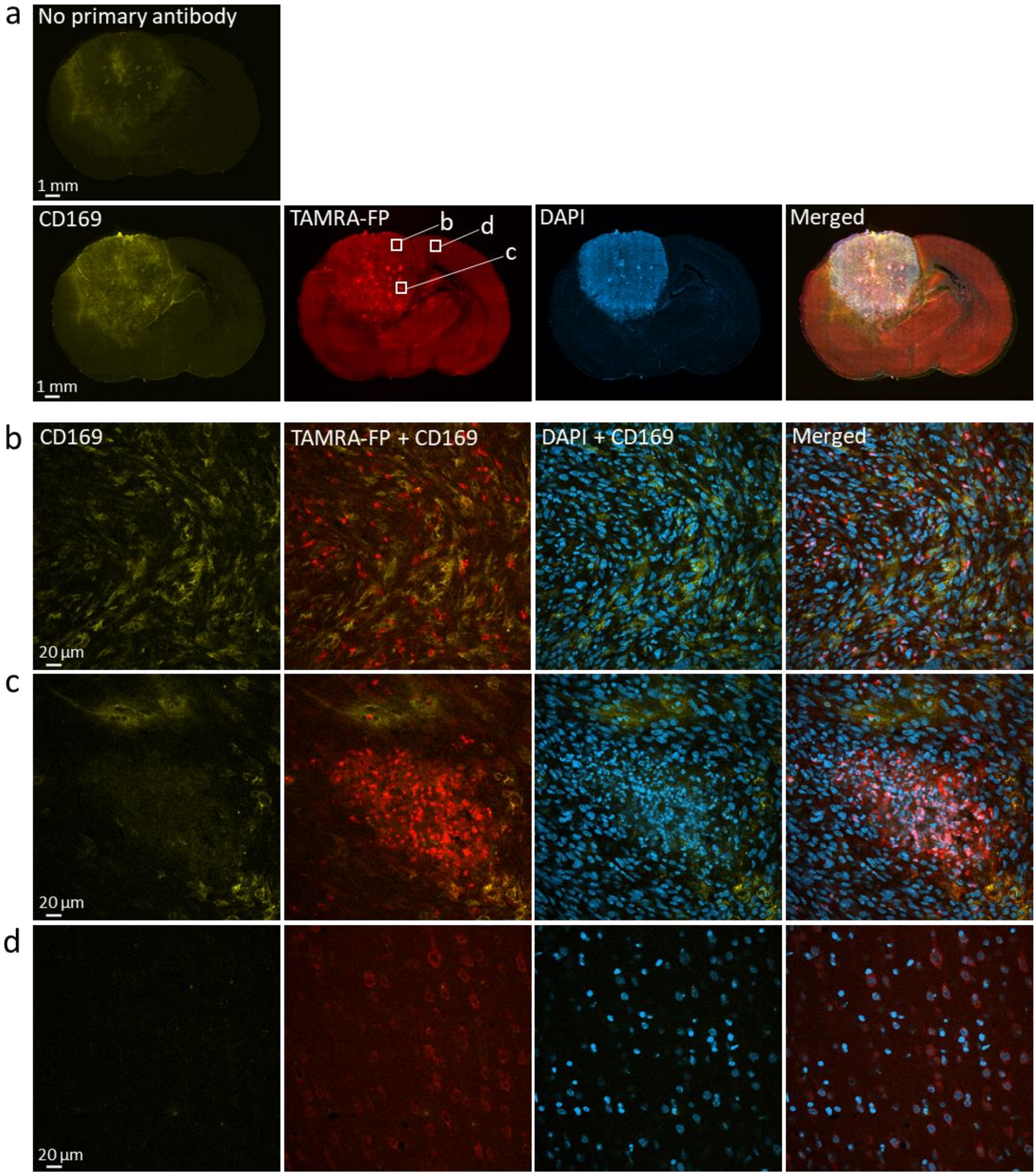

Figure S21. Confocal imaging of SH activity in relation to CD169, a marker for macrophages. Sections went through the tissue-ABPP protocol to label SHs (red) and were thereafter immunostained for CD169 (yellow), followed by DAPI staining to visualize nuclei (blue). Panel **a** shows overall staining pattern throughout the coronal section plane. A control section undergoing identical staining protocol with no primary antibody is illustrated at top. Panel **b** shows staining pattern in glioma region characterized by intense SH activity originating from individual cells (TAMRA-FP hotspots). Panel **c** shows staining pattern in glioma region characterized by intense SH activity originating from cell clusters (TAMRA-FP hotspot clusters). Panel **d** shows staining pattern in healthy brain (cortex). Note detectable expression of CD169 in the glioma (**a**) and its poor co-localization with TAMRA-FP hotspots (**b**). Note lack of CD169 from TAMRA-FP hotspot clusters (**c**). CD169 is undetectable in control cortical region (**d**). Primary antibody mouse anti-CD169 (Bio-Rad, cat# MCA343GA), dilution 1:25, secondary antibody donkey anti-mouse IgG-Alexa Fluor 647 conjugate, dilution 1:100. Sections were from female rat 11. Scale bars: 1 mm in a, 20 µm in b-d. Images were adjusted for brightness and contrast.

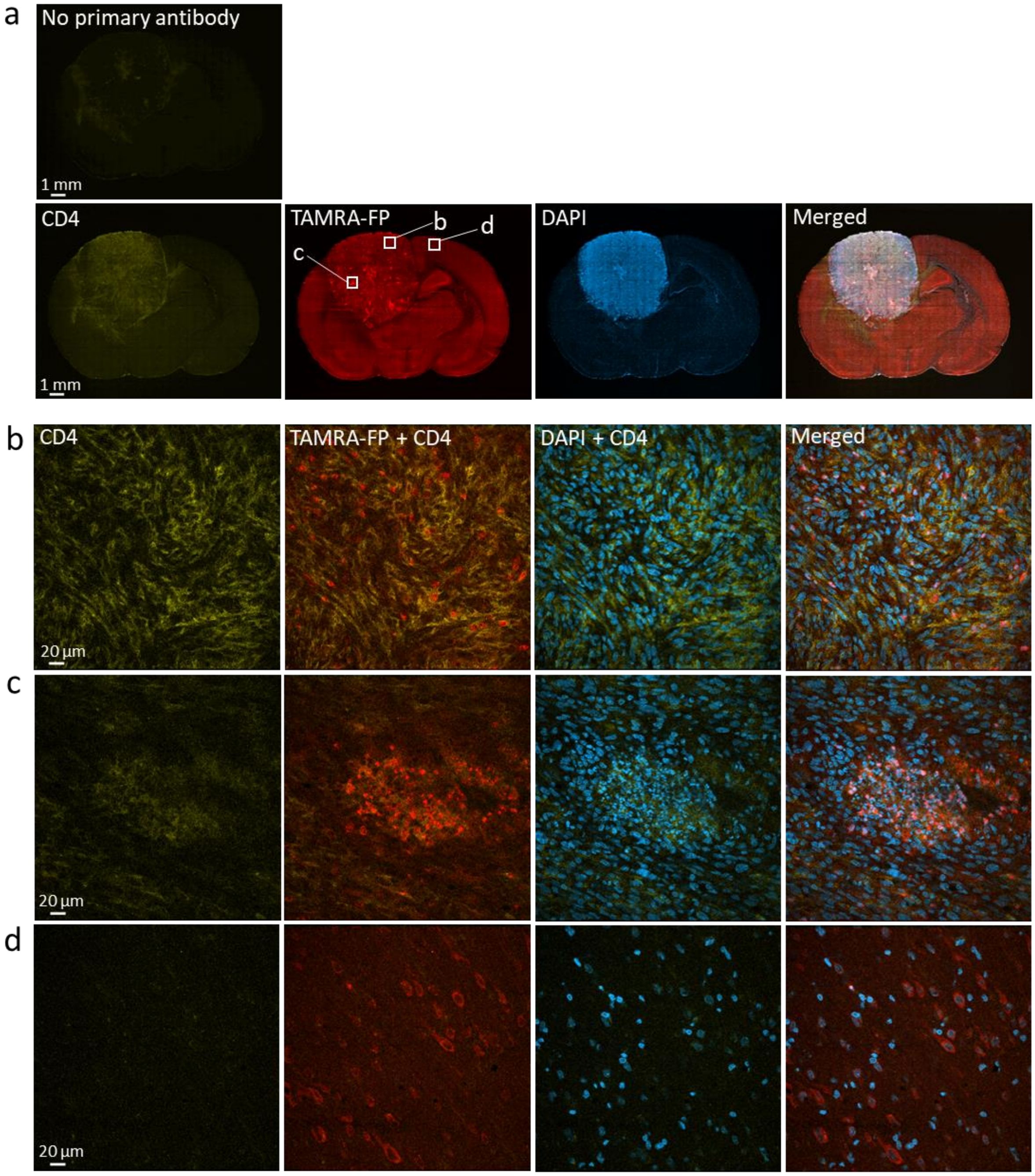

Figure S22. Confocal imaging of SH activity in relation to T cell marker CD4. Sections went through the tissue-ABPP protocol to label SHs (red) and were thereafter immunostained for CD4 (yellow), followed by DAPI staining to visualize nuclei (blue). Panel **a** shows overall staining pattern throughout the coronal section plane. A control section undergoing identical staining protocol with no primary antibody is illustrated at top. Panel **b** shows staining pattern in glioma region characterized by intense SH activity originating from individual cells (TAMRA-FP hotspots). Panel **c** shows staining pattern in glioma region characterized by intense SH activity originating from cell clusters (TAMRA-FP hotspot clusters). Panel **d** shows staining pattern in healthy brain (cortex). Note detectable expression of CD4 in the glioma (**a**) and its absence from other brain regions (**a, d**). Note robust CD4 expression and its partial co-localization with TAMRA-FP hotspots (**b**). Note also CD expression in area with TAMRA-FP hotspot clusters and its partial co-localization with the cluster (**c**). Primary antibody mouse anti-CD4 (OX-35, Abcam, cat# ab33775), dilution 1:100, secondary antibody donkey anti-mouse IgG-Alexa Fluor 647 conjugate, dilution 1:100. Sections were from female rat 11. Scale bars: 1 mm in a, 20 µm in b-d. Images were adjusted for brightness and contrast.

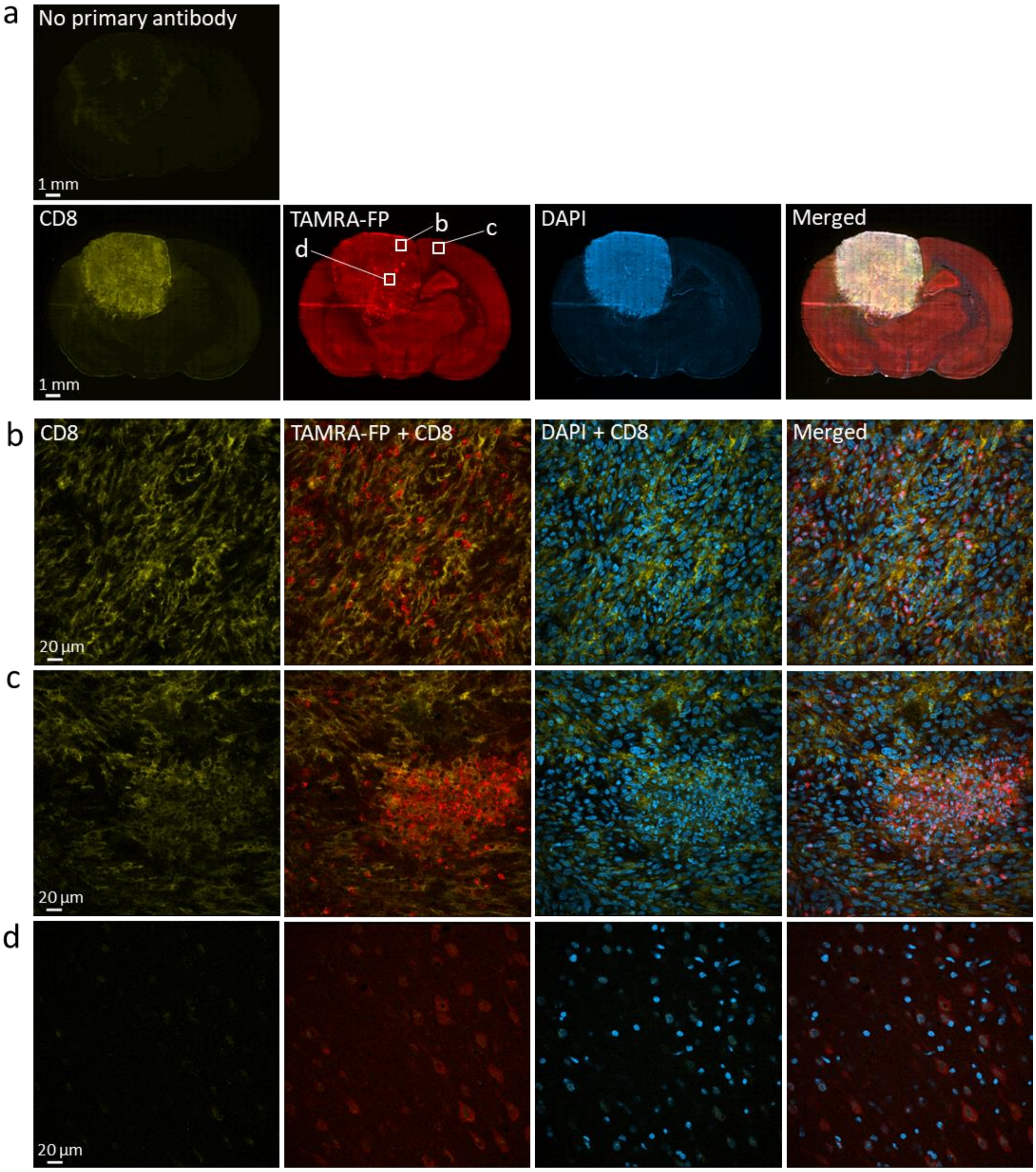

Figure S23. Confocal imaging of SH activity in relation to T cell marker CD8. Sections went through the tissue-ABPP protocol to label SHs (red) and were thereafter immunostained for CD8 (yellow), followed by DAPI staining to visualize nuclei (blue). Panel **a** shows overall staining pattern throughout the coronal section plane. A control section undergoing identical staining protocol with no primary antibody is illustrated at top. Panel **b** shows staining pattern in glioma region characterized by intense SH activity originating from individual cells (TAMRA-FP hotspots). Panel **c** shows staining pattern in glioma region characterized by intense SH activity originating from cell clusters (TAMRA-FP hotspot clusters). Panel **d** shows staining pattern in healthy brain (cortex). Note prominent expression of CD8 throughout the glioma (**a**) and its absence from healthy brain (**a, d**). Note CD8-positive cells and their partial localization to TAMRA-FP hotspots (**b**). Note abundance of CD8-positive cells in the region of TAMRA-FP hotspot clusters, showing partial co-localization with TAMRA-FP signal (**c**). Primary antibody mouse anti-CD8 alpha (OX-8, Abcam, cat# ab33786), dilution 1:500, secondary antibody donkey anti-mouse IgG-Alexa Fluor 647 conjugate, dilution 1:100. Sections were from female rat 11. Scale bars: 1 mm in a, 20 µm in b-d. Images were adjusted for brightness and contrast.

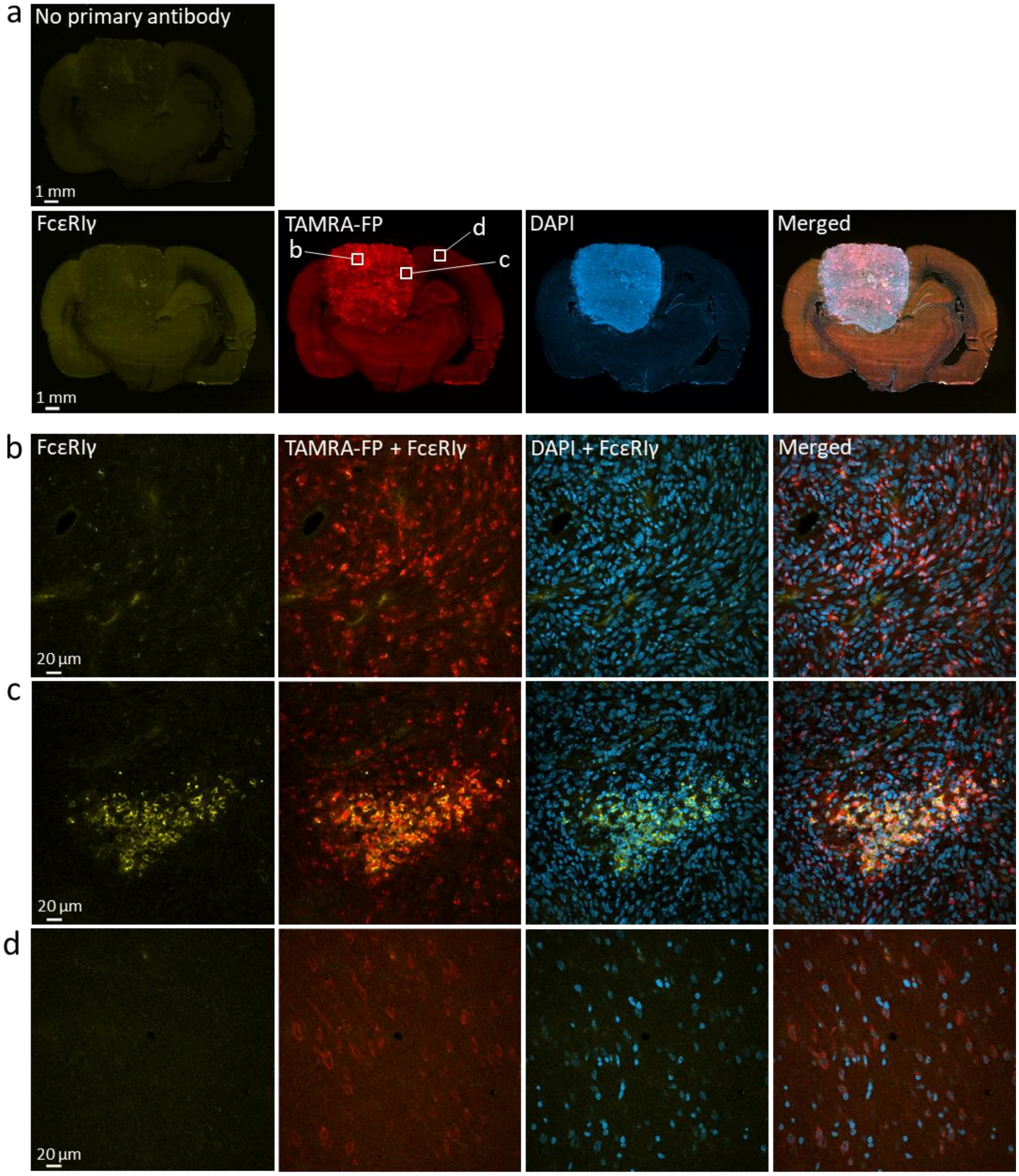

Figure S24. Confocal imaging of SH activity in relation to FcεRIγ, a marker for mast cells, eosinophils, basophils and monocytes. Sections went through the tissue-ABPP protocol to label SHs (red) and were thereafter immunostained for FcεRIγ (yellow), followed by DAPI staining to visualize nuclei (blue). Panel **a** shows overall staining pattern throughout the coronal section plane. A control section undergoing identical staining protocol with no primary antibody is illustrated at top. Panel **b** shows staining pattern in glioma region characterized by intense SH activity originating from individual cells (TAMRA-FP hotspots). Panel **c** shows staining pattern in glioma region characterized by intense SH activity originating from cell clusters (TAMRA-FP hotspot clusters). Panel **d** shows staining pattern in healthy brain (cortex). Note scarce expression of FcεRIγ in the glioma (**a**) and that this marker is practically absent from TAMRA-FP hotspots (**b**). Note enrichment of FcεRIγ and its co-localization with TAMRA-FP hotspot clusters (**c**). FcεRIγ is undetectable in control cortical region (**d**). Primary antibody mouse anti-FcεRIγ (F-1, Santa Cruz, cat# sc-390221), dilution 1:100, secondary antibody donkey anti-mouse IgG-Alexa Fluor 647 conjugate, dilution 1:100. Sections were from male rat 31. Scale bars: 1 mm in a, 20 µm in b-d. Images were adjusted for brightness and contrast.

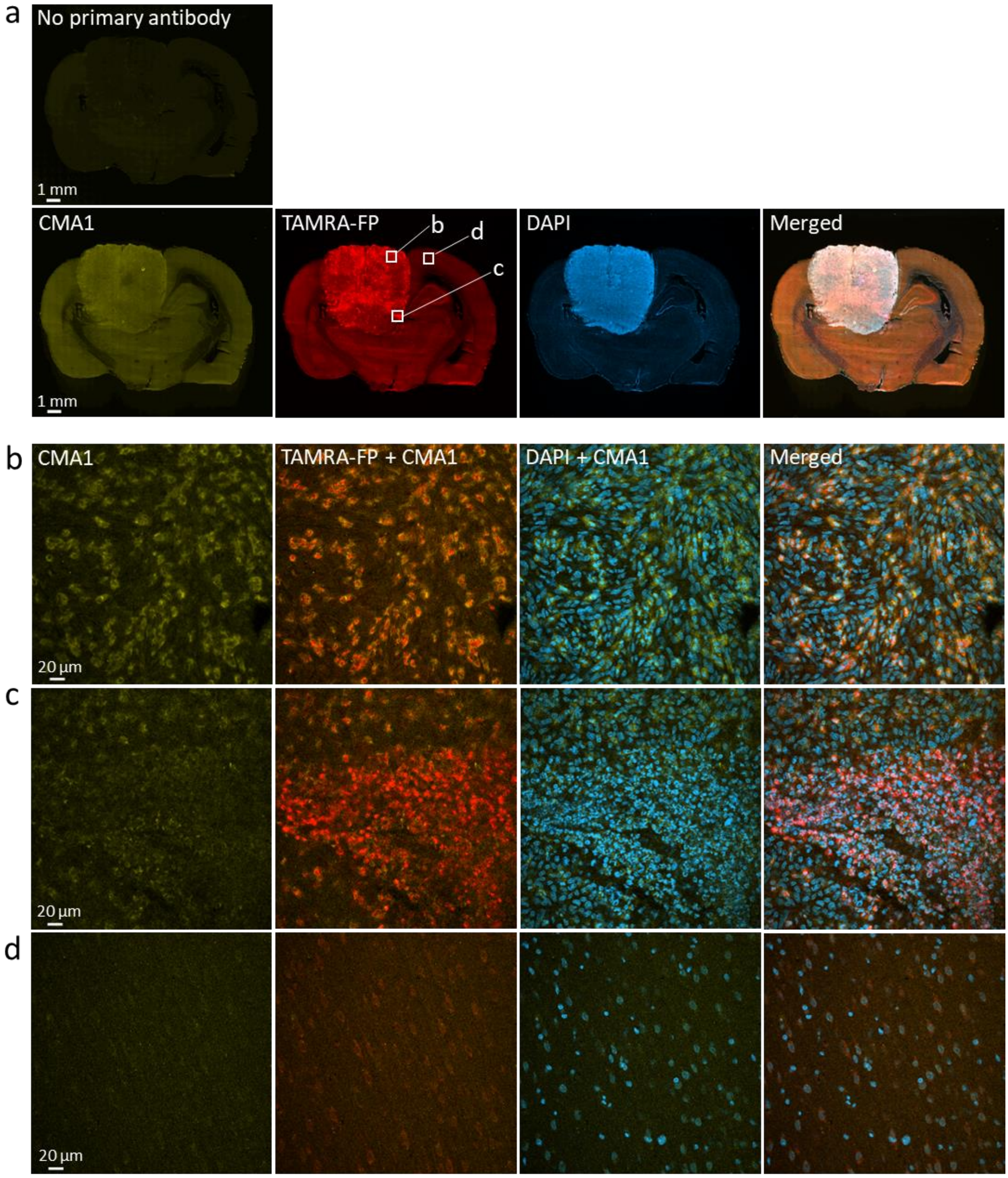

Figure S25. Confocal imaging of SH activity in relation to chymase (CMA1), a marker for mast cells. Sections went through the tissue-ABPP protocol to label SHs (red) and were thereafter immunostained for CMA1 (yellow), followed by DAPI staining to visualize nuclei (blue). Panel **a** shows overall staining pattern throughout the coronal section plane. A control section undergoing identical staining protocol with no primary antibody is illustrated at top. Panel **b** shows staining pattern in glioma region characterized by intense SH activity originating from individual cells (TAMRA-FP hotspots). Panel **c** shows staining pattern in glioma region characterized by intense SH activity originating from cell clusters (TAMRA-FP hotspot clusters). Panel **d** shows staining pattern in healthy brain (cortex). Note faint expression of CMA1 in the glioma (**a**). Note CMA1-positive cells in the region of TAMRA-FP hotspots with notable co-localization with the TAMRA-FP signal (**b**). Note absence of CMA1-positive cells from TAMRA-FP hotspot clusters (**c**). CMA1 is undetectable in control cortical region (**d**). Primary antibody rabbit anti-Chymase 1 (Biomatic, CAU22217), dilution 1:500, secondary antibody goat anti-rabbit IgG-Alexa Fluor 647 conjugate, dilution 1:100. Sections were from male rat 31. Scale bars: 1 mm in a, 20 µm in b-d. Images were adjusted for brightness and contrast.

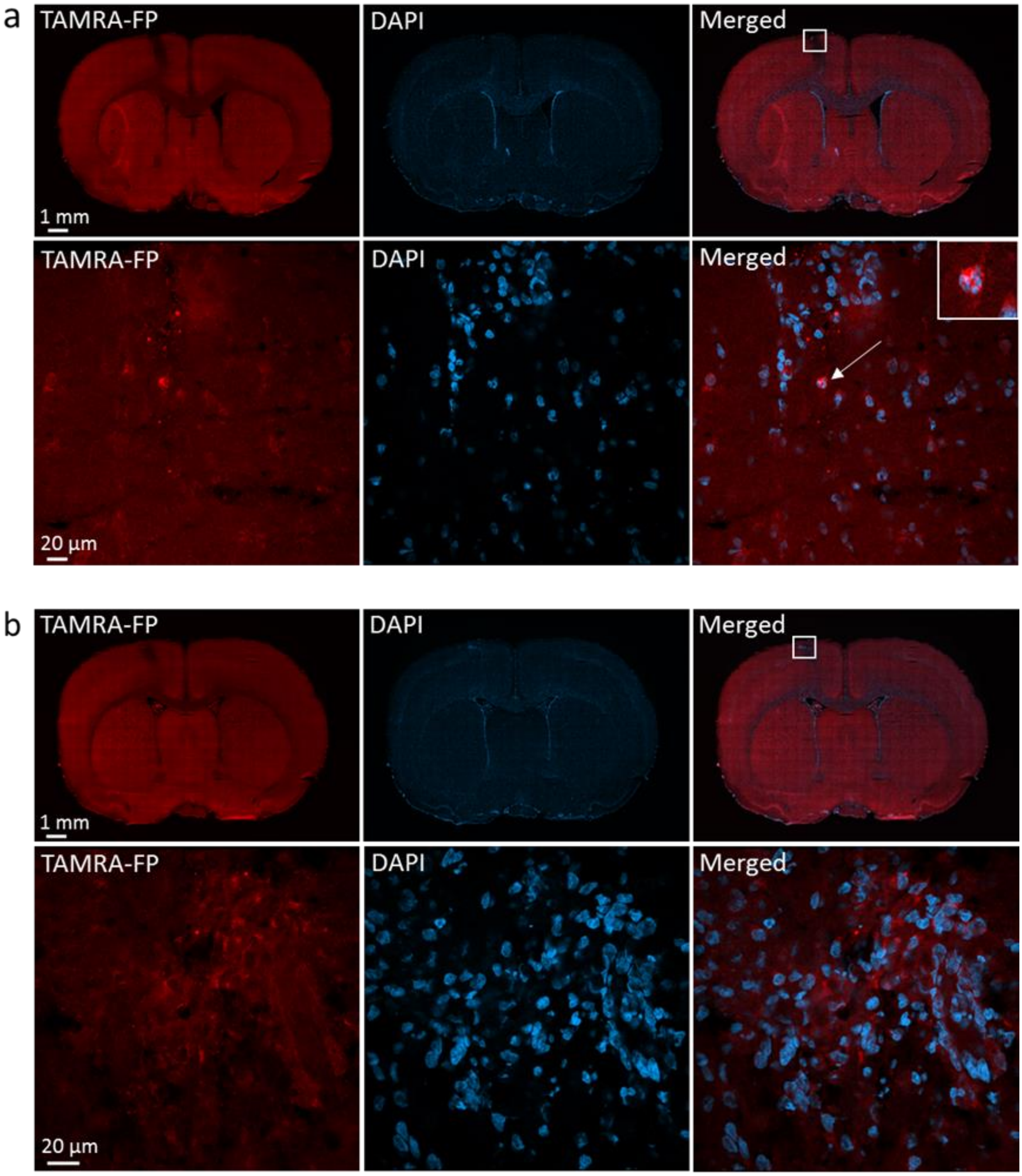

Figure S26. TAMRA-FP signal at the site of injection in sham-operated animals. Sections covering the site of sham-injection (tumor cell medium only) in male rat 23 (**a**) and female rat 9 (**b**) went through the tissue-ABPP protocol to label SHs (red), followed by DAPI staining to visualize nuclei (blue). As a rule, multi-nucleated cells with TAN-like morphology were not evident with the exception of one particular cell showing also TAMRA-FP hotspot characteristics. Scale bar 1 mm (upper panels) and 20 µm (lower panels). Scale bars: 1 mm in a, 20 µm in b-d. Images were adjusted for brightness and contrast.

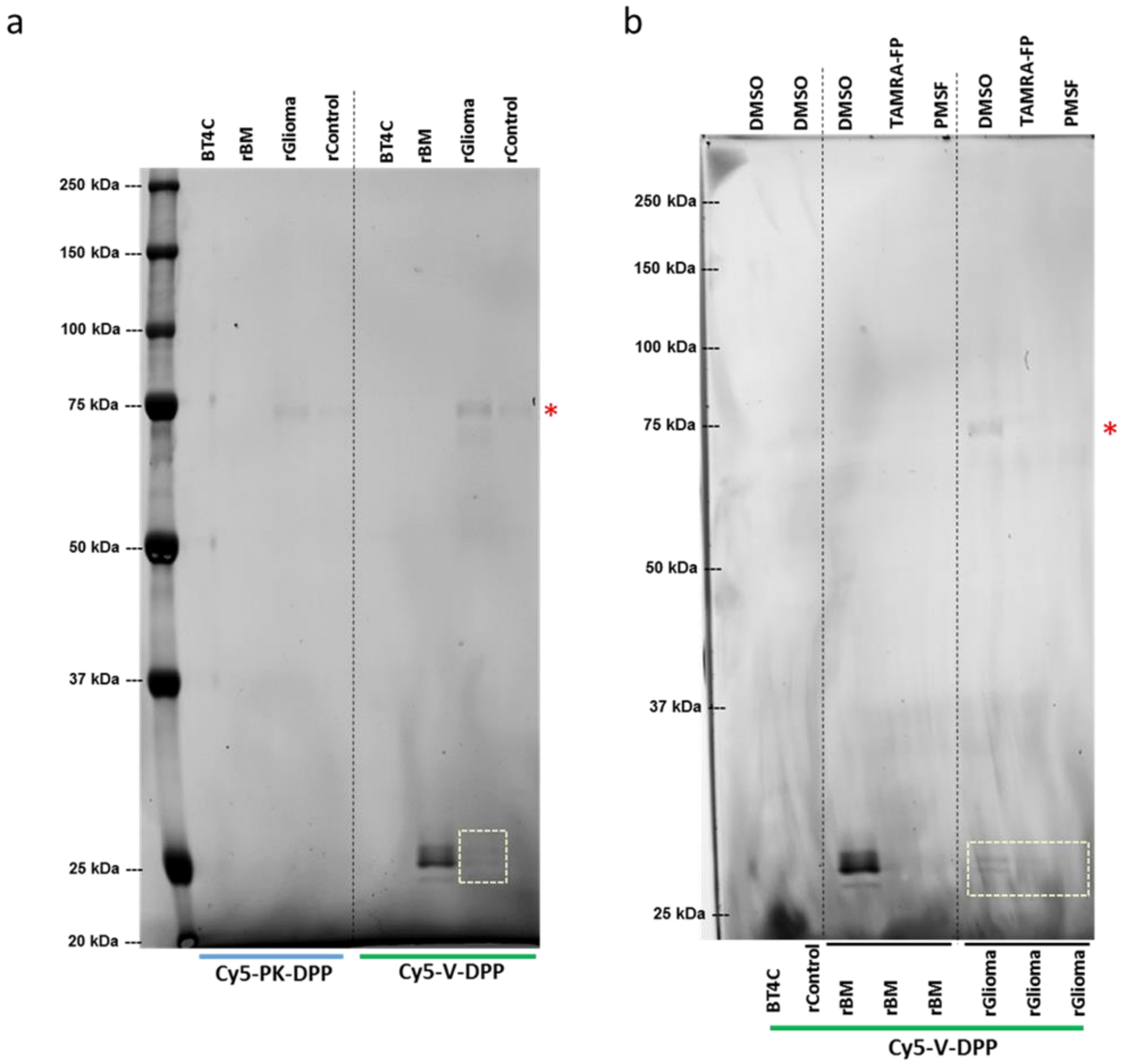

Figure S27. Gel-ABPP of rat glioma proteomes using Cy5-labeled serine protease activity probes PK-DPP and V-DPP. Lysates of BT4C tumor cells, bone-marrow-derived mononuclear cells (rBM) or homogenates of glioma and control brain (all 4 mg/ml) were directly labelled by the indicated Cy5-probes (**a**) or first pretreated for 1h with DMSO or with the SH inhibitors TAMRA-FP (1 µM) or PMSF (1 mM) (**b**), after which the proteomes were labelled by the indicated Cy5-probes for 1h (probe concentration 1 µM). The reaction was quenched, 30 µg protein was loaded per lane, proteins were separated by SDS-PAGE, followed by in-gel fluorescence imaging. MW markers are indicated at left. In **a**, note that the trypsin-preferring probe PK-DPP weakly detects a single ~75 kDa band with unknown identity (red asterisk) in samples of glioma and control brain and that a band of similar size is also weakly recognized by V-DPP. Note that the elastase-preferring probe V-DPP detects 3-4 bands at the ~25 kDa range in rBM and two bands of similar size faintly in the glioma sample (circled by the dotted line). In **b** note that V-DPP labeling of the ~25 kDa protein bands in rBM and glioma (circled by the dotted line) is sensitive to the inhibitors, indicating mutual SH targets for these compounds. Images were adjusted for brightness and contrast. Lanes not related to these probes were removed.

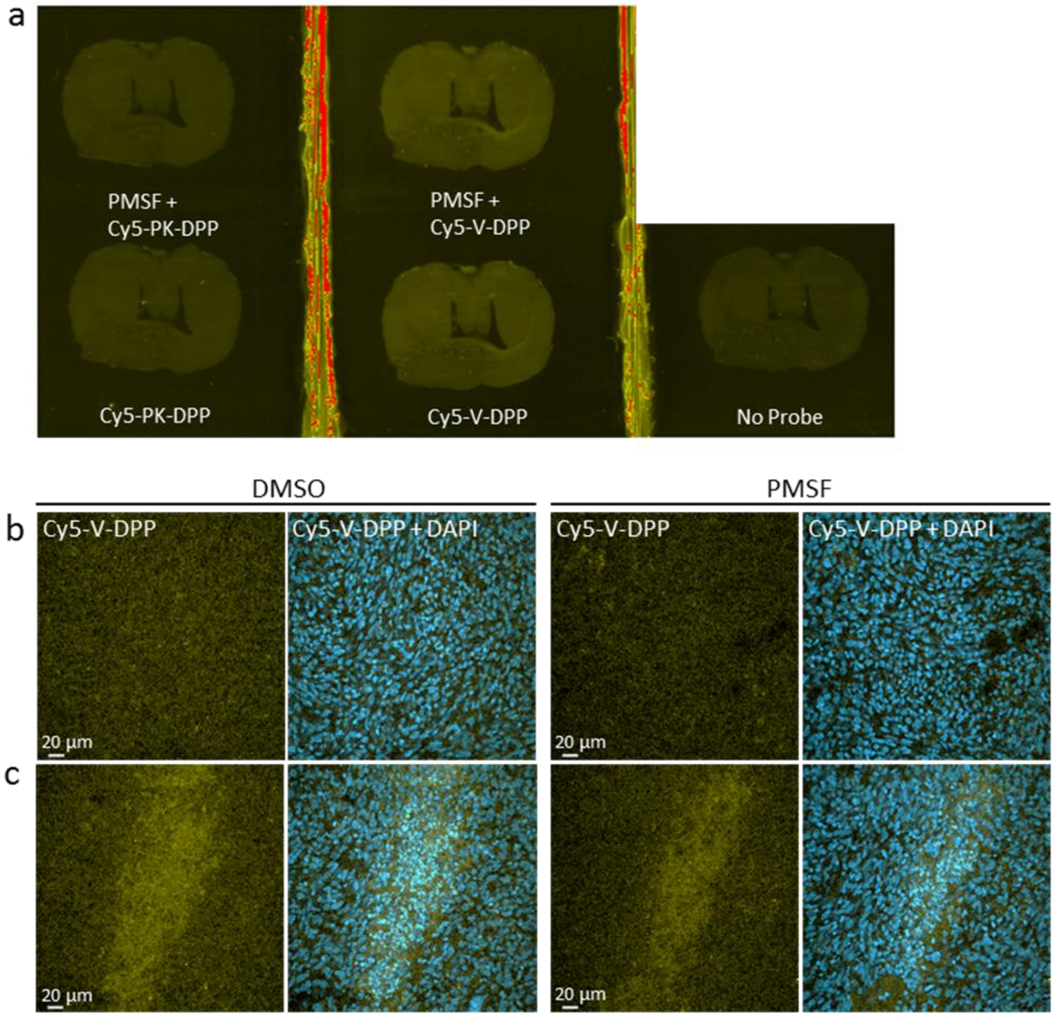

Figure S28. Tissue-ABPP of glioma sections using Cy5-labeled activity probes PK-DPP and V-DPP. Sections were pretreated with solvent (DMSO) or PMSF (1 mM) for 1h at RT, after which they went through the tissue-ABPP protocol using the indicated probes instead of TAMRA-FP (yellow, 0.5 µM final probe concentration), followed by DAPI staining to visualize nuclei (blue). Panel **a** shows overall staining pattern throughout the coronal section plane, imaged using BioRad gel scanner (Cy5 window). A control section undergoing identical protocol with no probe is shown for comparison. Note weak and largely PMSF-resistant signal for both probes. Panel **b** compares Cy5-probe staining pattern between DMSO- and PMSF-pretreated sections in glioma region of TAMRA-FP hotspots. Panel **c** compares Cy5-probe staining pattern between DMSO- and PMSF-pretreated sections in glioma region of TAMRA-FP hotspot clusters. Note largely PMSF-resistant binding of both probes throughout these regions (**b-c**). Images were adjusted for brightness and contrast.

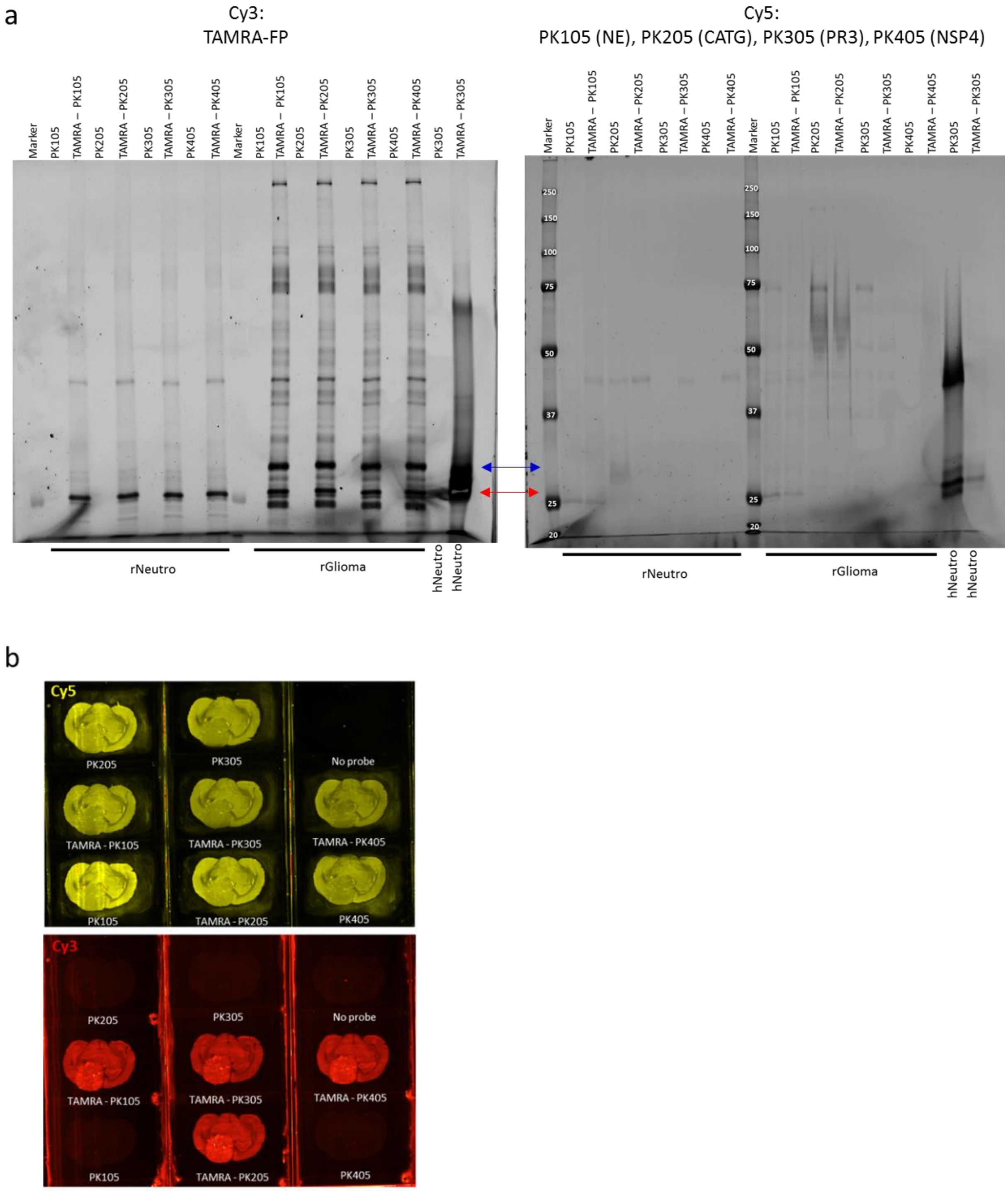

Figure S29. ABPP of rat neutrophil and glioma samples using Cy5-labeled neutrophil serine protease (NSP) probes in combination with TAMRA-FP. In **a**, rat neutrophil lysates (rNeutro, 0.1 mg/ml) or glioma homogenates (1 mg/ml) were treated for 1h with DMSO or with TAMRA-FP (1 µM), after which the proteomes were incubated with the indicated Cy5-probes for 1h (probe concentration 1 µM). Human neutrophil lysate (hNeutro, 0.1 mg/ml) was included as a positive control. The reaction was quenched, 0.7 or 7 µg protein (lysates and glioma homogenate, respectively) was loaded per lane, proteins separated by SDS-PAGE, followed by in-gel fluorescence imaging by BioRad gel scanner using Cy3-window for TAMRA-FP and Cy5-window for the NSP probes. Images of the same gel showing fluorescence in both windows with MW markers indicated in the Cy5-window. Note that TAMRA-FP strongly labels a ~25 kDa band in rat neutrophils (red double-headed arrow) and that band of similar size is also strongly labeled in glioma. Note that the neutrophil ~25 kDa band is not readily detected by any of the NSP probes. Note absence of the prominent ~30 kDa glioma band (blue double-headed arrow) from rat neutrophils. Note also that under the conditions employed, PK305 strongly labels several bands

in the 25-30 kDa range in human neutrophils and that this labeling is sensitive to TAMRA-FP, indicating that the activity probes label the same SH targets. **b.** Tissue-ABPP of glioma sections using Cy5-labeled NSP probes in combination with TAMRA-FP. Sections were pretreated with DMSO or TAMRA-FP (1 µM) for 1h at RT, after which they went through the tissue-ABPP protocol using the indicated probes (0.2 µM final concentration) instead of TAMRA-FP. The upper and lower panels show Cy5- and Cy3-probe labeling pattern, respectively, throughout the coronal section plane, imaged using BioRad gel scanner. A control section undergoing identical protocol with no probe is shown for comparison. Note robust and largely TAMRA-FP-insensitive binding of the Cy5-probes throughout the section. Images were adjusted for brightness and contrast.

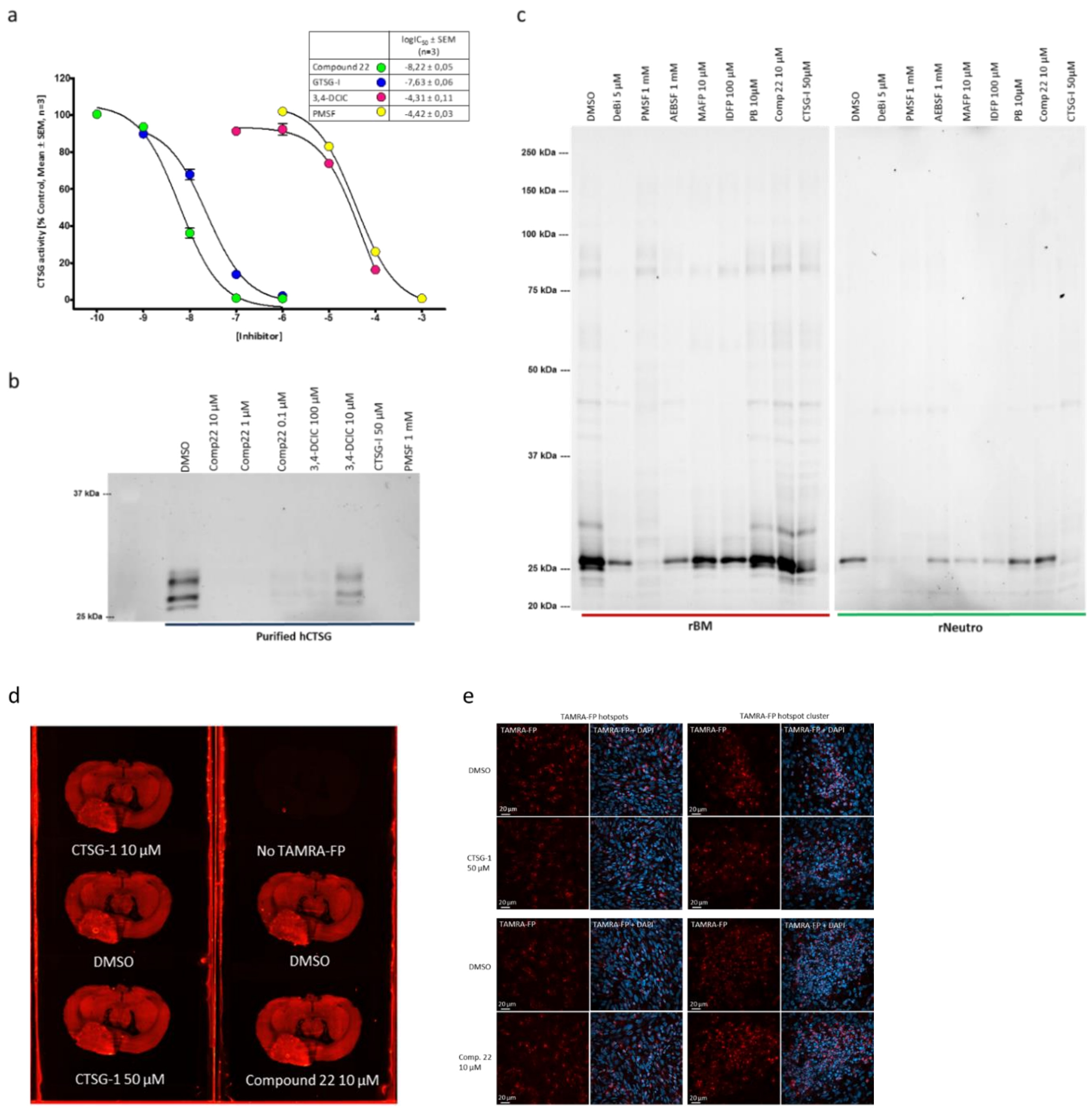

Figure S30. Inhibitor profiles of human cathepsin G (hCTSG) and the prominent 25-30 kDa SH bands in rat bone-marrow-derived mononuclear cells and neutrophils. **a**) Dose-response curves and potencies (logIC_{50} values) for Compound 22 (Cyclo-GTCnXSDPPICFPN), CTSG-Inhibitor (CTSG-I), 3,4-dichloroisocoumarin (3,4-DCIC) and PMSF in inhibiting purified human neutrophil CTSG (Calbiochem Cat# 219373, 100 µU/well). hCTSG-mediated hydrolysis of the chromogenic substrate N-succinyl-Ala-Ala-Pro-Phe-pNA (100 µM) generates 4-nitroaniline whose absorbance was kinetically monitored at $\lambda_{405\ nm}$. The assay contained additionally 0.1 % (w/v) BSA and 1% (v/v) DMSO. **b)** In gel-ABPP, purified hCTSG migrates as four separate bands in the 25-30 kDa range. Note potent inhibition of hCTSG by Compound 22 in line with the outcome of the substrate-based activity assay. **c)** Comparative inhibitor profiling of rat bone-marrow-derived mononuclear cells (rBM) and neutrophils (rNeutro). Note that in contrast to PMSF, Compound 22 does not inhibit 25-30 kDa SH bands in rBM or neutrophils. Inhibitor profiles of the 25-30 kDa SH bands in rBM (1 mg/ml) and the 25 kDa band in neutrophils (0.1 mg/ml) show similar pharmacology, taken into account 10-fold difference in protein-inhibitor stoichiometry (only limited amounts of neutrophils were available for these experiments). **d-e)** Competitive tissue-ABPP of glioma sections pretreated with CTSG inhibitors. Sections were pretreated with DMSO or the indicated concentrations of the CTSG inhibitors for 1h at RT, after which they went through the tissue-ABPP protocol using TAMRA-FP (0.5 µM final concentration). Panel **d**) shows TAMRA-FP labeling pattern throughout the coronal section plane, imaged using BioRad gel scanner and panel **e)** shows confocal fluorescence images of regions with TAMRA-FP hotspots and TAMRA-FP hotspot clusters. Note insensitivity of TAMRA-FP signal throughout hotspots or hotspot clusters towards the CTSG inhibitors. Abbreviations: DeBi, desthiobiotin-FP; PB, palmostatin B. Images were adjusted for brightness and contrast.

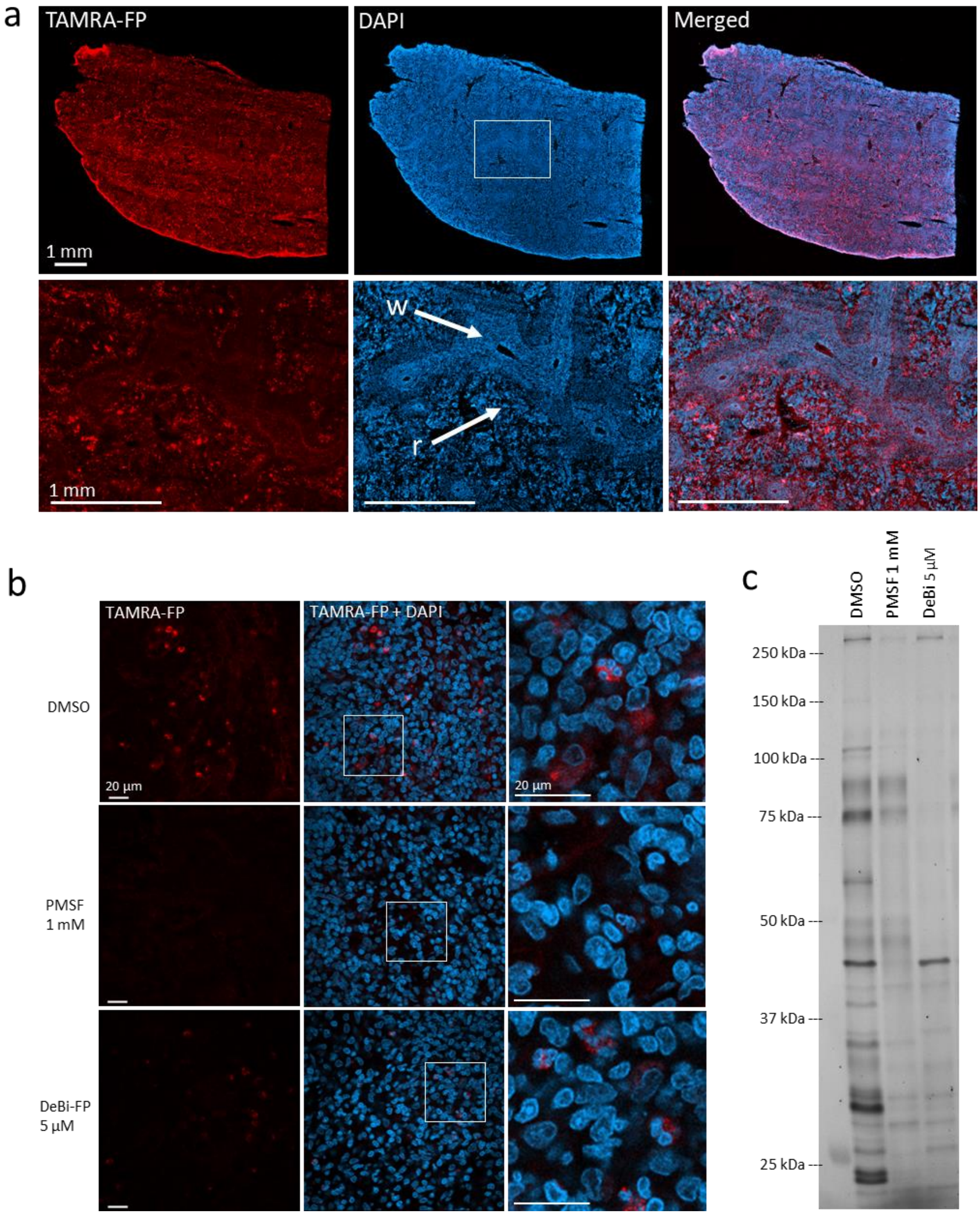

Figure S31. High-resolution imaging of TAMRA-FP hotspots and their inhibitor sensitivity in rat spleen. In **a**, sections went through the tissue-ABPP protocol to label SHs (red), followed by DAPI staining to visualize nuclei (blue). Note presence of TAMRA-FP hotspots in red pulp (r) and absence of TAMRA-FP hotspots from white pulp (w). In **b**, sections were pretreated with DMSO or with the SH inhibitors PMSF (1 mM) or deshiobiotin-FP (DeBi-FP, 5 µM) for 1h at RT, after which they went through the tissue-ABPP protocol to label SHs (red), followed by DAPI staining to visualize nuclei (blue). Red pulp regions of intense TAMRA-FP labeling were imaged. Note presence of multi-nucleated cells in regions of TAMRA-FP hotspots. Note that throughout the examined regions, TAMRA-FP labeling is sensitive to the inhibitors. Gel-ABPP (**c**) using spleen homogenate was run for comparison to visualize TAMRA-FP labeled SH bands and their sensitivity to inhibitors. Note that PMSF is more efficient than DeBi-FP in blocking TAMRA-FP labeling in both tissue- and gel-based ABPP. Scale bars 1 mm (**a**) and 20 µm (**b**). Images were adjusted for brightness and contrast.

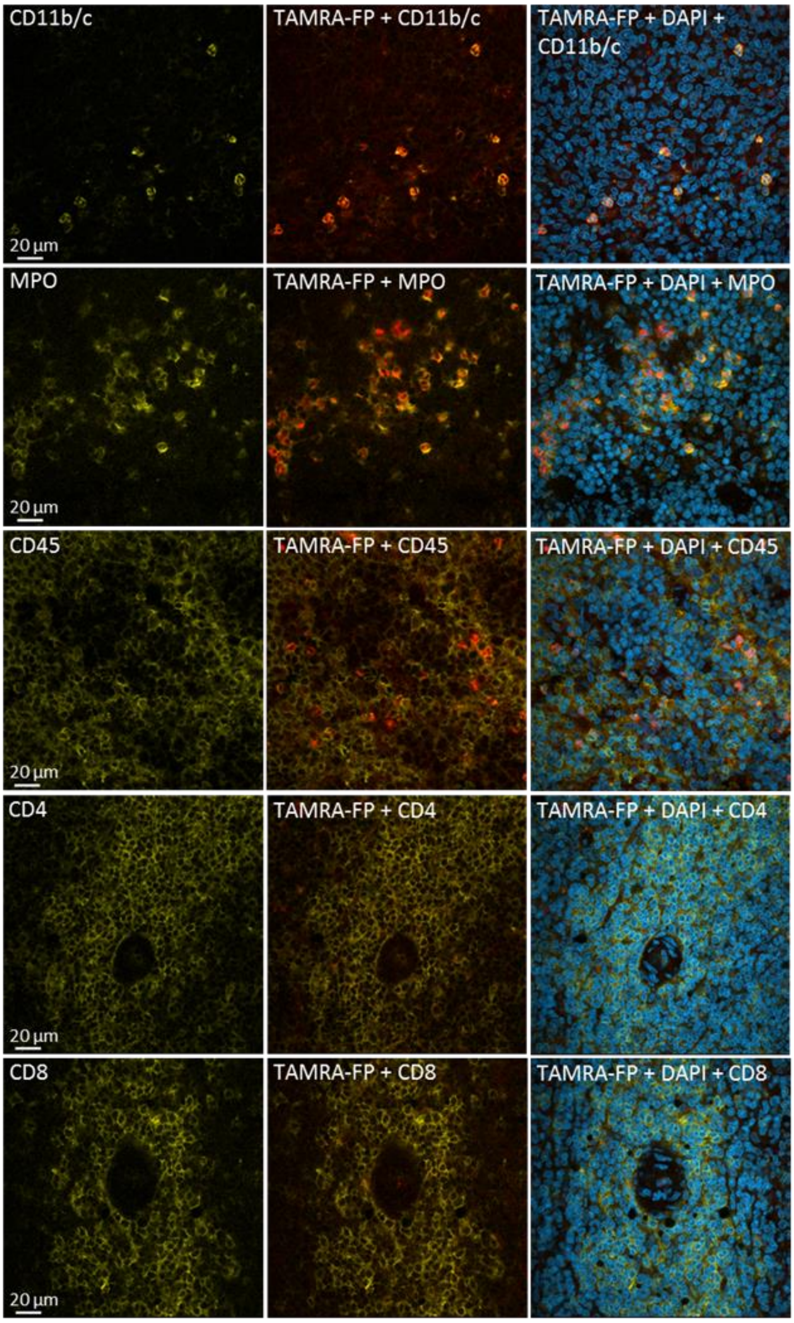

Figure S32. Confocal imaging of SH activity in rat spleen sections in relation to selected immunomarkers. Sections went through the tissue-ABPP protocol to label SHs (red) and were thereafter immunostained for phagocytes (CD11b/c), neutrophils (myeloperoxidase, MPO), nucleated hematopoietic cells (CD45), or T cells (CD4 and CD8) (yellow), followed by DAPI staining to visualize nuclei (blue). Note in particular CD11b/c-positive and MPO-positive cells in the red pulp region of TAMRA-FP hotspots and close match of CD11b/c- and MPO-positive cells with the TAMRA-FP signal. CD45-positive cells partially match with TAMRA-FP signal in red pulp region. CD4- and CD8-positive cells are enriched in white pulp regions with no TAMRA-FP hotspots. Primary antibody mouse anti-CD11b/c (OX42, Abcam, ab1211), dilution 1:500, secondary antibody Donkey anti-mouse IgG-Alexa Fluor 647 conjugate, dilution 1:500; rabbit anti-MPO (Abcam, cat# ab9535), dilution 1:25, secondary antibody Goat anti-rabbit IgG-Alexa Fluor 647 conjugate, dilution 1:500; mouse anti-CD45 (MRC-OX1, Abcam, ab33923, dilution 1:500, secondary antibody Donkey anti-mouse IgG-Alexa Fluor 647 conjugate, dilution 1:100; mouse anti-CD4 (OX-35, Abcam, cat# ab33775), dilution 1:100, secondary antibody Donkey anti-mouse IgG-Alexa Fluor 647 conjugate, dilution 1:100; and mouse anti-CD8 (OX-8, Abcam, cat# ab33786), dilution 1:500, secondary antibody Donkey anti-mouse IgG-Alexa Fluor 647 conjugate, dilution 1:100. Scale bars 20 µm. Images were adjusted for brightness and contrast.

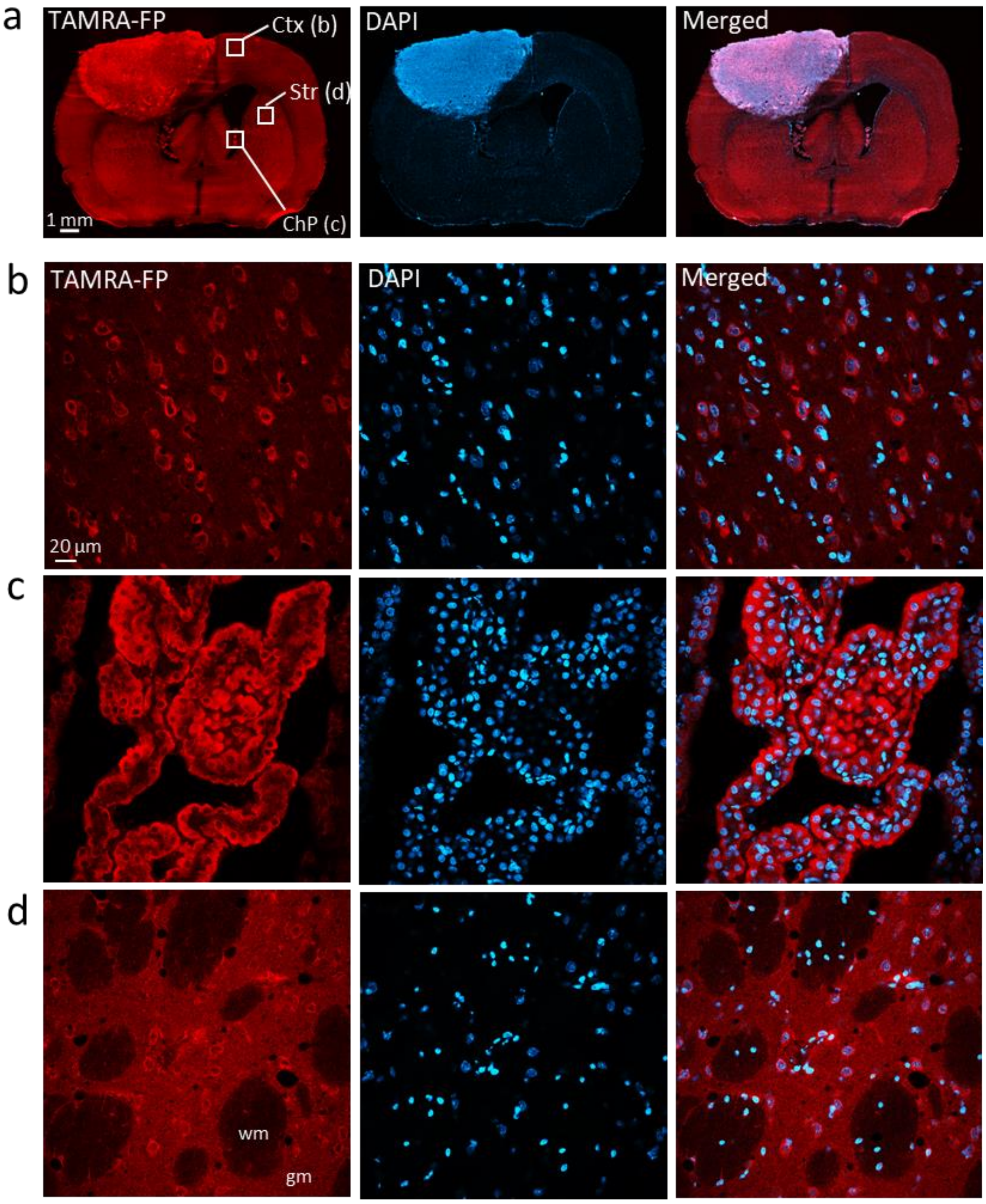

Figure S33. Tissue-ABPP offers sufficient sensitivity to enable imaging of TAMRA-FP fluorescence in regions of the healthy brain. Note clearly detectable TAMRA-FP fluorescence (red) throughout the gray matter regions of healthy brain and less intense signal over the white matter tracts (**a**). Nuclear DAPI staining (blue) indicates dense cell population in glioma as compared to most regions of the healthy brain. **b**) In the cortex (Ctx), TAMRA-FP signal localizes mainly to cytosol and plasma membrane and associates mainly around DAPI stained nuclei. **c)** In the choroid plexus (ChP), relatively intense TAMRA-FP fluorescence associates with DAPI-positive epithelial cells. **d**) In the striatum (Str), TAMRA-FP labeling is more intense in the gray matter (gm) as compared to the white matter (wm). Scale bars: 1 mm in a, 20 µm in b-d. Images were adjusted for brightness and contrast.

References

1. Bachovchin DA, Cravatt BF. The Pharmacological Landscape and Therapeutic Potential of Serine Hydrolases. Nat Rev Drug Discov [Internet]. 2012;11:52–68. Available from: http://www.ncbi.nlm.nih.gov/pmc/articles/PMC3665514/
